# Intersectoral Cooperation in 12 European Case Studies Aiming for Better Health, Environmental Sustainability, and Health Equity: Protocol for a Qualitative Evaluation

**DOI:** 10.2196/17323

**Published:** 2020-06-24

**Authors:** Nina van der Vliet, Lea Den Broeder, María Romeo-Velilla, Hanneke Kruize, Brigit Staatsen, Jantine Schuit

**Affiliations:** 1 National Institute for Public Health and the Environment Bilthoven Netherlands; 2 Tilburg School of Social and Behavioral Sciences Tilburg University Tilburg Netherlands; 3 Achieve Faculty of Health Amsterdam University of Applied Science Amsterdam Netherlands; 4 EuroHealthNet Brussels Belgium

**Keywords:** intersectoral cooperation, health, environmental sustainability, equity, focus groups, protocol

## Abstract

**Background:**

The INHERIT (INtersectoral Health and Environment Research for InnovaTion) project has evaluated intersectoral cooperation (IC) in 12 European case studies attempting to promote health, environmental sustainability, and equity through behavior and lifestyle changes. These factors are the concerns of multiple sectors of government and society. Cooperation of health and environmental sectors with other sectors is needed to enable effective action. IC is thus essential to promote a triple win of health, sustainability, and equity.

**Objective:**

This paper describes the design of a qualitative study to gain insights into successful organization of IC, facilitators and barriers, and how future steps can be taken to improve IC in the evaluated case studies.

**Methods:**

Each case study was assessed qualitatively through a focus group. A total of 12 focus groups in 10 different European countries with stakeholders, implementers, policymakers, and/or citizens were held between October 2018 and March 2019. Five to eight participants attended each focus group. The focus group method was based on appreciative inquiry, which is an asset-based approach focusing on what works well, why it is working well, and how to strengthen assets in the future. A stepped approach was used, with central coordination and analysis, and local implementation and reporting. Local teams were trained to apply a common protocol using a webinar and handbook on organizing, conducting, and reporting focus groups. Data were gathered in each country in the local language. Translated data were analyzed centrally using deductive thematic analysis, with consideration of further emerging themes. Analyses involved the capability, opportunity, motivation-behavior (COM-b) system to categorize facilitators and barriers into capability, motivation, or opportunity-related themes, as these factors influence the behaviors of individuals and groups. Web-based review sessions with representatives from all local research teams were held to check data analysis results and evaluate the stepped approach.

**Results:**

Data collection has been completed. A total of 76 individuals participated in 12 focus groups. In December 2019, data analysis was nearly complete, and the results are expected to be published in fall 2020.

**Conclusions:**

This study proposes a stepped approach that allows cross-country focus group research using a strict protocol while dealing with language and cultural differences. The study generates insights into IC processes and facilitators in different countries and case studies to filter out which facilitators are essential to include. Simultaneously, the approach can strengthen cooperation among stakeholders by looking at future cooperation possibilities. By providing knowledge on how to plan for, improve, and sustain IC successfully to deal with today’s multisectoral challenges, this study can contribute to better intersectoral action for the triple win of better health, sustainability, and equity. This protocol can serve as a tool for other researchers who plan to conduct cross-country qualitative research.

**International Registered Report Identifier (IRRID):**

RR1-10.2196/17323

## Introduction

### Background

Many of today’s behaviors and lifestyles and the drivers that shape them are unhealthy and damaging to the environment. For example, current diet trends involving high meat, fat, and sugar pose a risk to people’s health in terms of overweight and noncommunicable diseases. Furthermore, our global food production system creates a huge pressure on the environment and damages ecosystems [1,2]. However, not all populations are affected equally by health and environmental problems. Changing our behaviors and lifestyles and the environments that shape these behaviors is being progressively acknowledged as vital for not only achieving better health but also creating a more sustainable environment for all [3]. Importantly, population health, environmental sustainability, and equitable health are influenced by factors located in multiple sectors of the government and society. Consequently, addressing current and future challenges of health, environmental sustainability, and equity requires a cross-sectoral approach involving multiple sectors, as not only the challenges themselves but also their solutions are interdependent. Intersectoral cooperation (IC), as an important condition for intersectoral action, allows for these “triple-win” solutions. For example, replacing car journeys with active transport (eg, walking and cycling) is better for both health (through physical exercise) and the environment (through reduced vehicle emissions). In this case, realizing these multisector benefits requires cooperation among the urban planning, environmental, and public health sectors, and between national and local government levels to allow for effective intersectoral action [4]. The importance of IC is recognized internationally [5]. It is believed to only be feasible to achieve the sustainable development goals (SDGs) that aim to achieve a better and more sustainable future for all by 2030, and many countries are committed to this by embracing intersectoral action and cooperation, as is proposed in SDG 17 [6].

We defined IC as cooperation between partners from different sectors, allowing for joint action that is more effective or efficient than actions taken separately by each individual sector. It entails cooperation among parties from different sectors (eg, health and environmental sectors), types of institutions (eg, nongovernmental and private organizations), different levels of government (eg, local and national), and professionals, policy makers, and citizens [7,8].

Previous research has documented the planning, implementation, and evaluation of IC, and the literature shows that working and cooperating intersectorally are not easy processes [8-14]. The barriers mentioned include failing to identify cobenefits, differences in interests, speaking different jargons, siloed ways of thinking, as well as a lack of political will or commitment [8,12,15]. Facilitators identified in the literature include having relationships based on trust and respect, open communication, investing in alliance building, and aiming to achieve consensus at the planning stage of cooperation [8,11,15]. Storm et al studied ways to improve cooperation between the health sector and other sectors in order to reduce health inequalities [16]. Their recommendations included focus on formal cooperation strategies and on working toward higher support for action at tactical and strategic levels.

A wide variety of methods have been applied to study IC. For example, Wagemakers et al developed a coordinated action checklist for community health promotion based on literature and an existing framework, and piloted it sequentially among partnerships in multiple settings [11]. Storm et al used document analysis, questionnaires, and interviews in their study [16]. James et al examined intersectoral policy and action regarding consumer adoption of healthy and sustainable food behaviors by conducting 29 semistructured interviews with key Australian stakeholders [17].

### Aims and Contributions to the Field

#### Intersectoral Cooperation

This protocol paper describes and discusses the design of a qualitative study to gain insights into how IC can be organized successfully, what are the facilitators and barriers to IC, and how future steps could be taken to improve IC in evaluated case studies. Previous evaluation literature on IC mostly focused on health and wellbeing or was nationally oriented. This study can potentially generate new insights as compared with the existing literature, because the cooperation processes of our case studies do not only deal with improving health and wellbeing, but simultaneously aim to promote environmental sustainability and equity. Moreover, in this study, we looked at case studies that cover a diverse range of topics (eg, food consumption, green space, active travel, and energy efficient housing) and are spread out over 10 different European countries. This variety of case studies allows for the generation of insights and perspectives from many different sectors, stakeholders, and countries. The first aim of this study was to gain more insights into processes, facilitators, and barriers of IC and find ways to improve intersectoral action. The results can be used to make generic recommendations and more context, culture, or topic-specific recommendations on what steps to take to effectively organize IC to not only achieve better health and wellbeing for all, but also promote environmental sustainability. In addition, we adopted a qualitative study design using focus groups, as this approach was deemed to be the most suitable for our study; focus groups enable obtaining rich and detailed information about the experiences of the key persons involved in collaborations regarding facilitators and barriers. By using focus groups to study IC in such a wide variety of case studies and countries, we believe that the findings will add to existing literature in which other methods were used or in which focus groups were used to assess national or topic-specific case studies. This can potentially lead to new or additional insights into IC.

#### Stepped Approach

This study aimed to assess IC in 10 different European countries with different cultural backgrounds. Performing such cross-country research poses a methodological challenge, as it requires researchers to know and understand cultural subtleties, language, and behavior in different countries’ contexts. Therefore, the second aim of this study was to pilot a stepped approach that allowed conducting cross-country qualitative research while taking cultural contexts and language barriers into account.

#### Appreciative Inquiry

We applied appreciative inquiry (AI) in our approach, which is an asset-based approach that focuses on what works well, why it is working well, and how to strengthen assets in the future [18,19]. AI has been used successfully in interviews and for the development of a coordinated action checklist to facilitate and evaluate community health promotion partnerships [11,19], and it was found to stimulate participants to appreciate those aspects of cooperation that already exist and inspire them to envision and plan desired future steps in cooperation. Our four core questions were based on AI principles, and we asked participants to discuss (1) how the cooperation began and developed? (2) what and how factors facilitated the cooperation? (3) what were the core barriers and challenges? and (4) how to satisfy future needs and wishes for cooperation?

#### Capability, Opportunity, Motivation-Behavior

**Figure 1:**
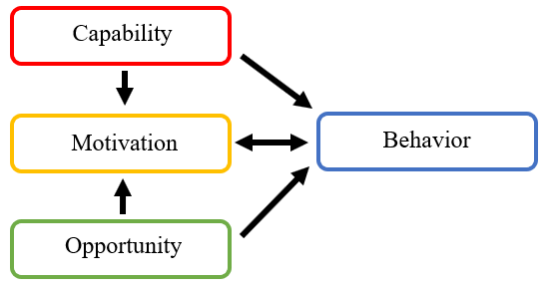
The capability, opportunity, motivation-behavior system. This system is part of the behavioral change wheel and consists of the three behavioral determinants capability, motivation, and opportunity that influence each other and behavior. These determinants can be used to categorize and understand aspects of intersectoral cooperation.

Understanding what works, with whom, and under what circumstances when cooperating intersectorally requires understanding people and their behaviors [20]. The capability, opportunity, motivation-behavior (COM-b) system can be used to understand the behaviors of individuals and groups and was therefore deemed suitable to understand a group of cooperating partners [21]. Capability, opportunity, and motivation are factors that together influence and interact with behavior (Figure 1) [21]. Capability is about being able to perform a certain behavior by having the necessary knowledge and skills. Regarding IC, having good network skills and being able to speak the language or jargon of another sector can be categorized as an aspect of capability [11]. Motivation is about all the brain processes that energize and direct behavior, including more automatic (habits and emotions) and more reflective processes (conscious decision-making). Regarding IC, being motivated to find and work toward a common goal can be categorized as an aspect of motivation, as would be having a positive attitude toward another organization [12]. Opportunity is about having an environment or context that facilitates a certain behavior. Regarding IC, having a work environment that facilitates cooperation can be categorized as an aspect of opportunity. This can include both a social environment (having a boss who stimulates cooperating with other organizations) and a physical environment (in terms of having the necessary resources and time to develop external relationships) [22]. Cooperation involves a group of people and their interactions and behaviors, which are influenced by these behavioral factors, such as their willingness to cooperate and their ability to cooperate, as well as the contexts in which their cooperation takes place and the means available to cooperate (eg, resources [time, budget, etc] and organizational position). The COM-b system has been commonly applied in health promotion (eg, in categorizing healthy food consumption behavior) [23]. Moreover, it has previously been applied in the context of IC by Hendriks et al, who used it to analyze interviews exploring the views of local policy officials on IC [24]. In addition, van Rinsum et al used the COM-b system to identify the types of behaviors of health brokers, who support health promotion in complex public health challenges by facilitating IC [25]. The COM-b system is also part of the conceptual and analytical INtersectoral Health and Environment Research for InnovaTion (INHERIT) model, which was developed to understand health, environmental sustainability, and equity while taking behaviors into account [26]. We applied the COM-b system to analyze focus group data. Applying the COM-b system to study IC using focus groups may provide new insights, as it can highlight factors that influence cooperation (behaviors), which are the most important to develop, improve, and maintain for successful IC in order to allow for triple-win solutions.

#### The INHERIT Project

This study is part of the 4-year (2016-2019) European Union-funded INHERIT project. INHERIT is a research project that aims to understand how lifestyles and behaviors can be changed in order to achieve a triple win, which involves promoting health, environmental sustainability, and equity simultaneously. From the INHERIT project’s promising practices database with over 100 promising practices throughout Europe, INHERIT has identified 15 case studies in the areas of “living” (eg, green areas), “moving” (eg, active transport), and “consuming” (eg, food consumption) that were considered to potentially contribute to the triple win. These were evaluated quantitatively and/or qualitatively, and/or the cost benefit was assessed. IC was studied qualitatively in 12 of these case studies, adding to the quantitative approaches used in the other evaluations. The case studies all involved stakeholders from different sectors that had been cooperating to promote health, environmental sustainability, and equity.

## Methods

### Procedures

#### Central Coordination

**Figure 2:**
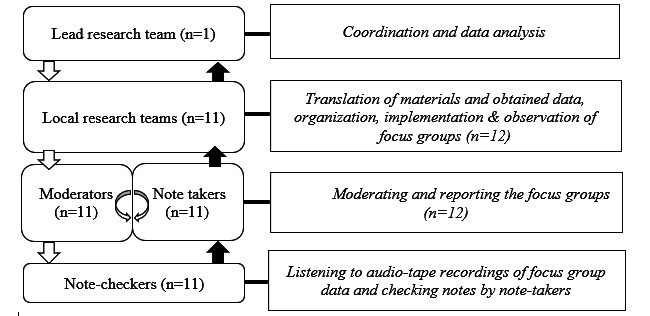
Procedures and roles regarding the focus group process.

Multiple focus groups were conducted from 2018 to 2019 in 10 European countries (Multimedia Appendix 1). This qualitative study involved a stepped design. Figure 2 provides an overview of the procedure and roles of the research teams in each country. One lead research team coordinated the data collection, mostly through group and bilateral teleconferences and emails. In addition, the lead research team also functioned as a local research team for two focus groups conducted in the Netherlands. This resulted in 11 local research teams who conducted the 12 focus groups in their local language. The lead research team provided detailed instructions to standardize procedures and realize similar focus groups while taking into account that these took place in different contexts and were led by heterogenous teams. This was established by providing (1) a webinar to train local research teams on AI and the COM-b model, and to provide practical advice regarding planning, conducting, note taking, and reporting with regard to the focus groups, (2) a detailed handbook with different checklists for local coordinators, moderators, and note takers and information about the COM-b system and AI, (3) a standardized reporting form used by all research teams to report the data from the focus groups, and (4) telephone and email support by the lead team for each local team when needed. At the start, dates were set out for the focus group of each case study. The lead research team ensured that local research teams had received the necessary documents and guidance before starting the planning, conducting, and reporting of the focus groups. For further information on how the local teams were trained and instructed to conduct the focus groups in a similar fashion, the webinar and handbook can be accessed over the internet and in Multimedia Appendix 2 and Multimedia Appendix 3 [27,28].

#### Local Data Collection

One way to investigate IC is through focus groups. O Nyumba et al concluded that a focus group is particularly useful when one’s goal is to generate and evaluate a discussion about a topic that requires collective views and meanings (experiences and beliefs) that lie behind those views [29]. Since we were interested in qualitatively evaluating collective views by cooperation partners on IC, focus groups appeared to be a suitable approach. Thus, each local research team organized one focus group in the respective region of their case study. The moderator was supported by a note taker, who took notes of the focus group discussions using a standardized form provided by the lead research team. Focus group participants were invited to write their views on sticky notes, which were also included in the note-taking form. In addition, all focus group discussions were recorded using an audio recorder (after obtaining permission from the participants). The note-taking form was checked by a second person (who was preferably present as an observer at the focus group), using the audio recordings for reference. Notes could be expanded and corrected by this “note checker.” Any corrected notes were discussed with the note taker to reach an agreement. We focused on IC within the 12 case studies and did not study IC between the different involved countries and case studies.

#### Central Analysis and Checking

Since the focus groups were conducted in native languages, local research teams translated the focus group reports into English before sending it out to the lead research team for analysis. Translated data were anonymized and sent to the lead research team using a secure file sender. Data were stored on a secure server by the lead research team. After the data were analyzed by the lead research team, the local research teams checked the analyses to make sure that the results still reflected focus group outcomes correctly. For this purpose, online review sessions were held with all local research teams to provide feedback on the results and reflect back on the focus groups and to evaluate the used method. This allowed the lead research team to make adjustments if necessary.

### Conducting the Focus Groups

For each focus group, a member of the local research team acted as the focus group moderator. In eight focus groups, a member from the project consortium adopted this role. Only in two cases, the moderator came from an external agency, and this moderator followed the webinar and received the handbook. In addition, a note taker attended the focus group and took notes using a reporting form. Each focus group followed the same time schedule and question structure, with a similar time allocation to each question, allowing for some flexibility (Table 1). Focus groups lasted between 90 and 120 minutes. Participants were given the opportunity to provide informed consent before starting the focus groups. First, participants were asked to introduce themselves. Following AI procedures, these introductions included a warm-up question on what was the most positive experience of the day for each participant attending the focus group. Subsequently, the core questions regarding IC were discussed. Table 1 presents an overview of these questions and time allocation during the focus groups. The core questions regarding success factors and the future of cooperation involved the use of sticky notes to ensure that all participants could provide input. All questions were based on the AI approach [18]. Discussions followed the question structure shown in Table 1 to allow for open discussions that were not guided by already known facilitators and barriers of IC. We believed this could increase the potential to generate new insights. Local research teams were given the option to expand the focus group by a maximum of 30 minutes for additional research questions pertinent to the specific interests of the local research team, which were not related to IC (these data were not analyzed by the lead research team). Immediately after the focus group finished, the moderator and note taker met to discuss outcomes, clarify confusions, and check interpretations.

**Table 1:** Overview of focus group topics and questions.

Topic (time allocation)	Questions
Start and development of the cooperation (an approximately 10-minute discussion)	“How did the cooperation/project start?” “How did it develop to where it is now?” “What contributed to the cooperation process?”
Core (success) factors of the cooperation (an approximately 15-minute discussion)	“What are the core factors that made this cooperation happen and that energized and inspired cooperation?” “Describe a peak experience in (intersectoral) cooperation in [case study X], when you felt really engaged and motivated”
Core barriers, challenges, and missing factors in the cooperation (an approximately 15-minute discussion)	“How could the cooperation have been?” “What would you change if you could change anything in this cooperation? What could it still become?”
Future of the cooperation (an approximately 15-minute discussion)	“Where do you want to be between now and a certain period and what does this future look like? If your dream is X, what would you want to have accomplished in Y years?” “What are possible options (actions and projects) to reach this and enhance cooperation in the future?”
Wrap up and summary by moderator (approximately 5 minutes)	“Of all things discussed, what was the most important to you regarding intersectoral cooperation?”

### Participants

The number of focus groups was set in advance (n=12) owing to strict planning requirements to achieve data collection in 10 different countries. The 12 focus groups consisted of five to eight participants, as this is the ideal size of focus groups for noncommercial topics [30]. In the focus group, at least one policy maker, one implementer of the case study, and a target population representative needed to be present to make sure perspectives from these different groups were represented in the focus group. Together with local case study contact persons, local research teams determined which essential case study stakeholders had to be included in the focus group. All focus group participants should have been involved in the cooperation process.

### Theoretical Framework for Analysis

We used thematic analysis to search for themes and patterns across the data set of the focus groups [31]. Within the thematic analysis, we used a semantic approach to identify themes within explicitly mentioned data and an essentialist or realist approach in which we assumed that language reflects and enables participants to express their experiences. This entails that we assume that the language we use reflects how we give meaning and what we experience [31,32]. Top down (deductive) coding is used; we developed an analytical code tree containing predetermined codes that incorporate our research questions and previously identified elements from existing frameworks in the literature [33]. The code tree is based on the following six conditions for effective IC, as described by Harris et al: necessity, opportunity, capacity, relationships, planned action, and sustained outcomes [9]. Moreover, the codes were developed from other existing literature on success factors and barriers of IC, such as the WHO report on Multisectoral and Intersectoral Action and the Coordinated Action Checklist by Wagemakers et al [8,11]. These factors from previous frameworks were incorporated in our analytical framework, which builds on the INHERIT model and, more specifically, on the COM-b system that is embedded in it [21,26]. The COM-b system (with capability, opportunity, and motivation as interacting determinants of behavior) served as the main structure of our framework, and previously identified IC factors were categorized in one of the COM elements. An explanation of how the COM-b system can help categorize data into codes is provided in the Introduction. In addition, the code tree was structured in accordance with the focus group questions that were inspired by AI [18]. However, further emerging themes that do not fit the analytical framework themes were considered, allowing for new insights.

## Results

Data collection has been completed, and a total of 76 individuals participated in 12 focus groups. In November 2019, data analysis was nearly complete, and the final results are expected to be published in fall 2020. All participants were asked for informed consent beforehand. The study was classified as “exempted from ethical approval” by the Clinical Expertise Centre of the National Institute of Public Health and the Environment.

## Discussion

### Principal Findings

IC is important when looking for triple-win solutions to simultaneously improve health and environmental sustainability and tackle inequity by changing behaviors and lifestyles. In order to develop and implement effective multisector policies and interventions to tackle these challenges, it is important to know which factors contribute to successful cooperation and which factors present barriers. Moreover, it is important to know whether there are similar factors for different settings (eg cultural and national), topics, and types of cooperation, necessitating cross-country research. The approach proposed in this study protocol provides a guideline to conduct cross-country research, allowing the retainment and utilization of local knowledge, while at the same time, striving toward comparable outcomes to combine knowledge on an international cross-cultural level.

This protocol paper proposes centrally organizing coordination and analysis but local organization of 12 focus groups. The use of this approach may allow for better knowledge of local cultural contexts, with local research teams who understand and speak the local language. This may enable local teams to collect more meaningful data, facilitate discussions, and allow capturing concepts and sayings that are culture specific. These advantages were mentioned by local research teams during the web-based review sessions. In addition, local teams are in more direct and close contact with local case study implementers and therefore may know better which cooperation partners should be present at the focus group. In addition, the stepped approach minimizes travelling between the widely spread case studies and allows for a relatively resource-efficient way of conducting international focus group research, while incorporating predefined data-checking steps to ensure data quality.

We use an approach inspired by AI, which is an asset-based approach that focuses on what works well and how to do more of it in the future [18]. A common criticism on AI is that it ignores issues and problems. However, there is room for negative experiences, and practice has shown that these do emerge when using AI, but they are dealt with from a reframed perspective. Participants are asked to think about what they are missing, what created the gap between what they see and what they want to see, and how to close that gap instead of dwelling on these negative experiences [34,35]. AI fits our combined aims of generating knowledge and further improving IC processes well. Moreover, although the case studies are evaluated in different contexts and languages and by different moderators, AI principles are relatively easy to implement, which could partly address the limitation of having many different local research teams conducting the focus groups. AI facilitated similarity in local approaches as executing it is quite straightforward. This was confirmed by the review sessions with local teams, who mentioned the ease of working with AI principles when conducting the focus groups. Moreover, from these review sessions, there were indications that the AI approach is particularly useful in more hierarchical situations to have open and equal conversations between partners.

### Limitations

A limitation of this approach may be that the researcher conducting data analysis was not the note taker and was not present at all the focus groups, as this was not feasible owing to language barriers. Original focus group notes had to be translated into English by local research teams, which might have caused some richness of data to be lost. If the focus groups had been conducted in English by the lead research team to gain more compatible data, it would have resulted in exclusion of the possibility of evaluation in some countries with case studies owing to the lack of mastery of the English language. Moreover, it could have resulted in an overrepresentation of participants with higher education and misrepresentation of different socioeconomic status backgrounds. In addition, this would have led to a loss in data richness and misunderstandings, as participants were nonnative English speakers in 11 focus groups. To partly overcome misrepresentation of data that could arise by translation and central analysis, the analysis results were checked by those who were present at the focus group (either an observer or the note taker).

The number of focus groups was set in advance owing to project requirements. This restricted theoretical sampling opportunities to achieve data saturation, where new information from data collection and analysis produces little to no change in the codebook [36]. However, a recent study found that 80% of themes among 40 focus groups were already discovered in two to three focus groups, confirming earlier literature on the relatively small number of focus groups or interviews needed to achieve data saturation [37].

An additional limitation of this study may be that although the notes taken during focus groups were checked and expanded afterwards with the audiotape recordings, no verbatim transcripts and translations were available owing to limited budgets. To allow for this cross-country stepped approach, it was decided to use budgets for translation and note taking with checking of audio recordings. More importantly, although transcription has been considered the “gold standard” in qualitative research, this combination of note taking and using audio recordings allows for the comparison of notes to actual responses and helps fill in blank spaces in field notes [38]. Moreover, as Halbcomb indicated, in the case of thematic analysis in which common themes are sought, verbatim transcription is not always necessary [38]. This author refers to several other authors who state that verbatim transcription is just one of the methods to capture verbal data [39]. Note taking with a check using audio recordings of the focus groups was therefore deemed sufficient in the context of this cross-country study, allowing data collection in local languages.

### Comparison With Prior Work

IC has been studied by previous researchers [8-14]. However, a great part of the resulting literature centers around promotion of health and well-being, while some literature looks at IC or action to improve both health and environmental sustainability and other literature looks at health inequalities. Our study focuses on the combination of simultaneously promoting health and environmental sustainability and addressing inequities, which may lead to new insights as a wider variety of sectors are involved. In addition, while previous research often focused on a specific topic, our case studies center around a diverse range of topics from healthy and sustainable food consumption to active travel by cycling and from energy efficiency to green space. Previous methods to study IC mainly included interviews and literature reviews. We decided to study IC by means of focus groups, as this approach allows us to evaluate collective views in groups of cooperation partners and generate discussion and future plans among the cooperation partners [29]. In addition, we used the COM-b system to categorize and structure data analysis. The COM-b system is a relatively simple behavioral model consisting of three factors that influence and interact with behavior. To our knowledge, the COM-b system has not been applied previously to analyze focus group data on IC, and this can lead to new insights and recommendations regarding what is needed for individuals and groups to practice effective IC in terms of capability, opportunity, and motivation. The results of this study will contribute to evidence on whether COM-b is a useful model to apply for group behaviors, such as IC.

### Conclusions

To our knowledge, no other qualitative study has been conducted in a similar manner to evaluate IC in a diverse range of European interventions in order to achieve the aforementioned triple-win solutions. Performing this type of cross-country research requires a strict approach, and our protocol can serve as a tool for other researchers who plan to conduct this type of research. The results of our study will demonstrate how the COM-b system can contribute to understanding the conditions for behavior that are necessary to develop and maintain successful IC. In addition, the applied stepped approach can be used by other researchers who wish to conduct focus groups in cross-country research. Insights from this qualitative evaluation will be used as one of INHERIT’s input sources for the development of a policy toolkit that will help and inform policy makers on actions that can lead to a healthier, more environmentally sustainable, and more equitable future. Often, approaches that work in one country or context do not necessarily work in another country or context. This study will provide an overview of key elements of successful cooperation according to stakeholders who cooperate in the context of a wide variety of European case studies. In addition, this study will generate rich data as it allows for comparison among a broad variety of interventions that enhance health, environmental sustainability, and equity by means of behavioral or lifestyle change, but differ strongly in terms of topic, cultures, and contexts. Therefore, the study can provide valuable lessons about what works when engaging in IC, and whether and how it differs among contexts (political, social, and cultural) for a broad set of topics.

## Appendix

Multimedia Appendix 1 Overview of the 12 case studies, with name, country, and a short description.

Multimedia Appendix 2 Webinar for qualitative evaluation (INHERIT).

Multimedia Appendix 3 Handbook for qualitative evaluation (INHERIT).

## Abbreviations

AI: appreciative inquiry
COM-b: capability, opportunity, motivation-behavior
IC: intersectoral cooperation
INHERIT: INtersectoral Health and Environment Research for InnovaTion
SDG: sustainable development goal

## References

[1] Tilman D, Clark M (2014). Global diets link environmental sustainability and human health. Nature.

[2] Willett W, Rockström J, Loken B, Springmann M, Lang T, Vermeulen S, Garnett T, Tilman D, DeClerck F, Wood A, Jonell M, Clark M, Gordon LJ, Fanzo J, Hawkes C, Zurayk R, Rivera JA, De Vries W, Majele Sibanda L, Afshin A, Chaudhary A, Herrero M, Agustina R, Branca F, Lartey A, Fan S, Crona B, Fox E, Bignet V, Troell M, Lindahl T, Singh S, Cornell SE, Srinath Reddy K, Narain S, Nishtar S, Murray CJ (2019). Food in the Anthropocene: the EAT-Lancet Commission on healthy diets from sustainable food systems. Lancet.

[3] Aibana K, Kimmel J, Welch S, United Nations (2017). Consuming differently, consuming sustainably: behavioural insights for policymaking.

[4] Staatsen B, van der Vliet N, Kruize H, Hall L, Morris G, Bell R, Stegeman I (2017). INHERIT: Exploring triple-win solutions for living, moving and consuming that encourage behavioural change, protect the environment, promote health and health equity.

[5] Kickbusch I, Gleicher D (2012). Governance for health in the 21st century.

[6] United Nations General Assembly (2015). Transforming our world: The 2030 agenda for sustainable development.

[7] Kirch W (2008). Intersectoral cooperation. Encyclopedia of Public Health. Vol 2019.

[8] Tiliouine A, WHO Regional Office for Europe (2018). Multisectoral and intersectoral action for improved health and well-being for all: mapping of the WHO European Region. Governance for a sustainable future: improving health and well-being for all.

[9] Harris E, Wise M, Hawe P, Finlay P, Nutbeam D (1995). Working together: intersectoral action for health.

[10] Health Canada (2000). Intersectoral action toolkit: The cloverleaf model for success.

[11] Wagemakers A, Koelen MA, Lezwijn J, Vaandrager L (2010). Coordinated action checklist: a tool for partnerships to facilitate and evaluate community health promotion. Glob Health Promot.

[12] Danaher A (2011). Reducing Health Inequities: Enablers and Barriers to Inter-sectoral Collaboration.

[13] Storm I (2016). Towards a HiAP cycle: Health in All Policies as a practice-based improvement process. Research VU.

[14] Graham WJ, Kuruvilla S, Hinton R, Veitch E, Simpson PJ (2018). Multisectoral collaboration for health and sustainable development. BMJ.

[15] Public Health Agency of Canada, Health Systems Knowledge Network, EQUINET (2017). Crossing Sectors - Experiences in Intersectoral Action, Public Policy and Health.

[16] Storm I, den Hertog F, van Oers H, Schuit AJ (2016). How to improve collaboration between the public health sector and other policy sectors to reduce health inequalities? - A study in sixteen municipalities in the Netherlands. Int J Equity Health.

[17] James SW, Friel S, Lawrence MA, Hoek AC, Pearson D (2017). Inter-sectoral action to support healthy and environmentally sustainable food behaviours: a study of sectoral knowledge, governance and implementation opportunities. Sustain Sci.

[18] Cooperrider DL, Whitney D, Stavros JM (2003). Appreciative Inquiry Handbook: The First in a Series of AI Workbooks for Leaders of Change.

[19] Michael S (2005). The promise of appreciative inquiry as an interview tool for field research. Development in Practice.

[20] No authors listed (2018). The cooperative human. Nat Hum Behav.

[21] Michie S, van Stralen MM, West R (2011). The behaviour change wheel: a new method for characterising and designing behaviour change interventions. Implement Sci.

[22] Corbin JH, Jones J, Barry MM (2018). What makes intersectoral partnerships for health promotion work? A review of the international literature. Health Promot Int.

[23] Graça J, Godinho CA, Truninger M (2019). Reducing meat consumption and following plant-based diets: Current evidence and future directions to inform integrated transitions. Trends in Food Science & Technology.

[24] Hendriks A, Jansen MW, Gubbels JS, De Vries NK, Molleman G, Kremers SP (2015). Local government officials׳ views on intersectoral collaboration within their organization – A qualitative exploration. Health Policy and Technology.

[25] van Rinsum CE, Gerards SM, Rutten GM, van de Goor IA, Kremers SP (2017). Health Brokers: How Can They Help Deal with the Wickedness of Public Health Problems?. Biomed Res Int.

[26] van der Vliet N, Staatsen B, Kruize H, Morris G, Costongs C, Bell R, Marques S, Taylor T, Quiroga S, Martinez Juarez P, Máca V, Ščasný M, Zvěřinová I, Tozija F, Gjorgjev D, Espnes GA, Schuit J (2018). The INHERIT Model: A Tool to Jointly Improve Health, Environmental Sustainability and Health Equity through Behavior and Lifestyle Change. Int J Environ Res Public Health.

[27] RIVM (2019). INHERIT.

[28] RIVM (2019). INHERIT.

[29] O Nyumba T, Wilson K, Derrick CJ, Mukherjee N (2018). The use of focus group discussion methodology: Insights from two decades of application in conservation. Methods Ecol Evol.

[30] Krueger RA, Casey MA (2014). Focus groups: a practical guide for applied research.

[31] Braun V, Clarke V (2006). Using thematic analysis in psychology. Qualitative Research in Psychology.

[32] Potter J, Wetherell M (1987). Discourse and social psychology: Beyond attitudes and behaviour.

[33] Boyatzis R (1998). Thematic analysis and code development: Transforming qualitative information.

[34] Coghlan A, Preskill H, Tzavaras Catsambas T (2003). An overview of appreciative inquiry in evaluation. New Directions for Evaluation.

[35] Bushe GR (2007). OD Practioner.

[36] Guest G, Bunce A, Johnson L (2016). How Many Interviews Are Enough?. Field Methods.

[37] Guest G, Namey E, McKenna K (2016). How Many Focus Groups Are Enough? Building an Evidence Base for Nonprobability Sample Sizes. Field Methods.

[38] Halcomb EJ, Davidson PM (2006). Is verbatim transcription of interview data always necessary?. Appl Nurs Res.

[39] Tessier S (2012). From Field Notes, to Transcripts, to Tape Recordings: Evolution or Combination?. International Journal of Qualitative Methods.

