# Supplementary material for: Intersectoral Cooperation in 12 European Case Studies Aiming for Better Health, Environmental Sustainability, and Health Equity: Protocol for a Qualitative Evaluation
Source: JMIR Res Protoc. 2020 Jun 24;9(6):e17323. doi: 10.2196/17323 (PMC7381080; doi:10.2196/17323)
Supplement: Multimedia Appendix 1 [file resprot_v9i6e17323_app1.docx]

**Appendix 1** Overview of the 12 case studies, with name, country and short description.
