# Supplementary material for: Intersectoral Cooperation in 12 European Case Studies Aiming for Better Health, Environmental Sustainability, and Health Equity: Protocol for a Qualitative Evaluation
Source: JMIR Res Protoc. 2020 Jun 24;9(6):e17323. doi: 10.2196/17323 (PMC7381080; doi:10.2196/17323)
Supplement: Multimedia Appendix 2 [file resprot_v9i6e17323_app2.pptx]

## Slide 1
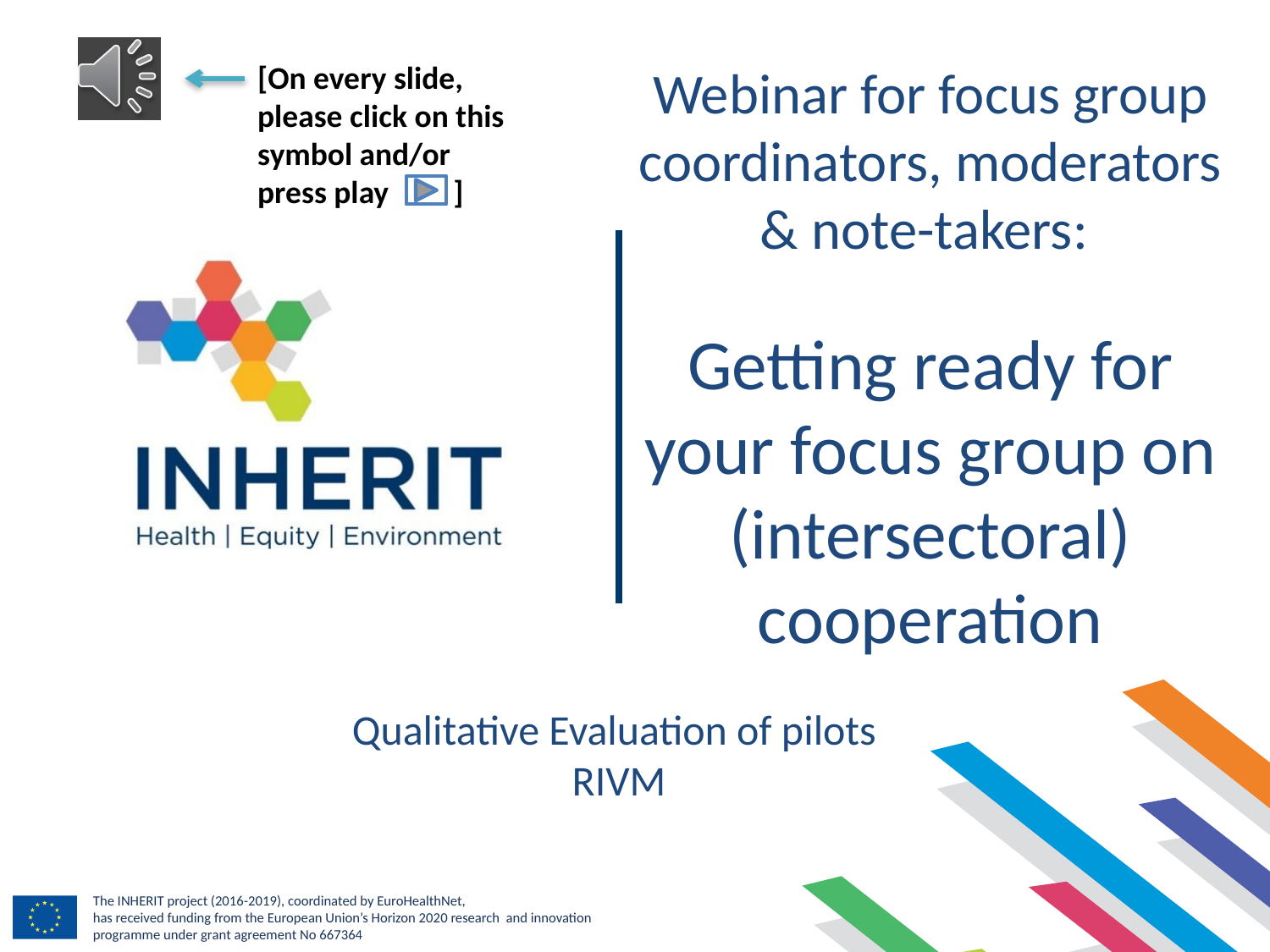

[On every slide, please click on this symbol and/or press play ]
Webinar for focus group coordinators, moderators & note-takers:
Getting ready for your focus group on (intersectoral) cooperation
Qualitative Evaluation of pilots
RIVM
The INHERIT project (2016-2019), coordinated by EuroHealthNet,
has received funding from the European Union’s Horizon 2020 research and innovation programme under grant agreement No 667364

## Slide 2
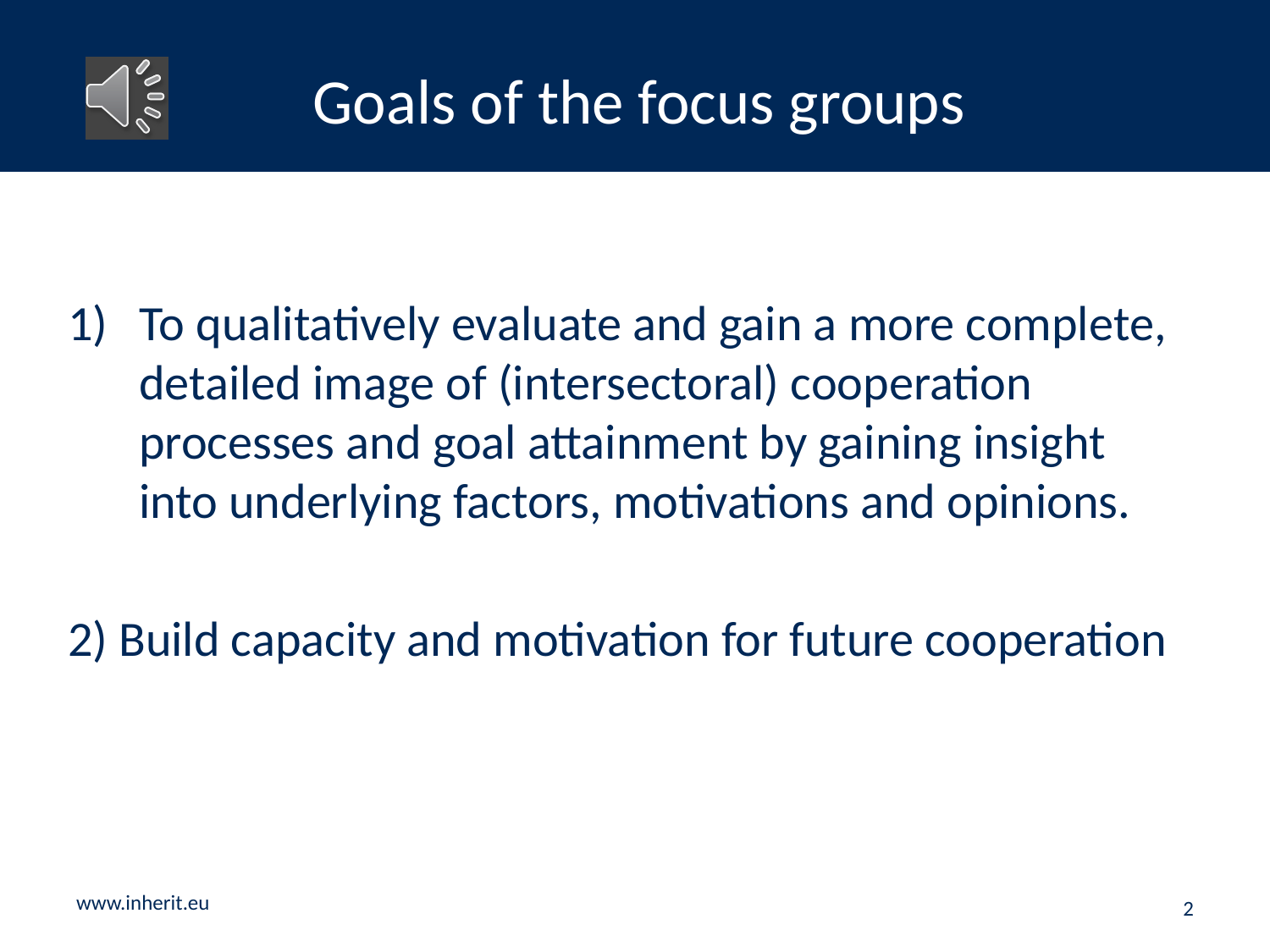

# Goals of the focus groups
To qualitatively evaluate and gain a more complete, detailed image of (intersectoral) cooperation processes and goal attainment by gaining insight into underlying factors, motivations and opinions.
2) Build capacity and motivation for future cooperation
www.inherit.eu
2

## Slide 3
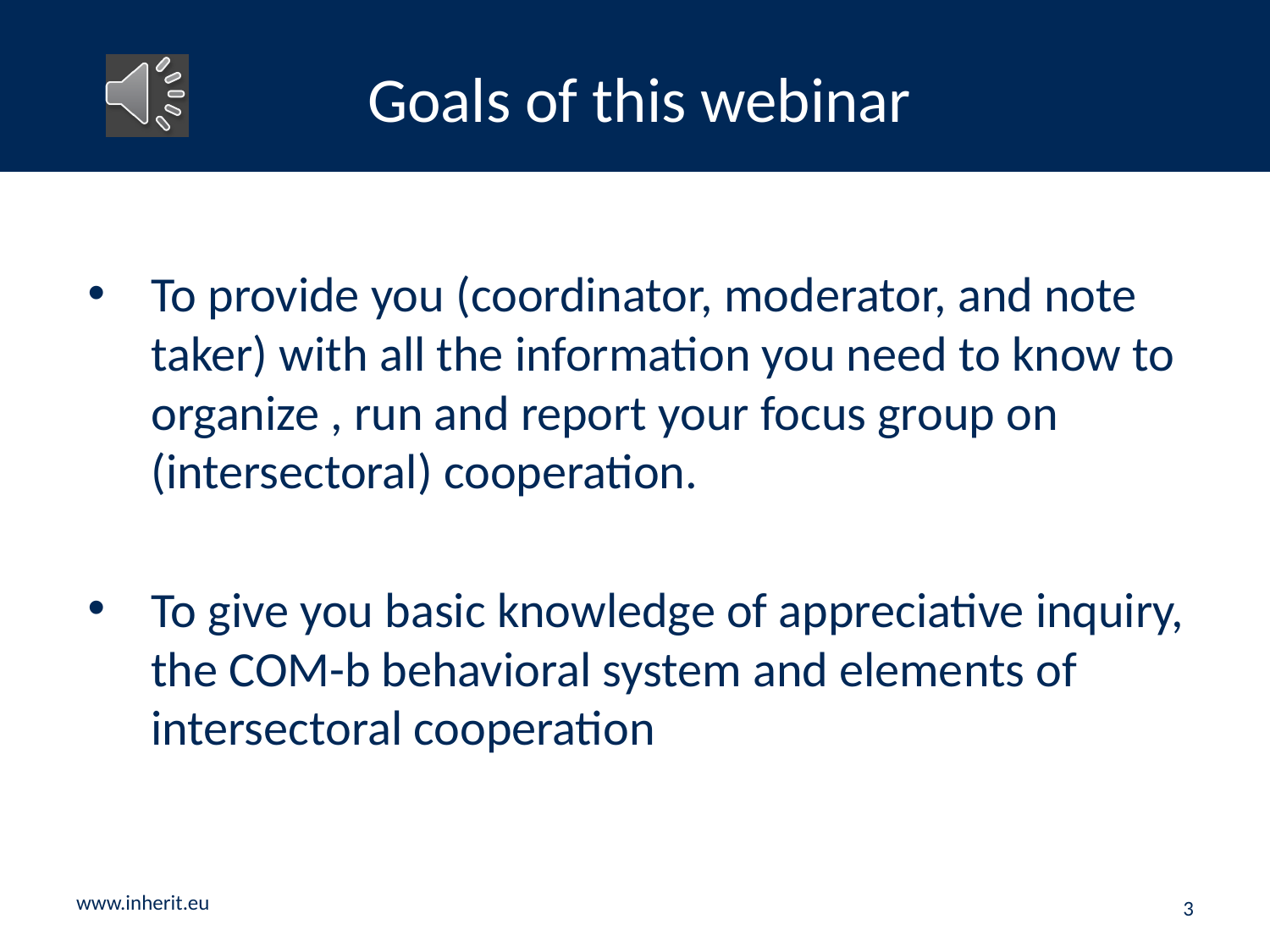

# Goals of this webinar
To provide you (coordinator, moderator, and note taker) with all the information you need to know to organize , run and report your focus group on (intersectoral) cooperation.
To give you basic knowledge of appreciative inquiry, the COM-b behavioral system and elements of intersectoral cooperation
www.inherit.eu
3

## Slide 4
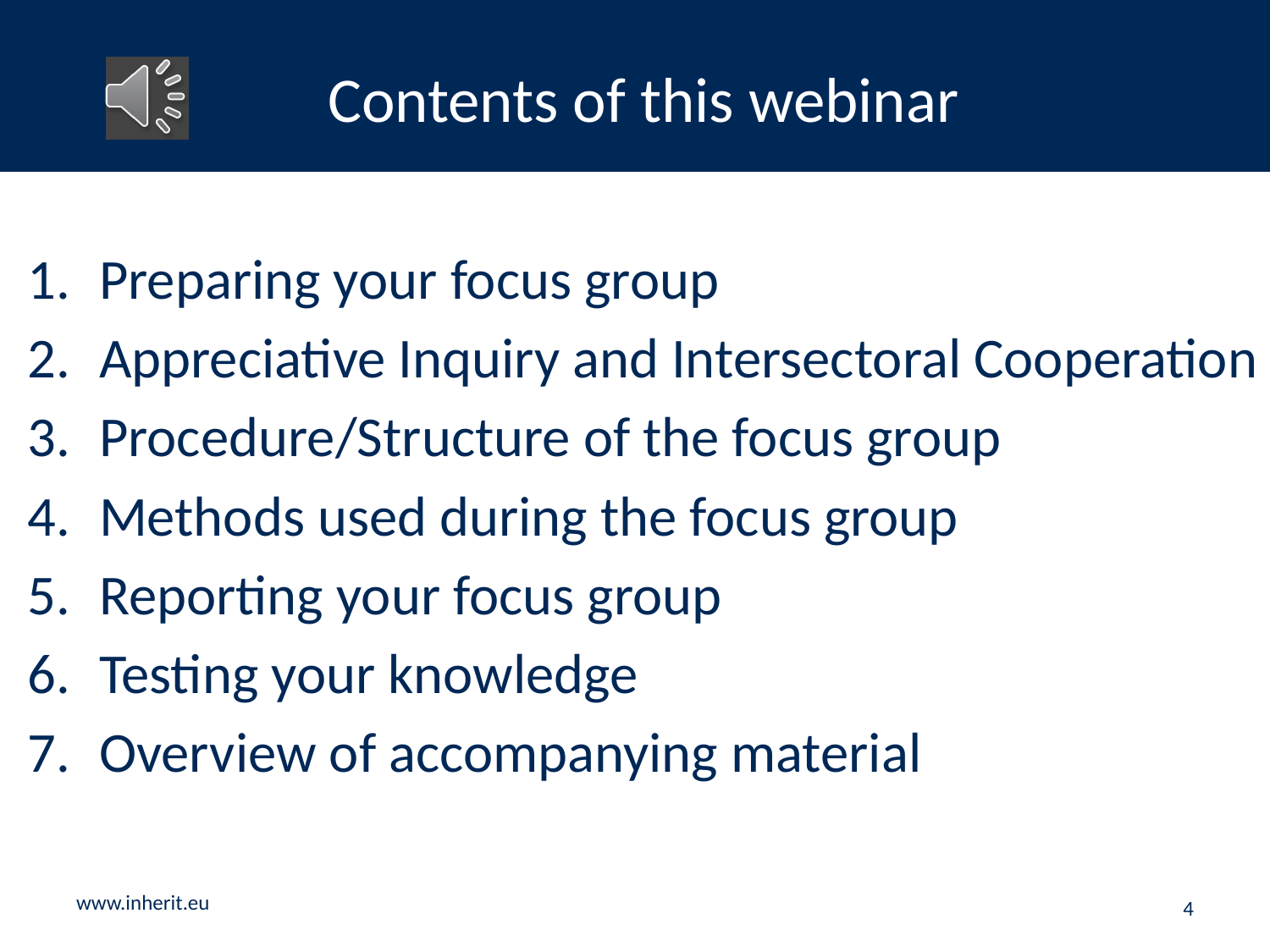

# Contents of this webinar
Preparing your focus group
Appreciative Inquiry and Intersectoral Cooperation
Procedure/Structure of the focus group
Methods used during the focus group
Reporting your focus group
Testing your knowledge
Overview of accompanying material
www.inherit.eu
4

## Slide 5
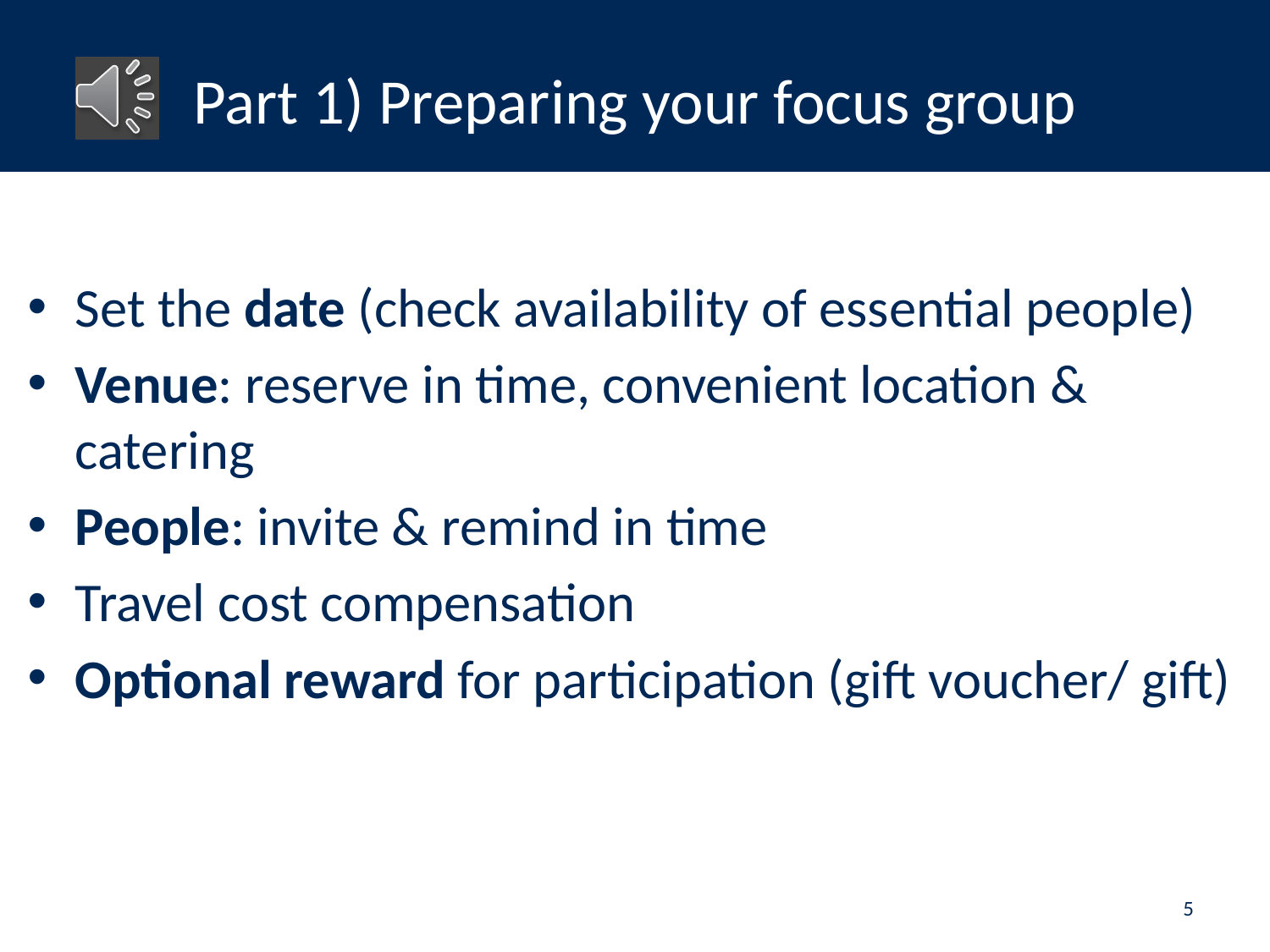

# Part 1) Preparing your focus group
Set the date (check availability of essential people)
Venue: reserve in time, convenient location & catering
People: invite & remind in time
Travel cost compensation
Optional reward for participation (gift voucher/ gift)
5

## Slide 6
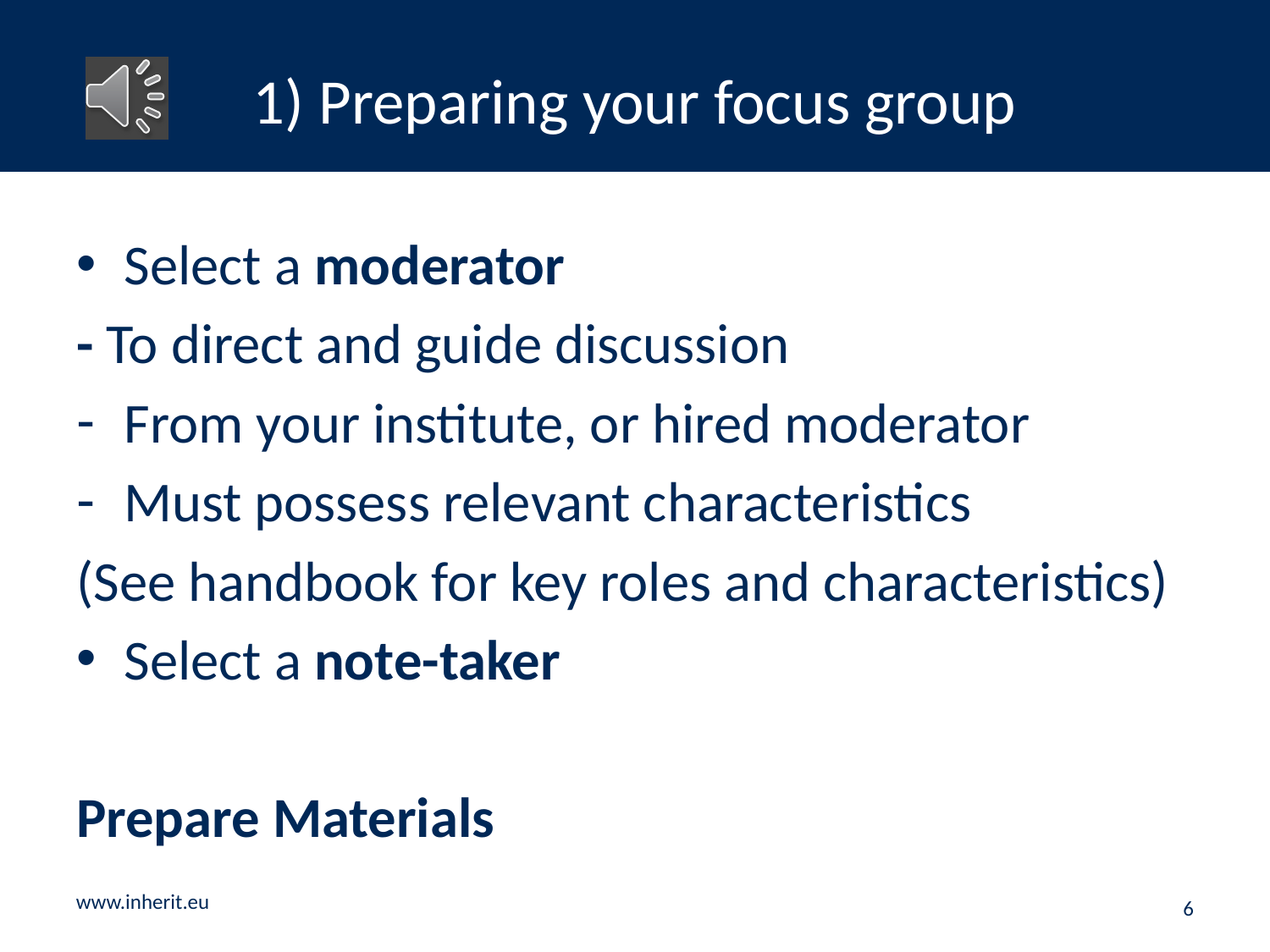

# 1) Preparing your focus group
Select a moderator
- To direct and guide discussion
From your institute, or hired moderator
Must possess relevant characteristics
(See handbook for key roles and characteristics)
Select a note-taker
Prepare Materials
www.inherit.eu
6

## Slide 7
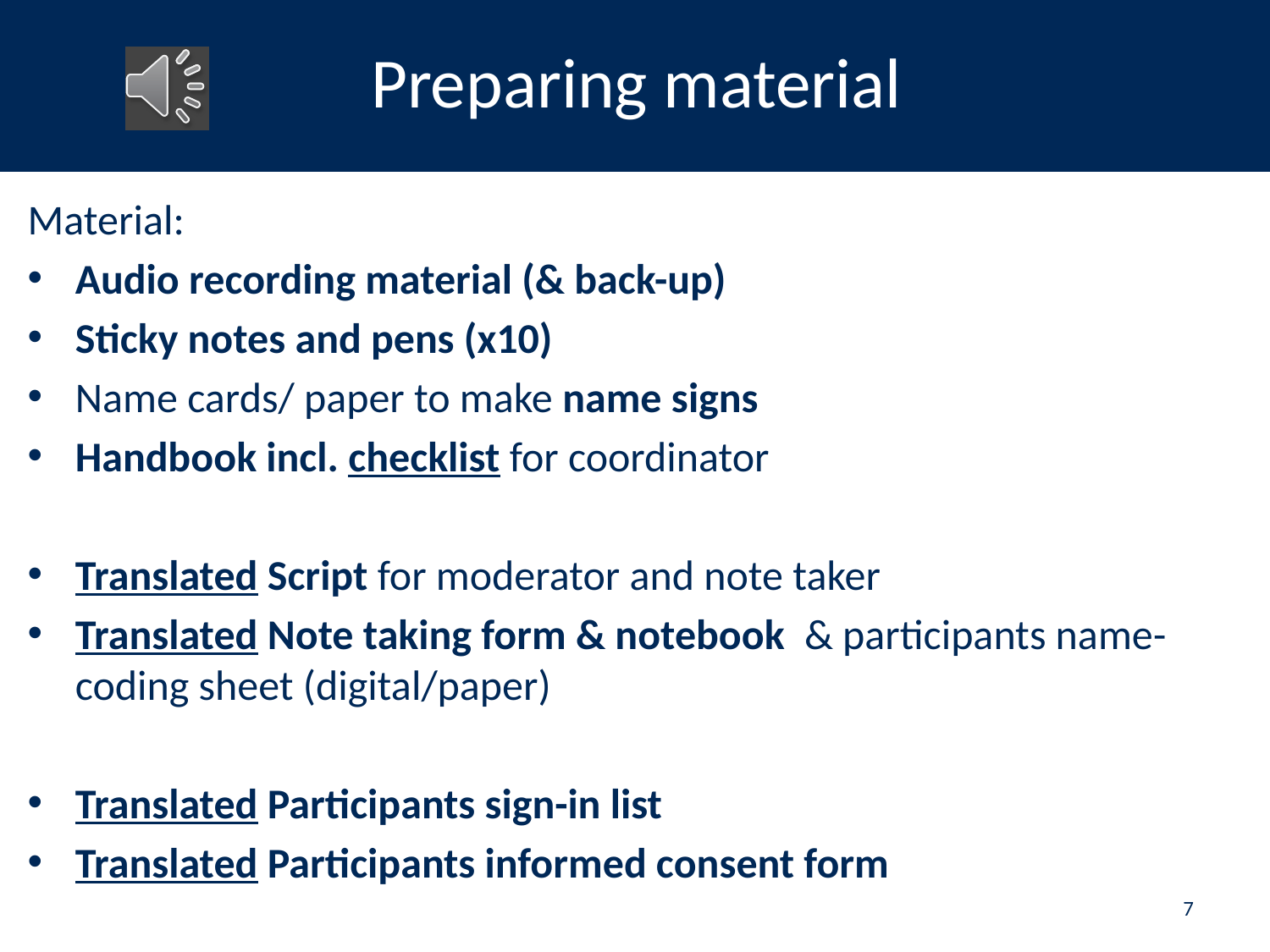

# Preparing material
Material:
Audio recording material (& back-up)
Sticky notes and pens (x10)
Name cards/ paper to make name signs
Handbook incl. checklist for coordinator
Translated Script for moderator and note taker
Translated Note taking form & notebook & participants name-coding sheet (digital/paper)
Translated Participants sign-in list
Translated Participants informed consent form
7

## Slide 8
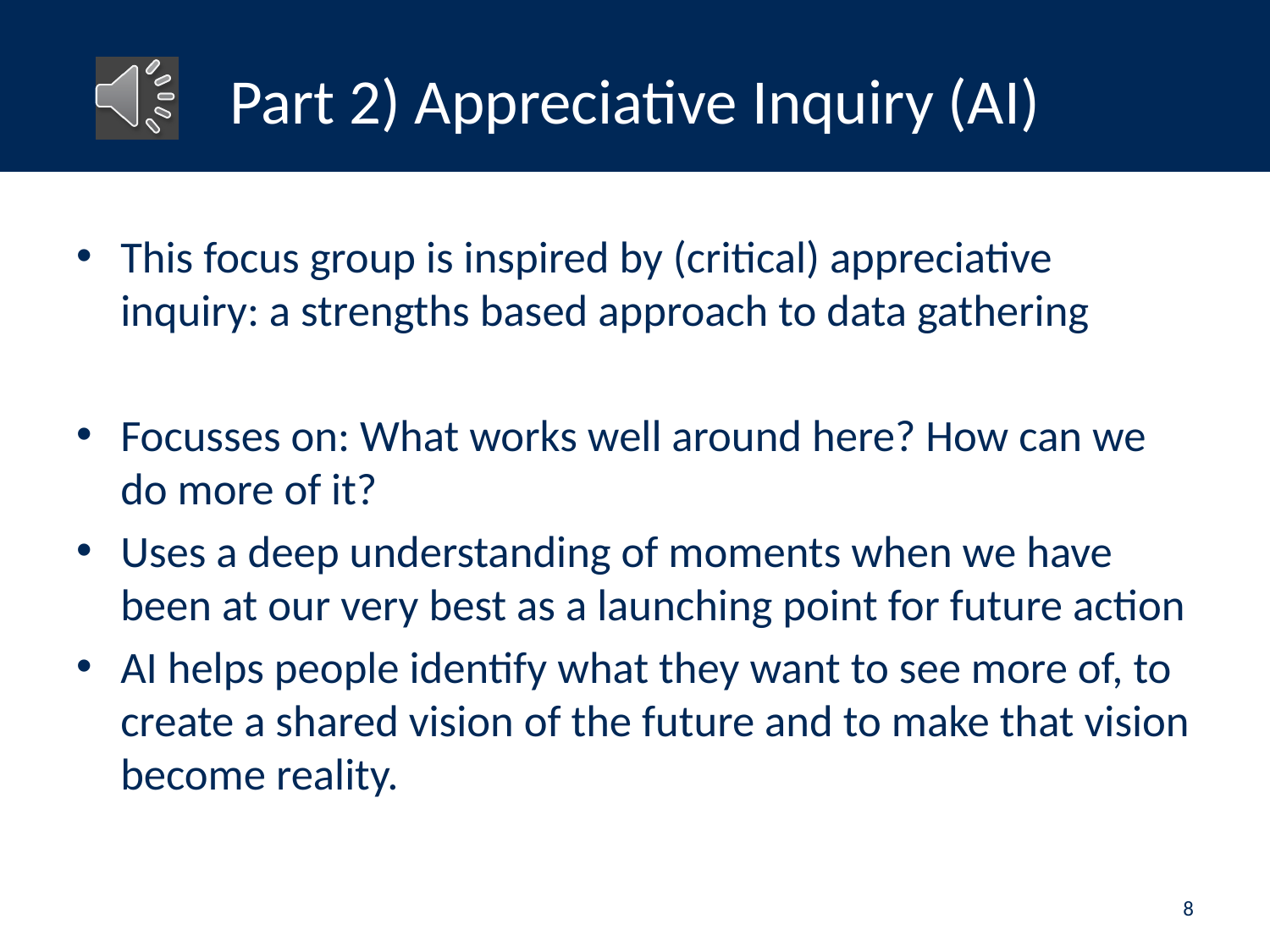

# Part 2) Appreciative Inquiry (AI)
This focus group is inspired by (critical) appreciative inquiry: a strengths based approach to data gathering
Focusses on: What works well around here? How can we do more of it?
Uses a deep understanding of moments when we have been at our very best as a launching point for future action
AI helps people identify what they want to see more of, to create a shared vision of the future and to make that vision become reality.
8

## Slide 9
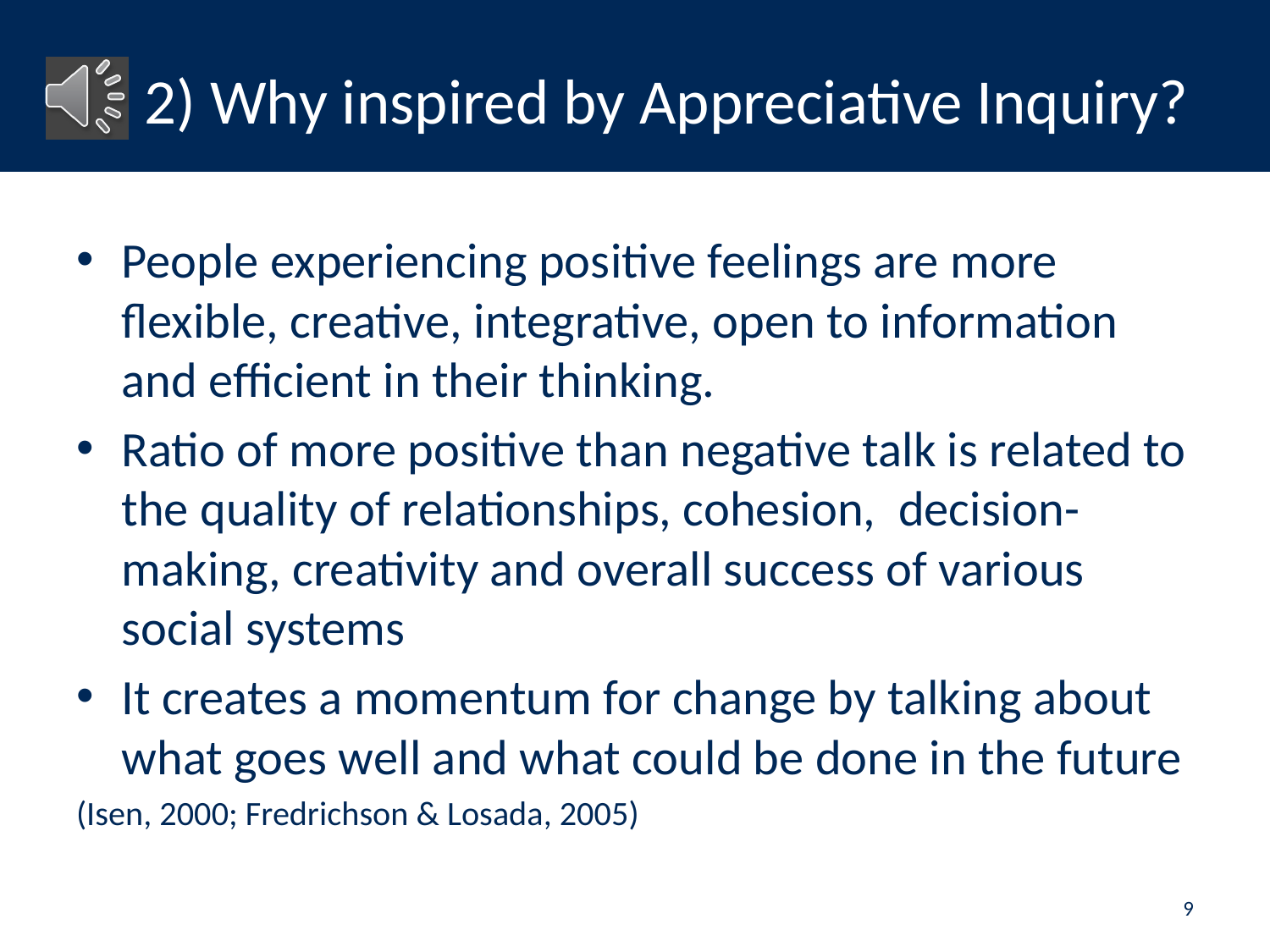

# 2) Why inspired by Appreciative Inquiry?
People experiencing positive feelings are more flexible, creative, integrative, open to information and efficient in their thinking.
Ratio of more positive than negative talk is related to the quality of relationships, cohesion, decision-making, creativity and overall success of various social systems
It creates a momentum for change by talking about what goes well and what could be done in the future
(Isen, 2000; Fredrichson & Losada, 2005)
9

## Slide 10
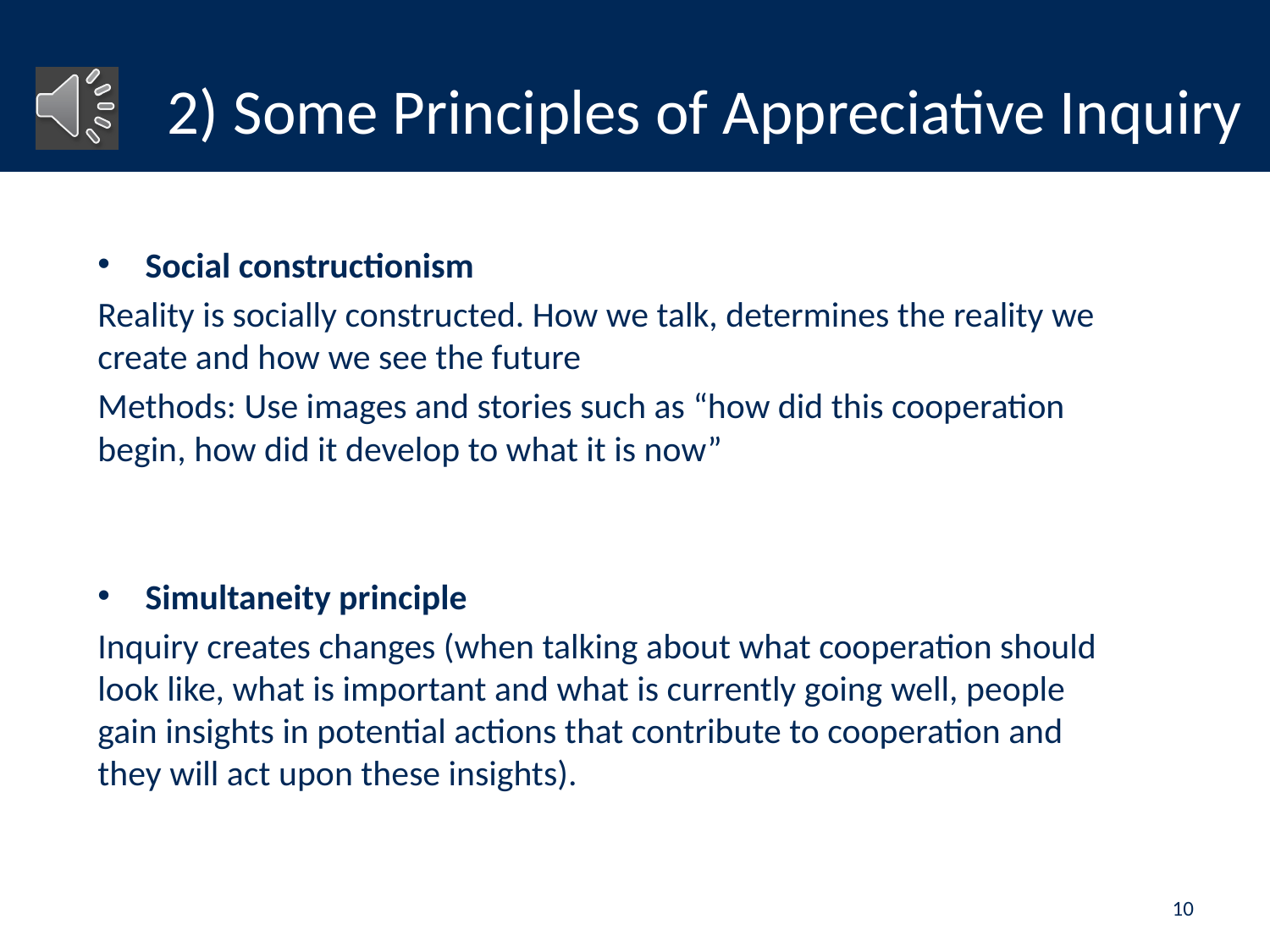

# 2) Some Principles of Appreciative Inquiry
Social constructionism
Reality is socially constructed. How we talk, determines the reality we create and how we see the future
Methods: Use images and stories such as “how did this cooperation begin, how did it develop to what it is now”
Simultaneity principle
Inquiry creates changes (when talking about what cooperation should look like, what is important and what is currently going well, people gain insights in potential actions that contribute to cooperation and they will act upon these insights).
10

## Slide 11
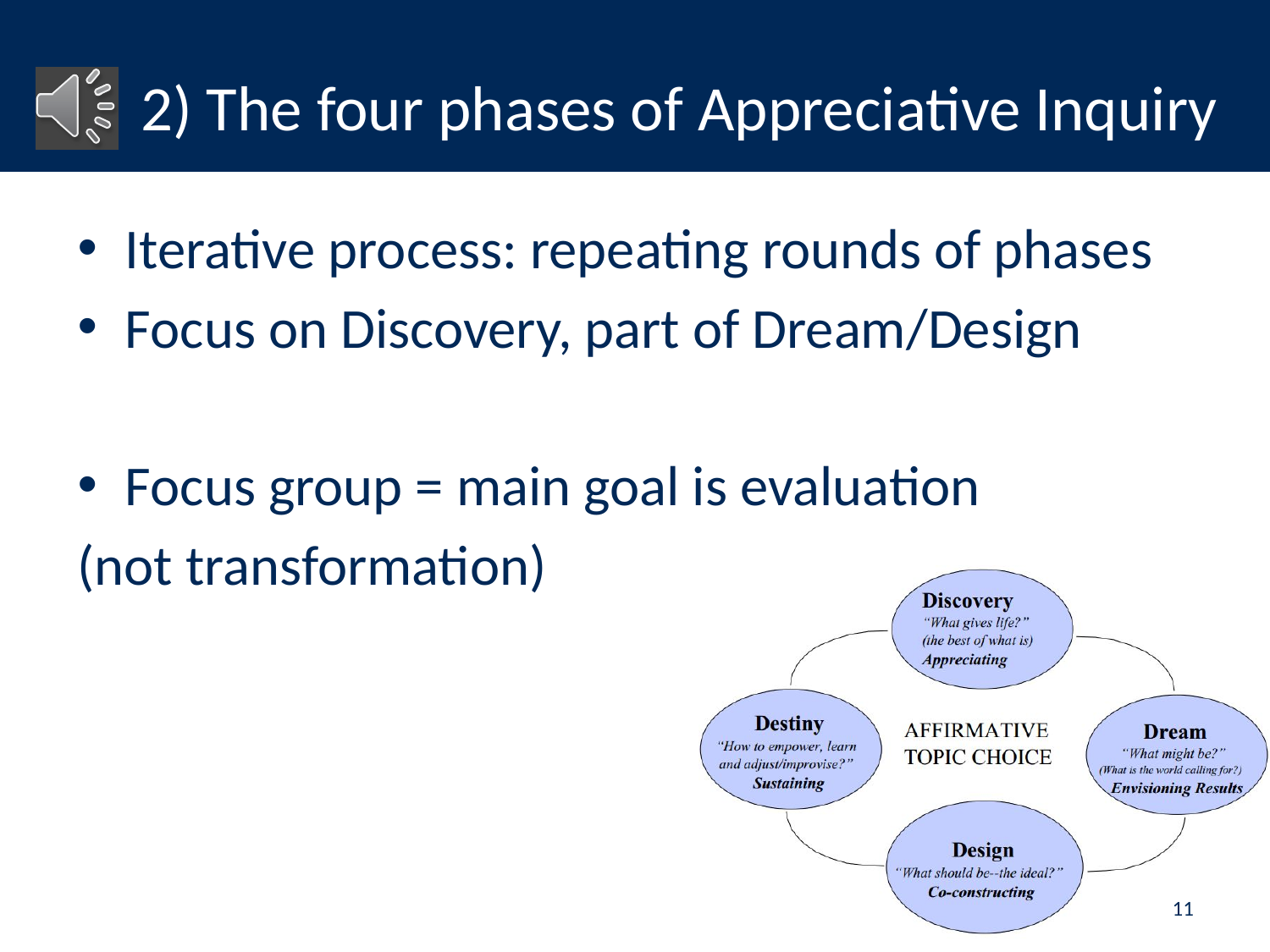

# 2) The four phases of Appreciative Inquiry
Iterative process: repeating rounds of phases
Focus on Discovery, part of Dream/Design
Focus group = main goal is evaluation
(not transformation)
11

## Slide 12
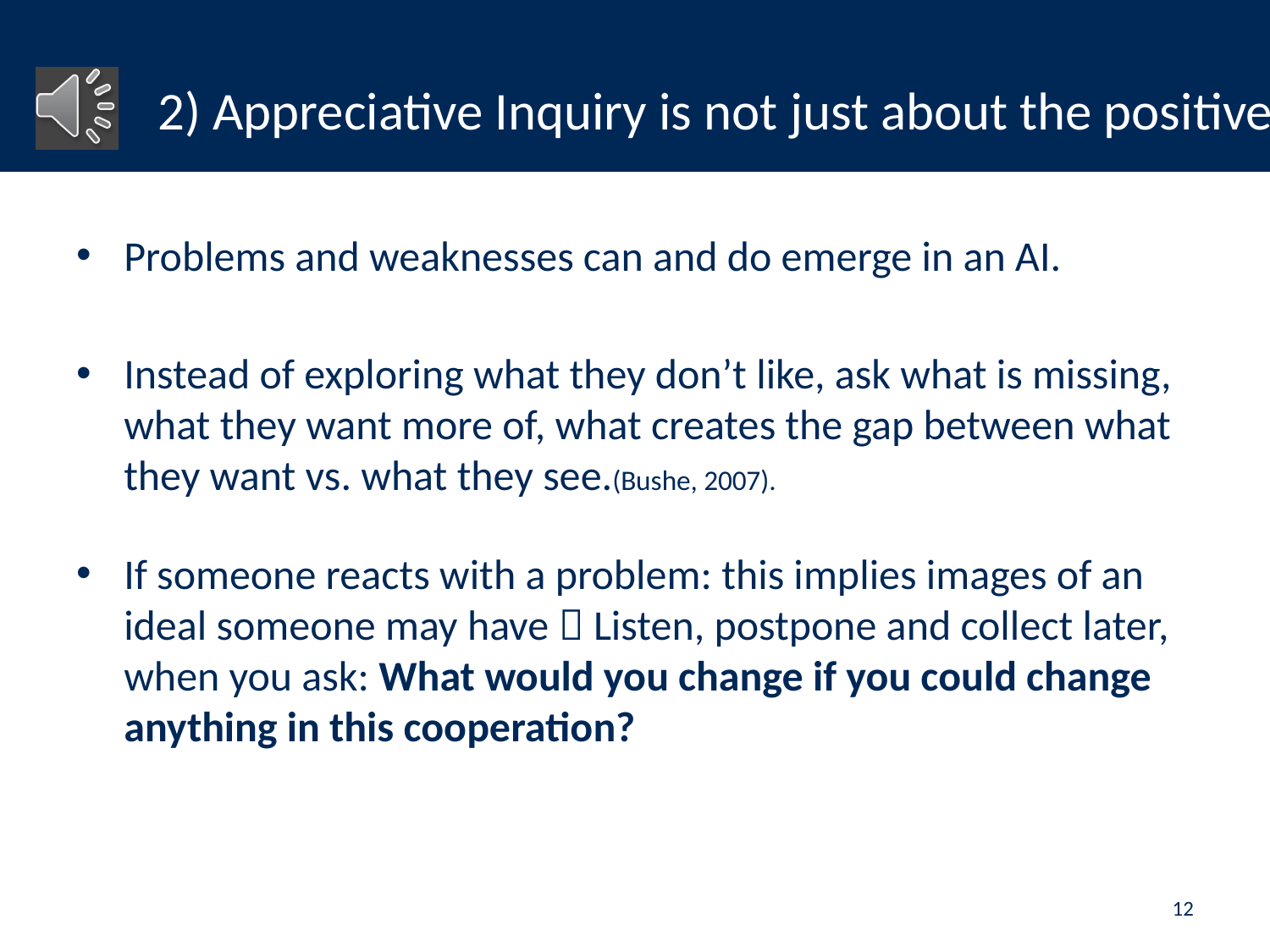

# 2) Appreciative Inquiry is not just about the positive
Problems and weaknesses can and do emerge in an AI.
Instead of exploring what they don’t like, ask what is missing, what they want more of, what creates the gap between what they want vs. what they see.(Bushe, 2007).
If someone reacts with a problem: this implies images of an ideal someone may have  Listen, postpone and collect later, when you ask: What would you change if you could change anything in this cooperation?
12

## Slide 13
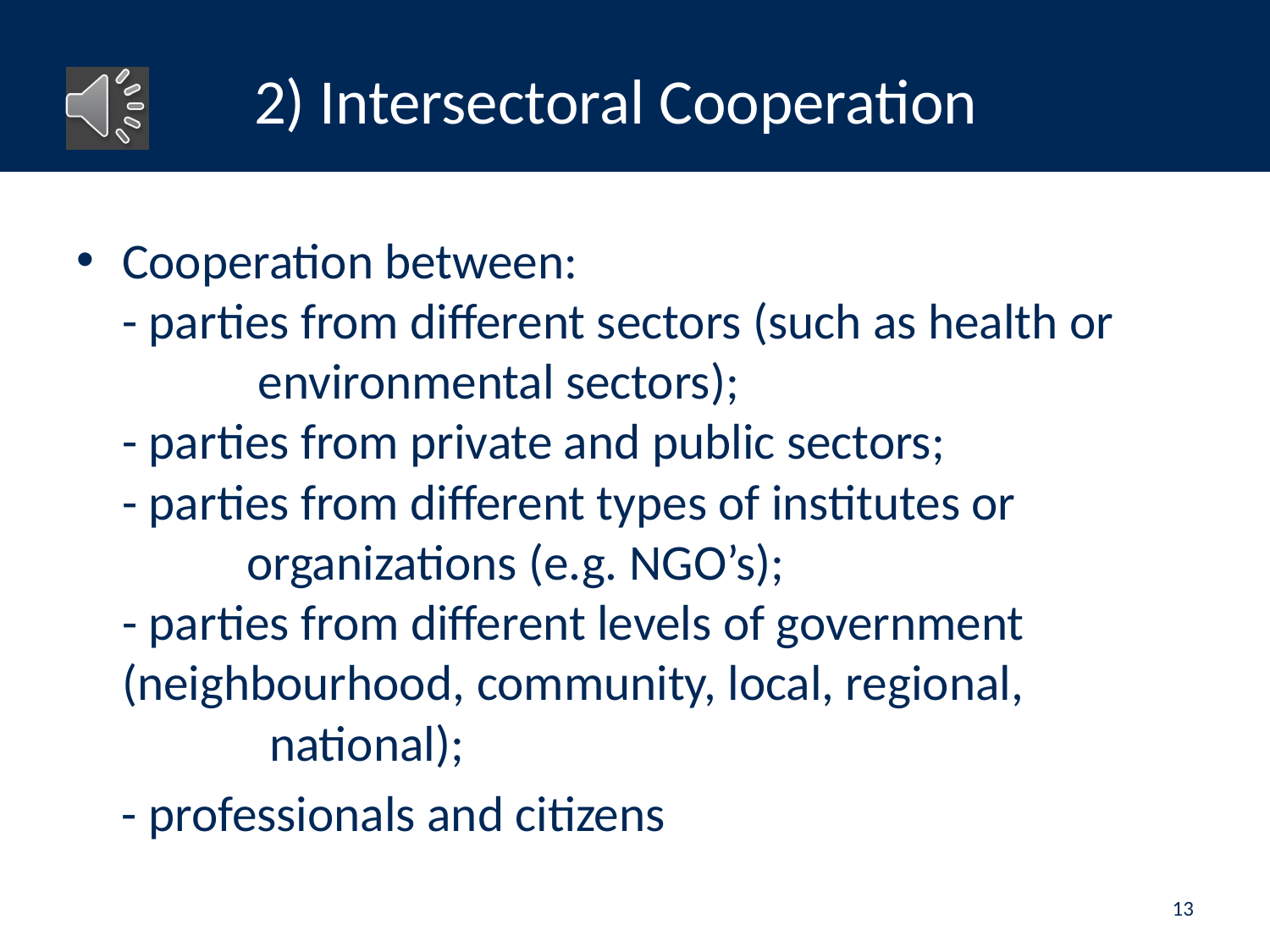

# 2) Intersectoral Cooperation
Cooperation between:
- parties from different sectors (such as health or 	 environmental sectors);
- parties from private and public sectors;
- parties from different types of institutes or 		 	 organizations (e.g. NGO’s);
- parties from different levels of government 		 (neighbourhood, community, local, regional, 	 	 national);
 - professionals and citizens
13

## Slide 14
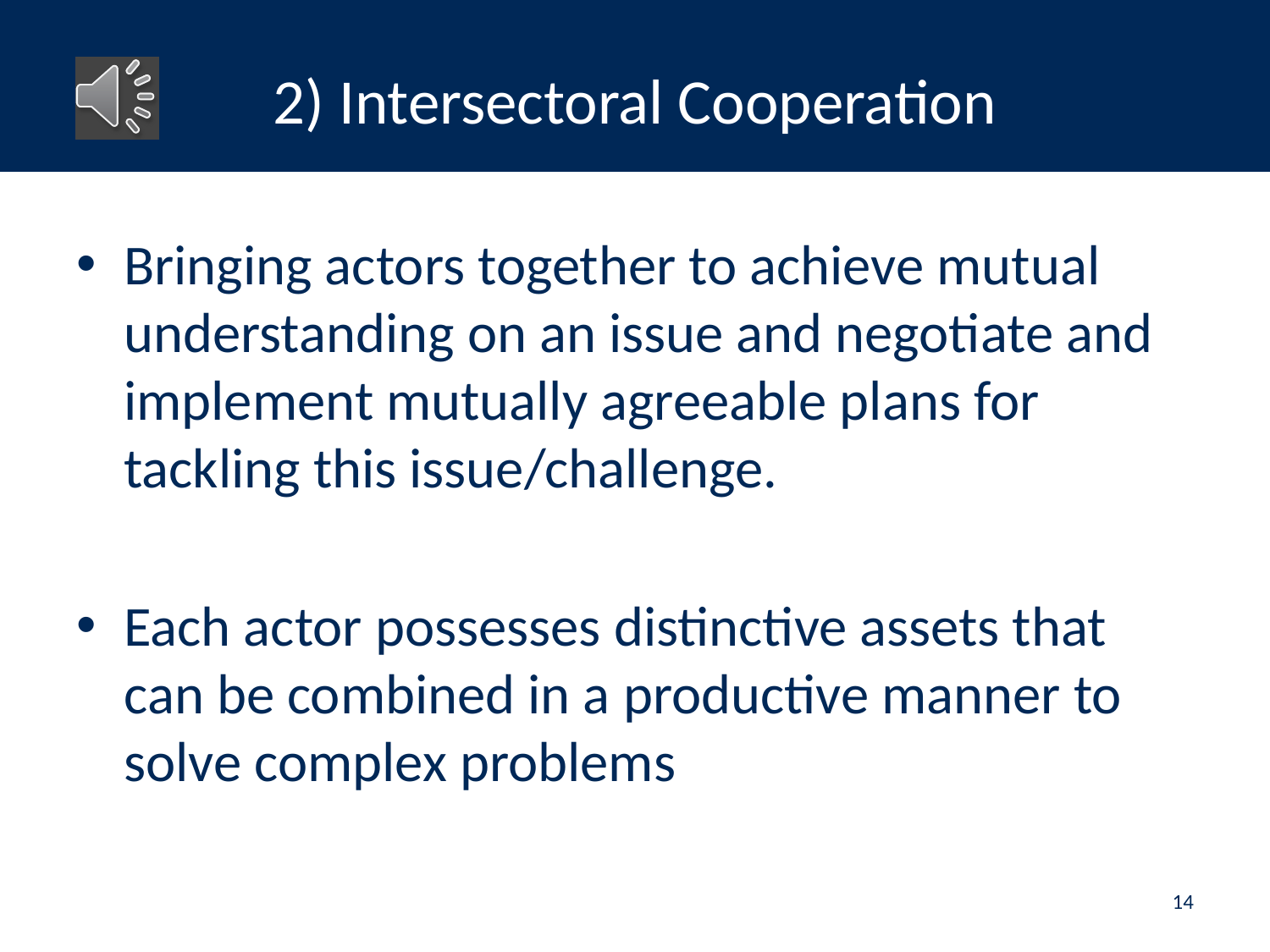

# 2) Intersectoral Cooperation
Bringing actors together to achieve mutual understanding on an issue and negotiate and implement mutually agreeable plans for tackling this issue/challenge.
Each actor possesses distinctive assets that can be combined in a productive manner to solve complex problems
14

## Slide 15
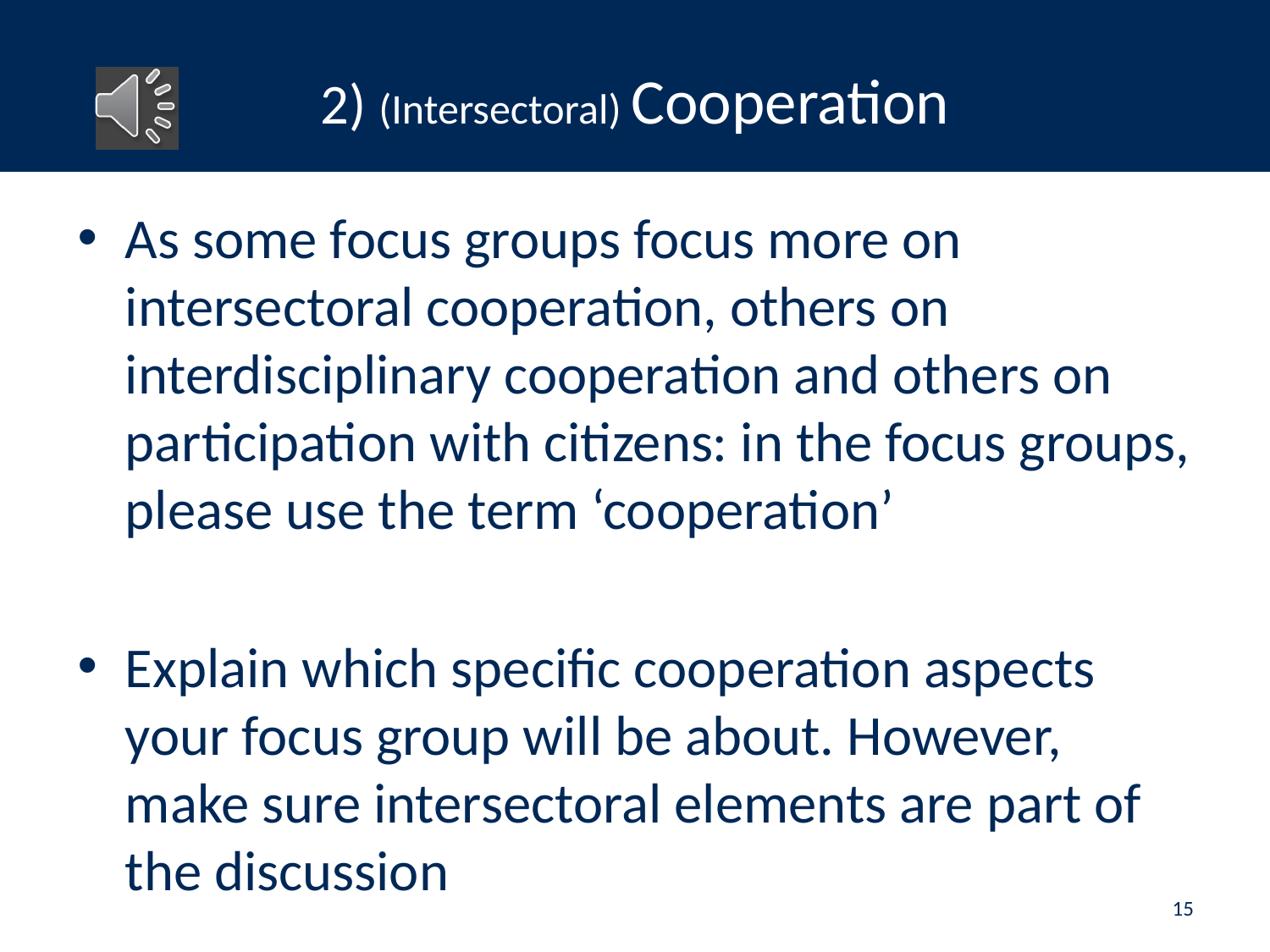

# 2) (Intersectoral) Cooperation
As some focus groups focus more on intersectoral cooperation, others on interdisciplinary cooperation and others on participation with citizens: in the focus groups, please use the term ‘cooperation’
Explain which specific cooperation aspects your focus group will be about. However, make sure intersectoral elements are part of the discussion
15

## Slide 16
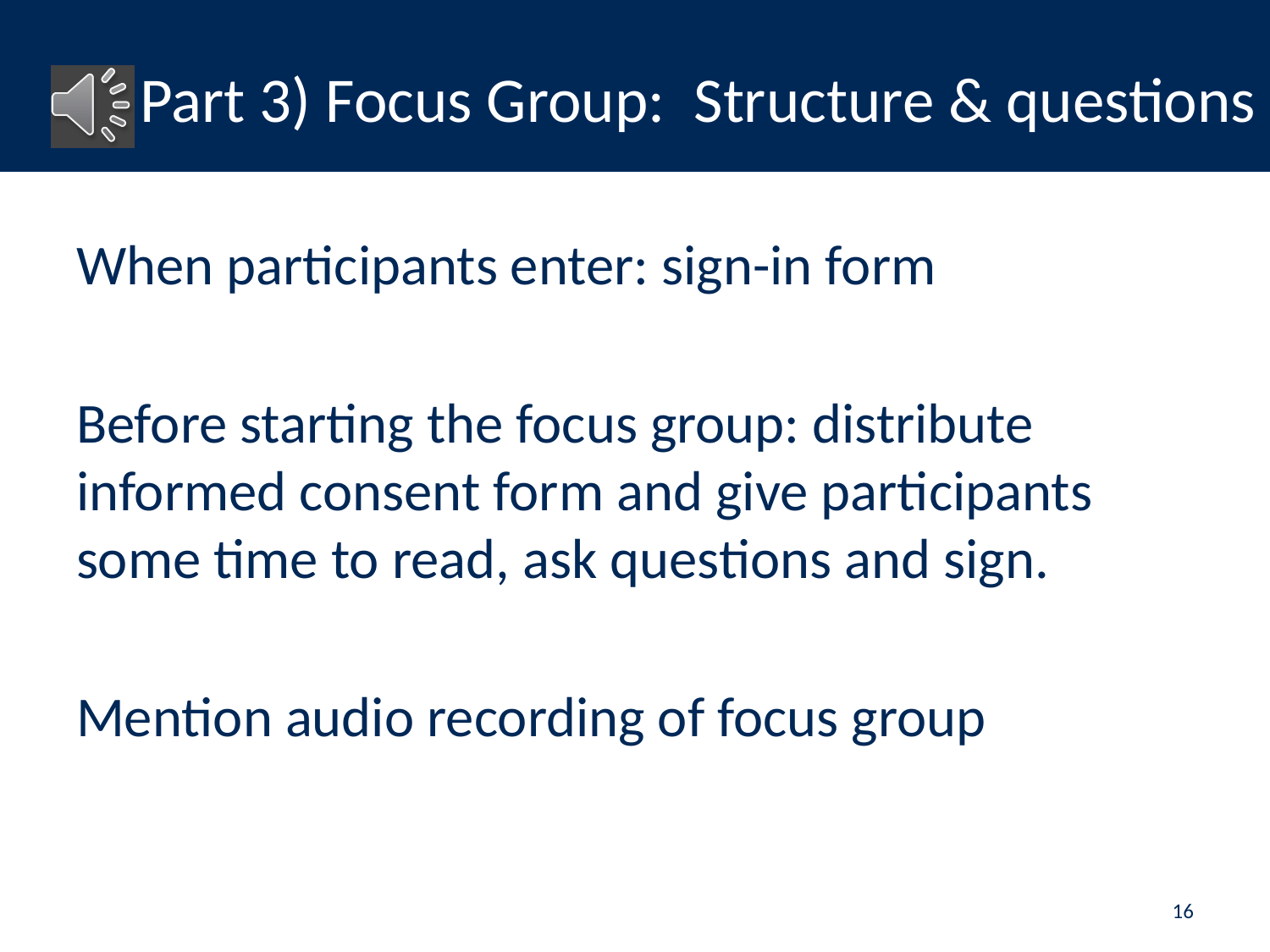

# Part 3) Focus Group: Structure & questions
When participants enter: sign-in form
Before starting the focus group: distribute informed consent form and give participants some time to read, ask questions and sign.
Mention audio recording of focus group
16

## Slide 17
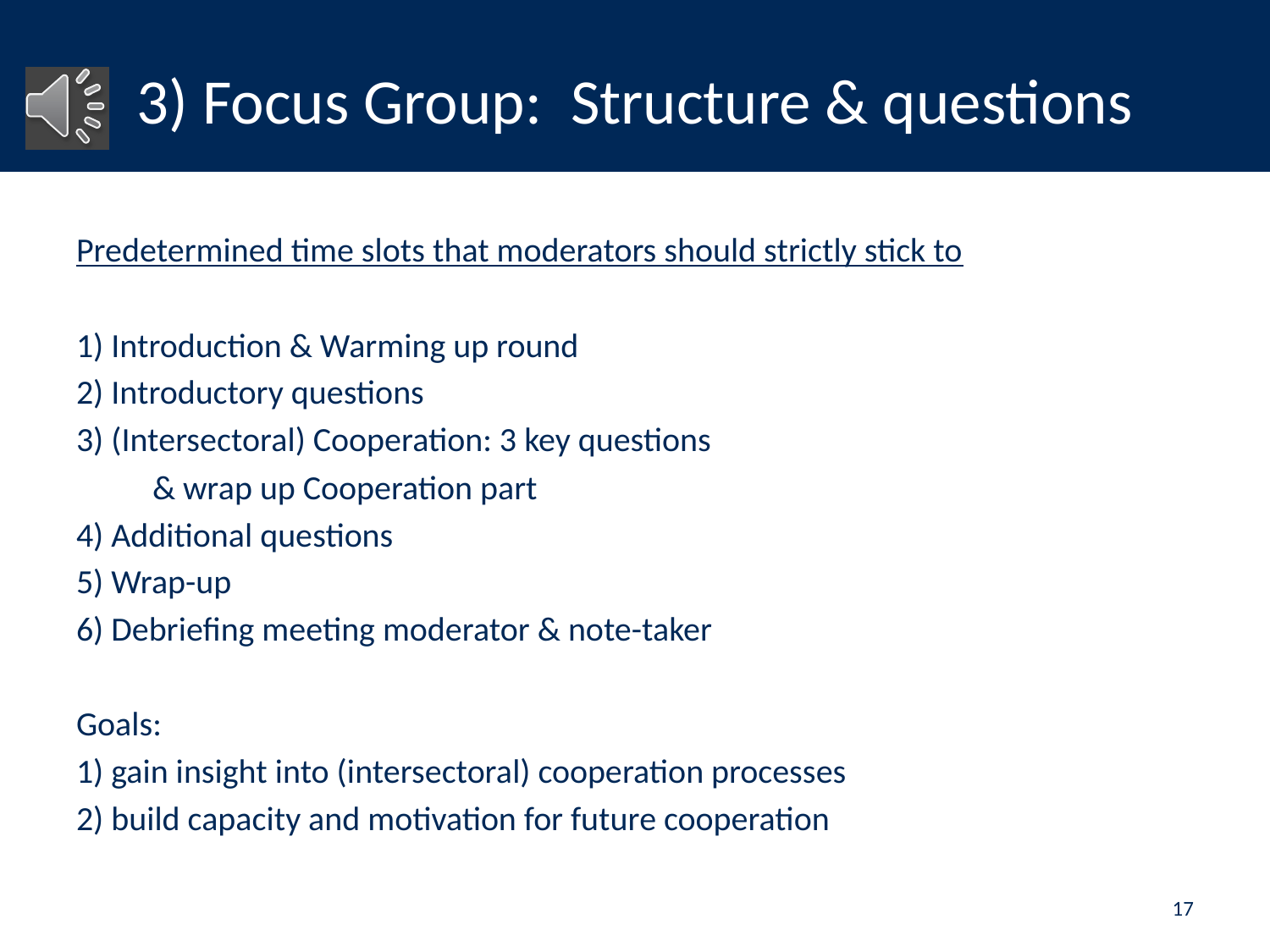

# 3) Focus Group: Structure & questions
Predetermined time slots that moderators should strictly stick to
1) Introduction & Warming up round
2) Introductory questions
3) (Intersectoral) Cooperation: 3 key questions
	& wrap up Cooperation part
4) Additional questions
5) Wrap-up
6) Debriefing meeting moderator & note-taker
Goals:
1) gain insight into (intersectoral) cooperation processes
2) build capacity and motivation for future cooperation
17

## Slide 18
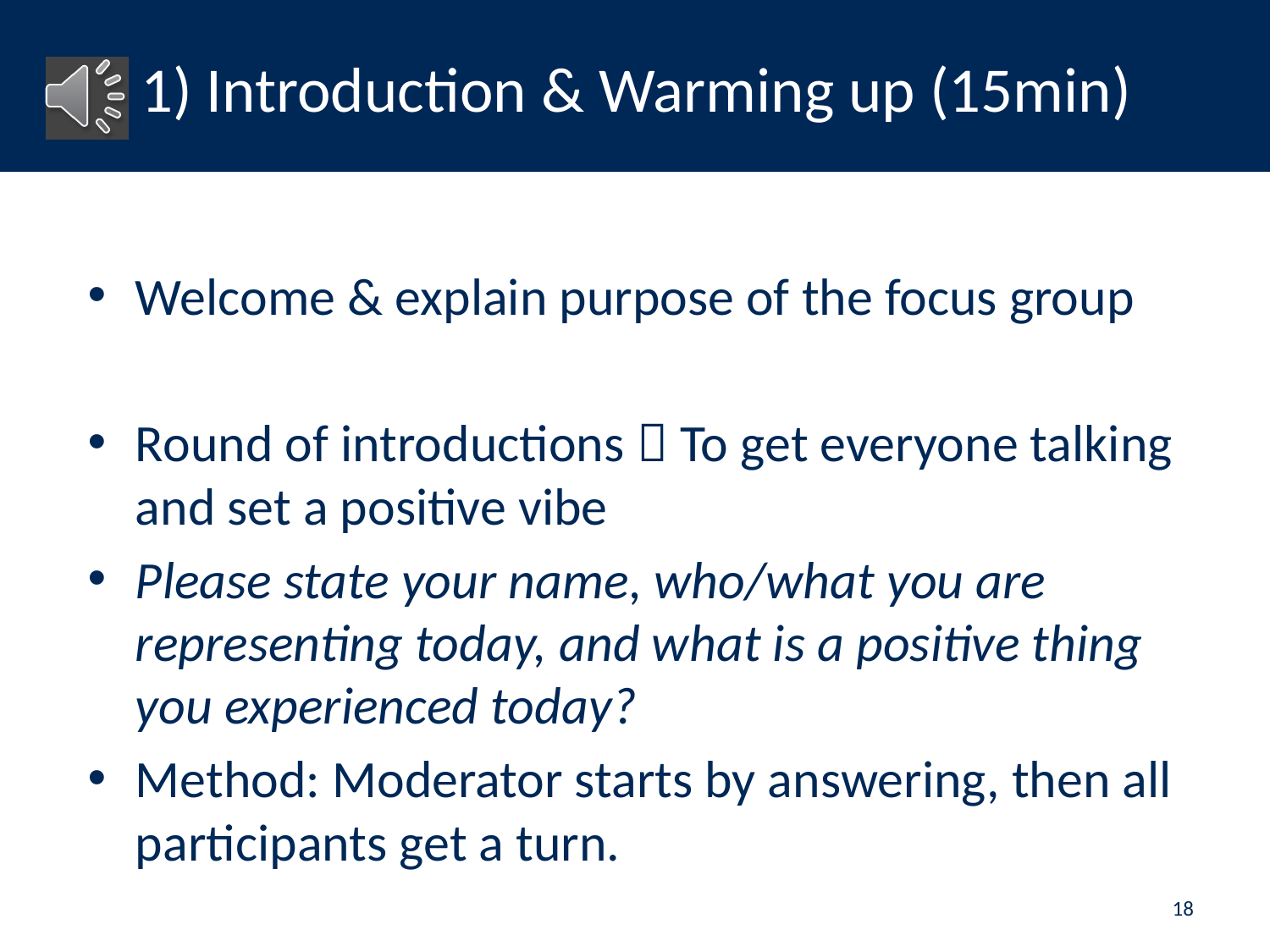

# 1) Introduction & Warming up (15min)
Welcome & explain purpose of the focus group
Round of introductions  To get everyone talking and set a positive vibe
Please state your name, who/what you are representing today, and what is a positive thing you experienced today?
Method: Moderator starts by answering, then all participants get a turn.
18

## Slide 19
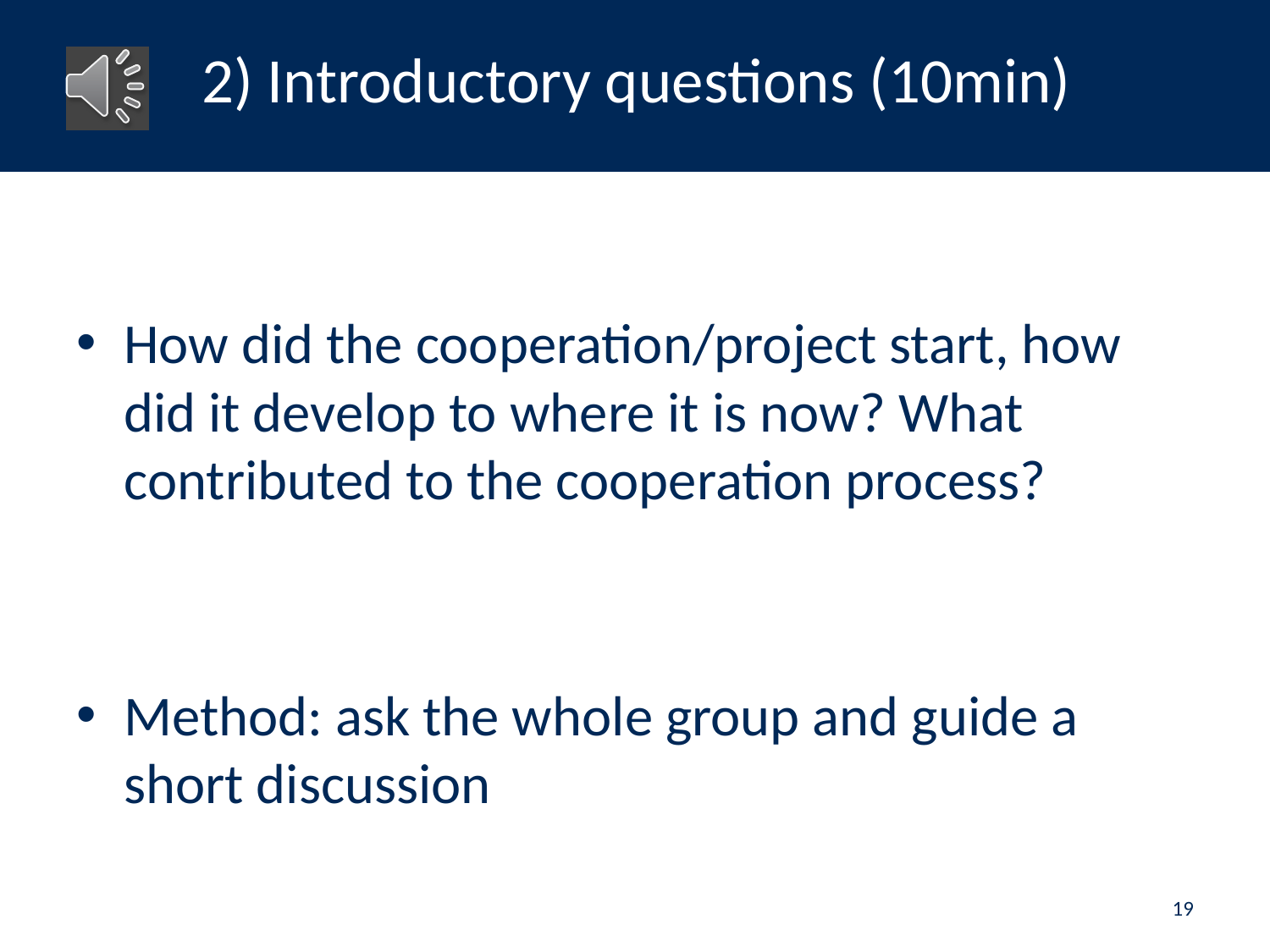

# 2) Introductory questions (10min)
How did the cooperation/project start, how did it develop to where it is now? What contributed to the cooperation process?
Method: ask the whole group and guide a short discussion
19

## Slide 20
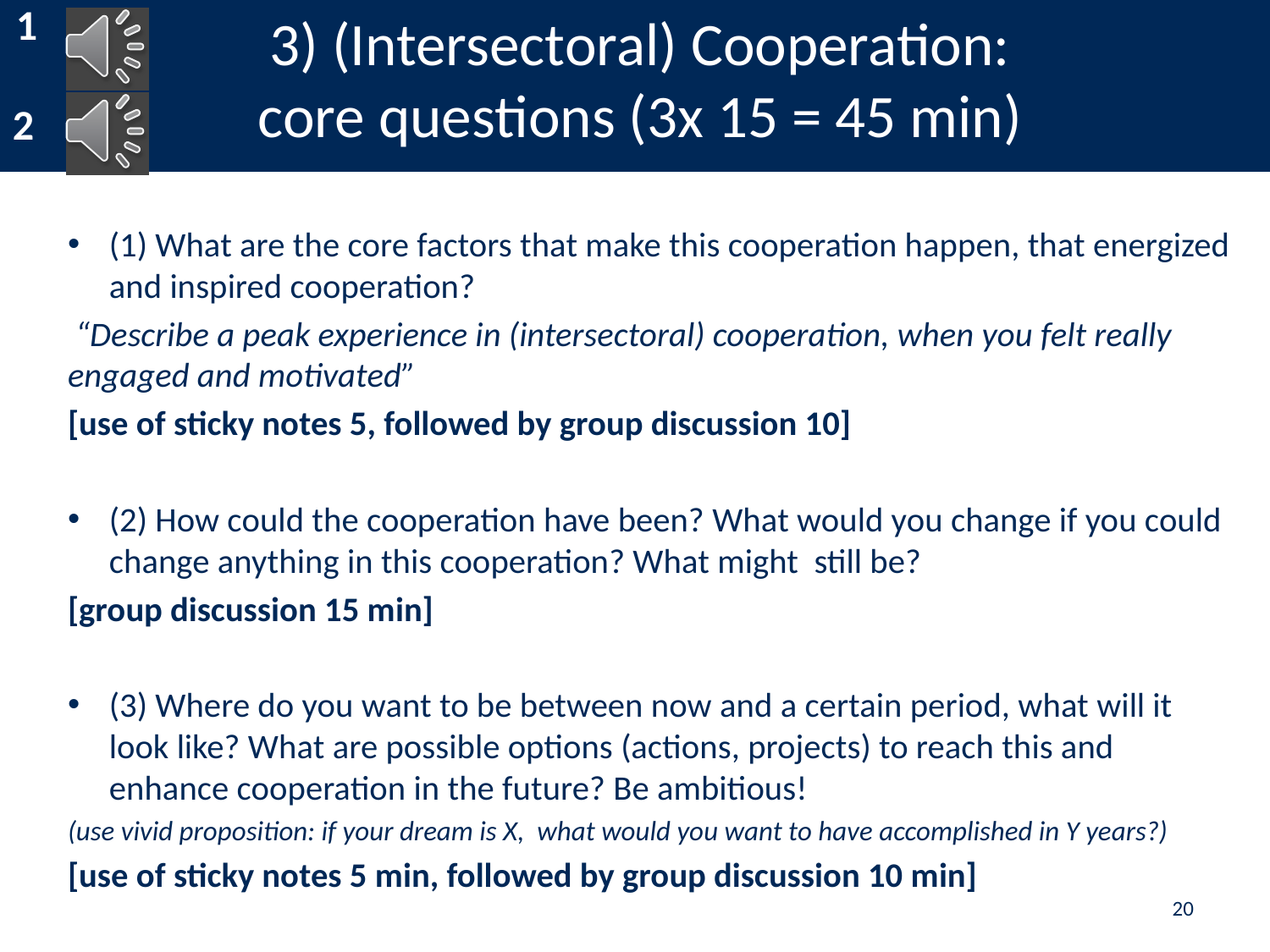

1
# 3) (Intersectoral) Cooperation: core questions (3x 15 = 45 min)
2
(1) What are the core factors that make this cooperation happen, that energized and inspired cooperation?
 “Describe a peak experience in (intersectoral) cooperation, when you felt really engaged and motivated”
[use of sticky notes 5, followed by group discussion 10]
(2) How could the cooperation have been? What would you change if you could change anything in this cooperation? What might still be?
[group discussion 15 min]
(3) Where do you want to be between now and a certain period, what will it look like? What are possible options (actions, projects) to reach this and enhance cooperation in the future? Be ambitious!
(use vivid proposition: if your dream is X, what would you want to have accomplished in Y years?)
[use of sticky notes 5 min, followed by group discussion 10 min]
20

## Slide 21
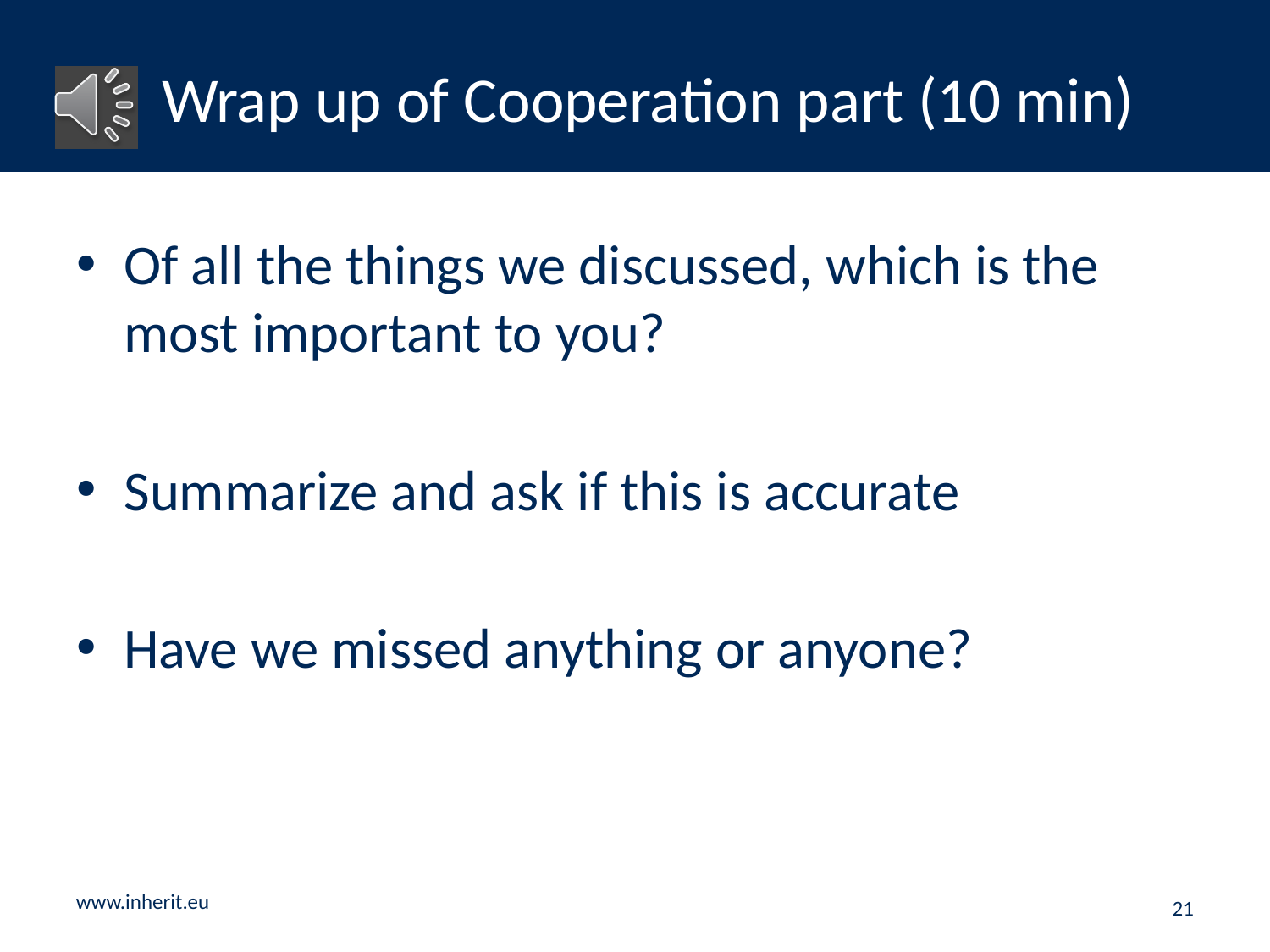

# Wrap up of Cooperation part (10 min)
Of all the things we discussed, which is the most important to you?
Summarize and ask if this is accurate
Have we missed anything or anyone?
www.inherit.eu
21

## Slide 22
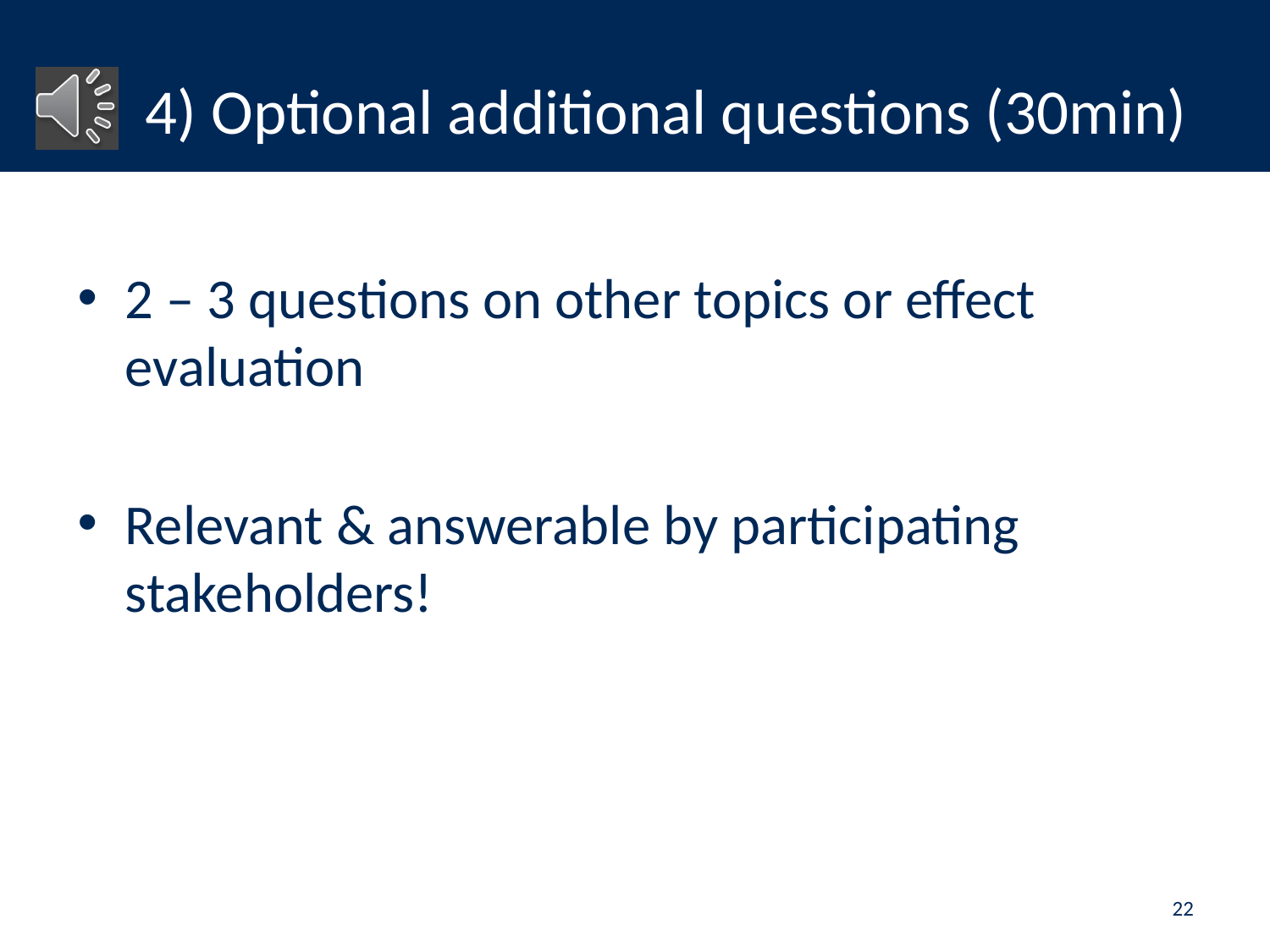

# 4) Optional additional questions (30min)
2 – 3 questions on other topics or effect evaluation
Relevant & answerable by participating stakeholders!
22

## Slide 23
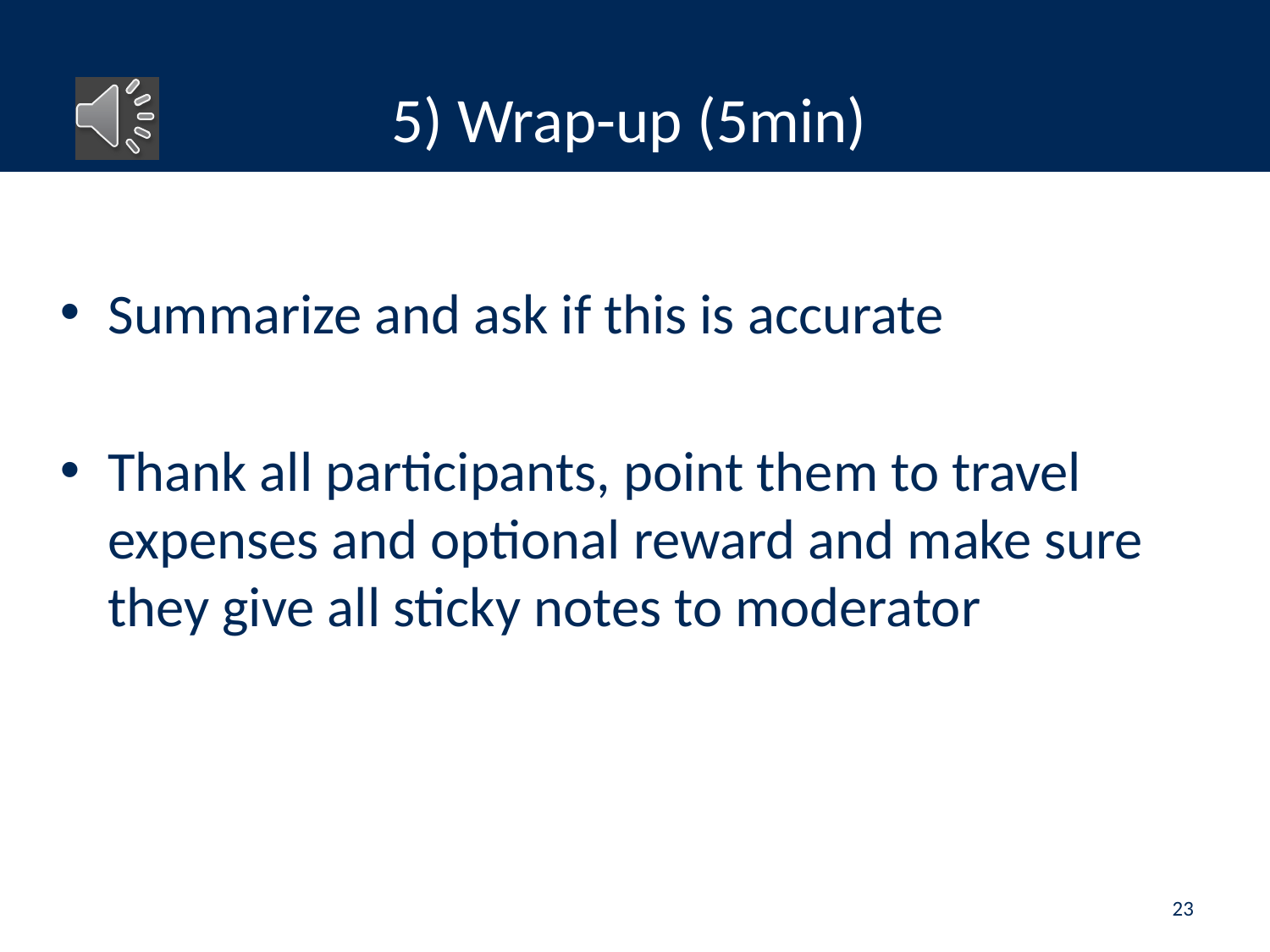

# 5) Wrap-up (5min)
Summarize and ask if this is accurate
Thank all participants, point them to travel expenses and optional reward and make sure they give all sticky notes to moderator
23

## Slide 24
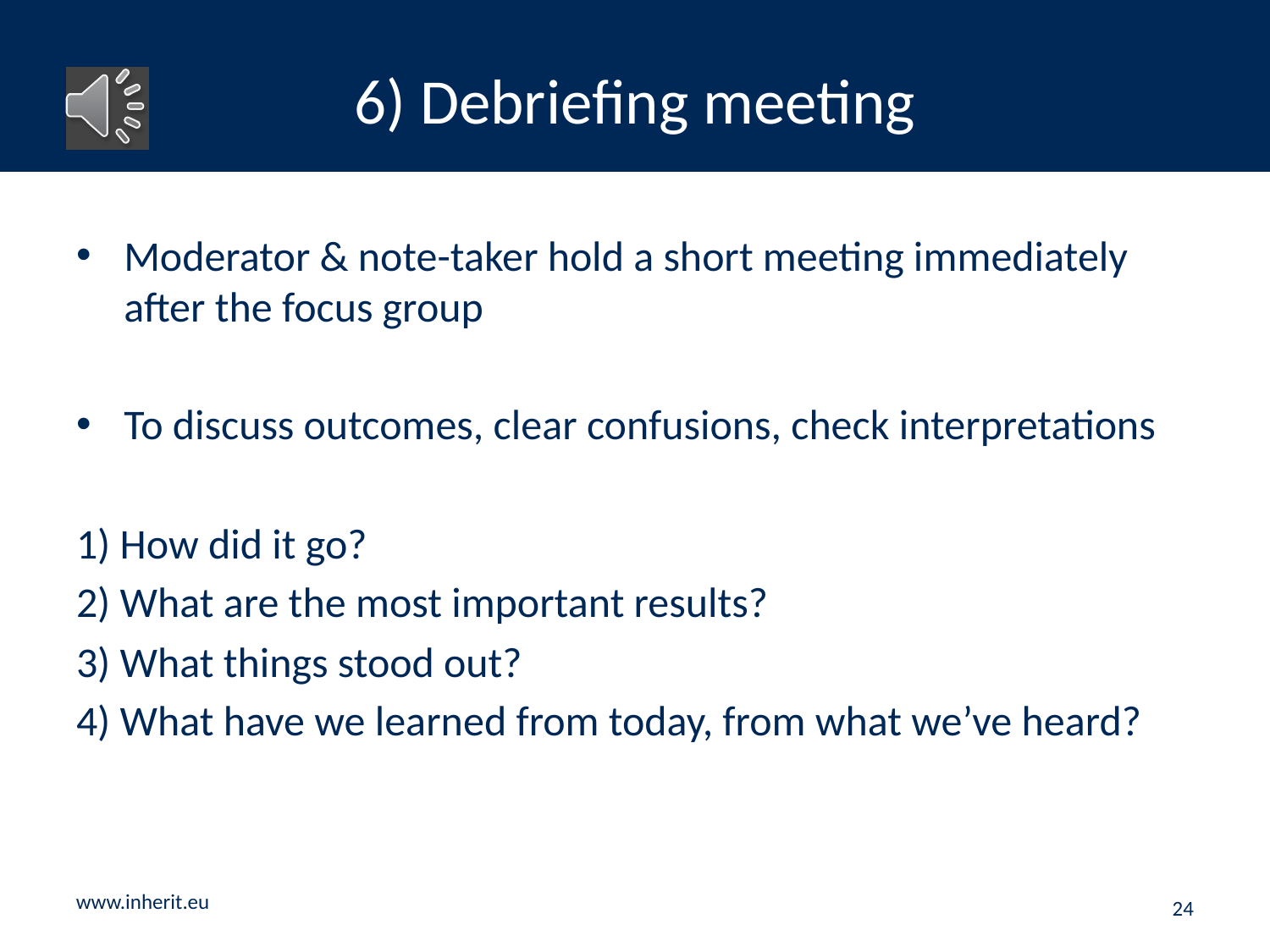

# 6) Debriefing meeting
Moderator & note-taker hold a short meeting immediately after the focus group
To discuss outcomes, clear confusions, check interpretations
1) How did it go?
2) What are the most important results?
3) What things stood out?
4) What have we learned from today, from what we’ve heard?
www.inherit.eu
24

## Slide 25
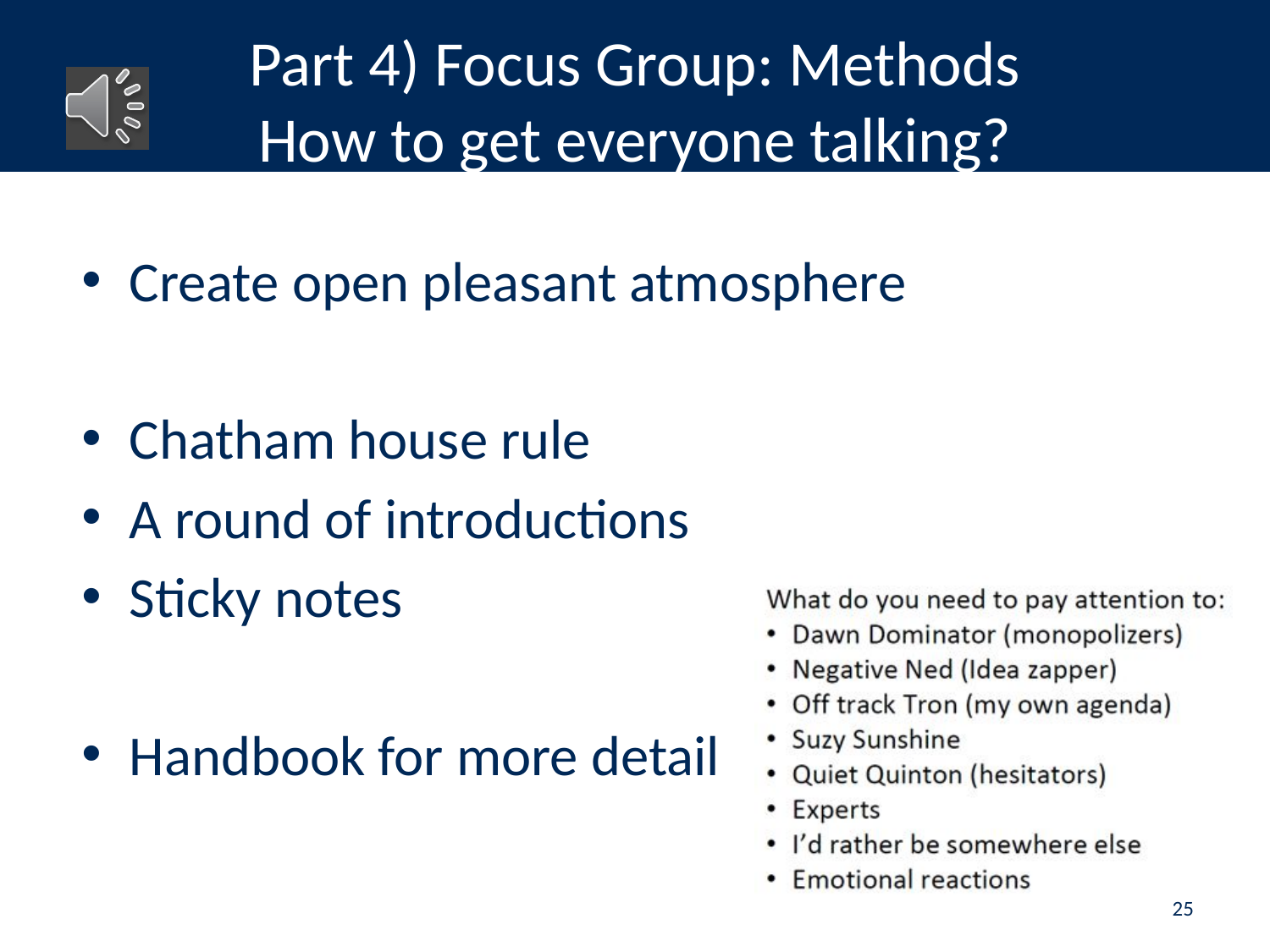

# Part 4) Focus Group: Methods How to get everyone talking?
Create open pleasant atmosphere
Chatham house rule
A round of introductions
Sticky notes
Handbook for more detail
25

## Slide 26
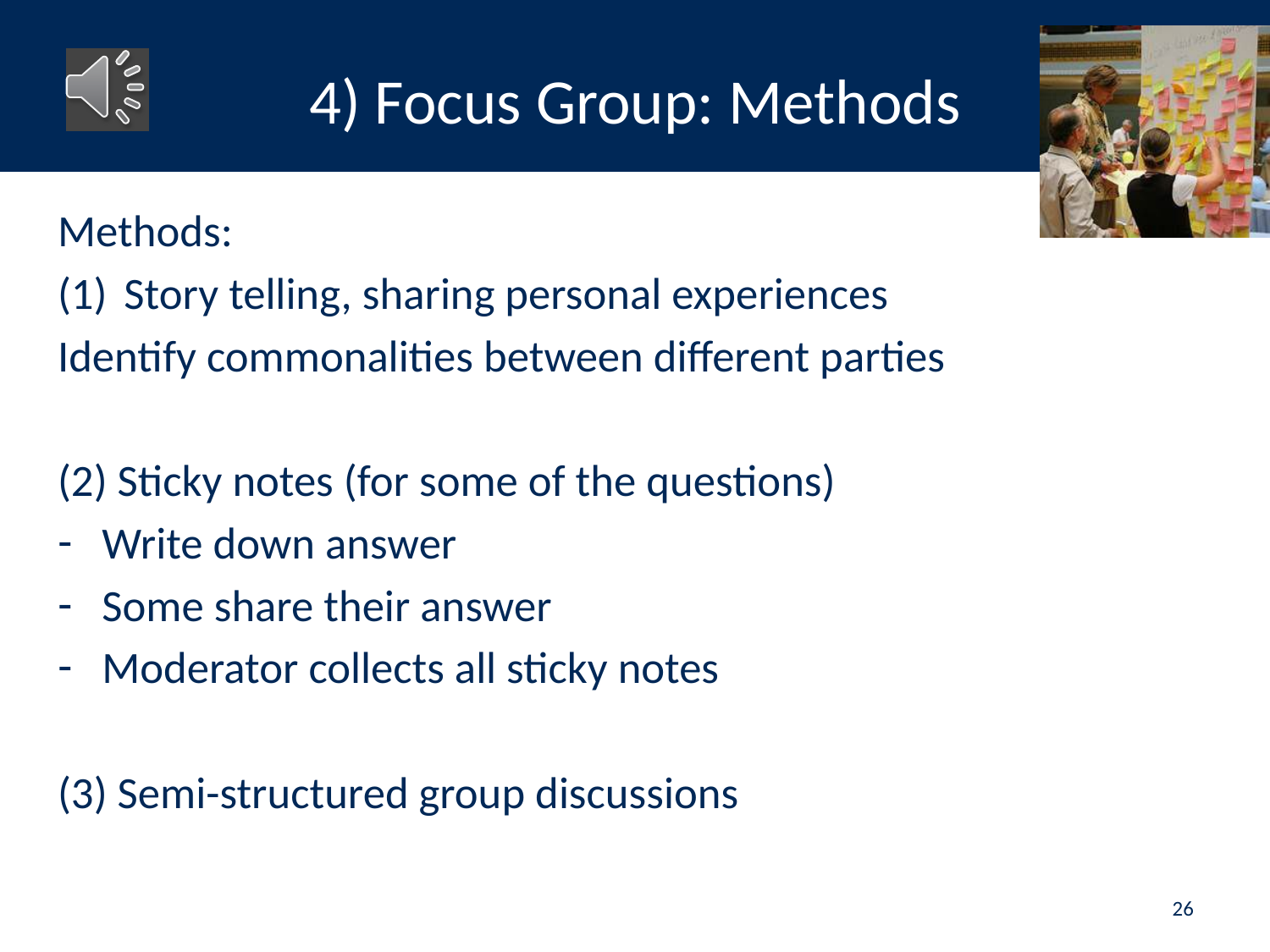

# 4) Focus Group: Methods
Methods:
Story telling, sharing personal experiences
Identify commonalities between different parties
(2) Sticky notes (for some of the questions)
Write down answer
Some share their answer
Moderator collects all sticky notes
(3) Semi-structured group discussions
26

## Slide 27
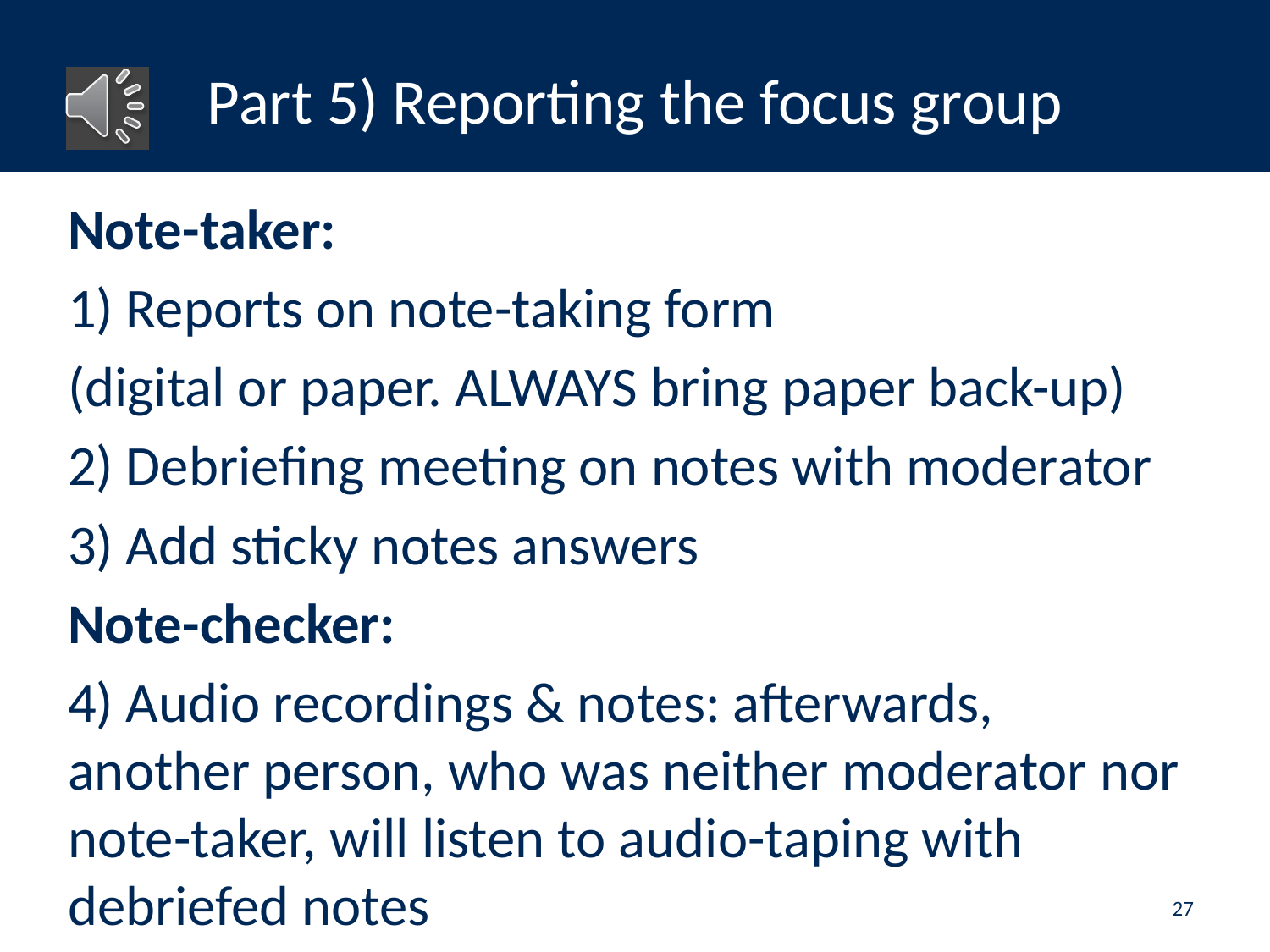

# Part 5) Reporting the focus group
Note-taker:
1) Reports on note-taking form
(digital or paper. ALWAYS bring paper back-up)
2) Debriefing meeting on notes with moderator
3) Add sticky notes answers
Note-checker:
4) Audio recordings & notes: afterwards, another person, who was neither moderator nor note-taker, will listen to audio-taping with debriefed notes
27

## Slide 28
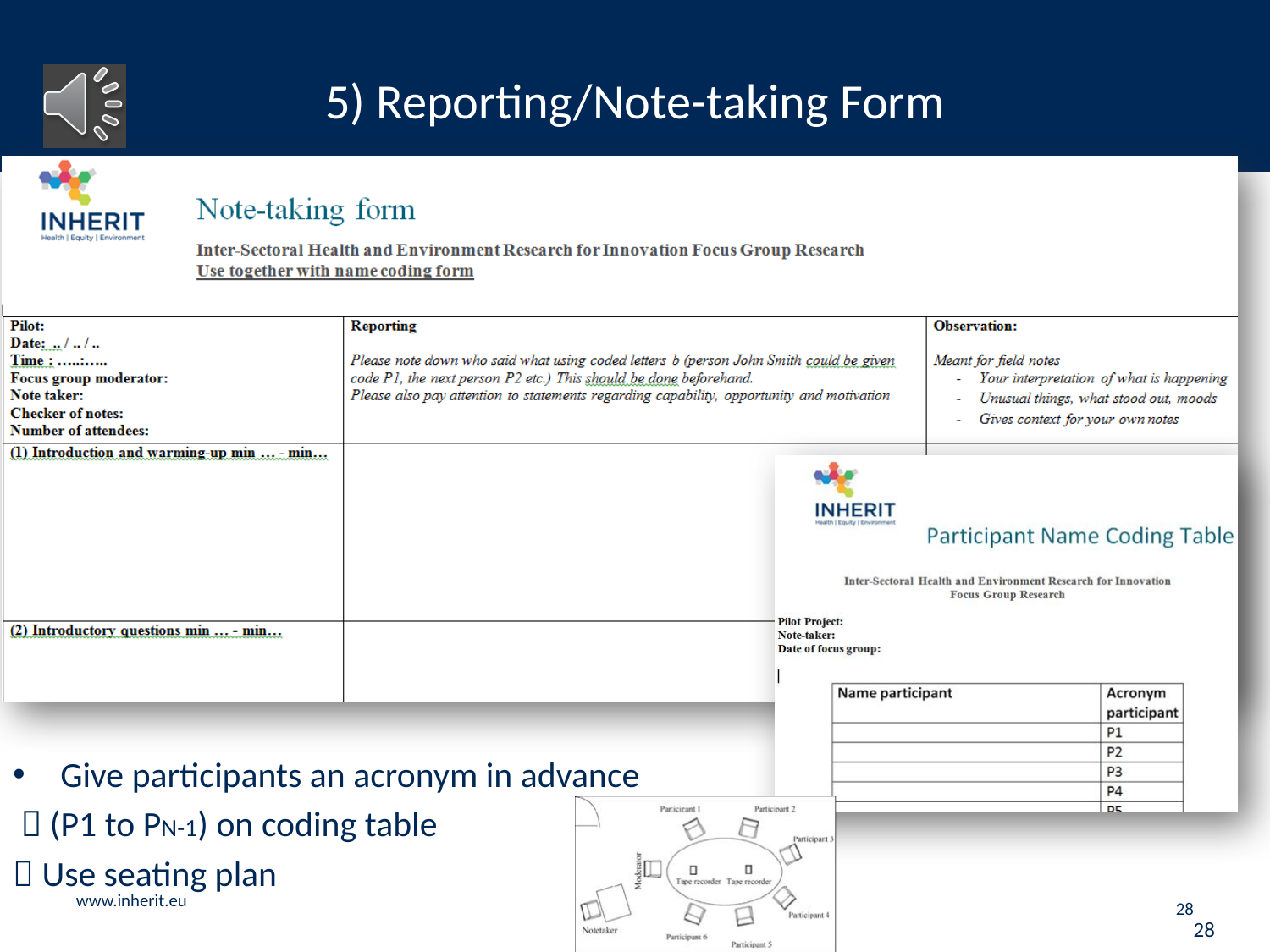

# 5) Reporting/Note-taking Form
Give participants an acronym in advance
  (P1 to PN-1) on coding table
 Use seating plan
www.inherit.eu
28
28

## Slide 29
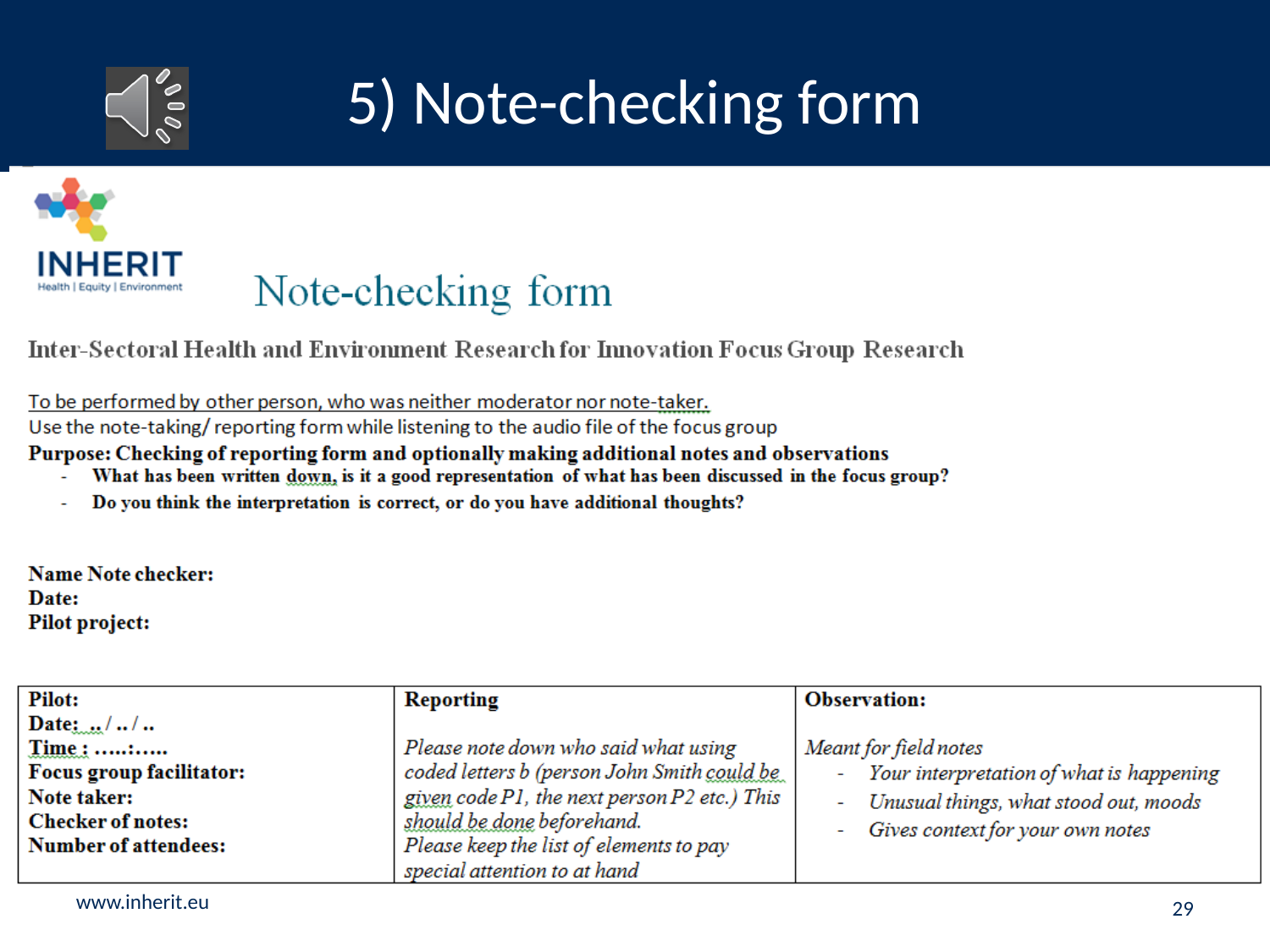

# 5) Note-checking form
www.inherit.eu
29

## Slide 30
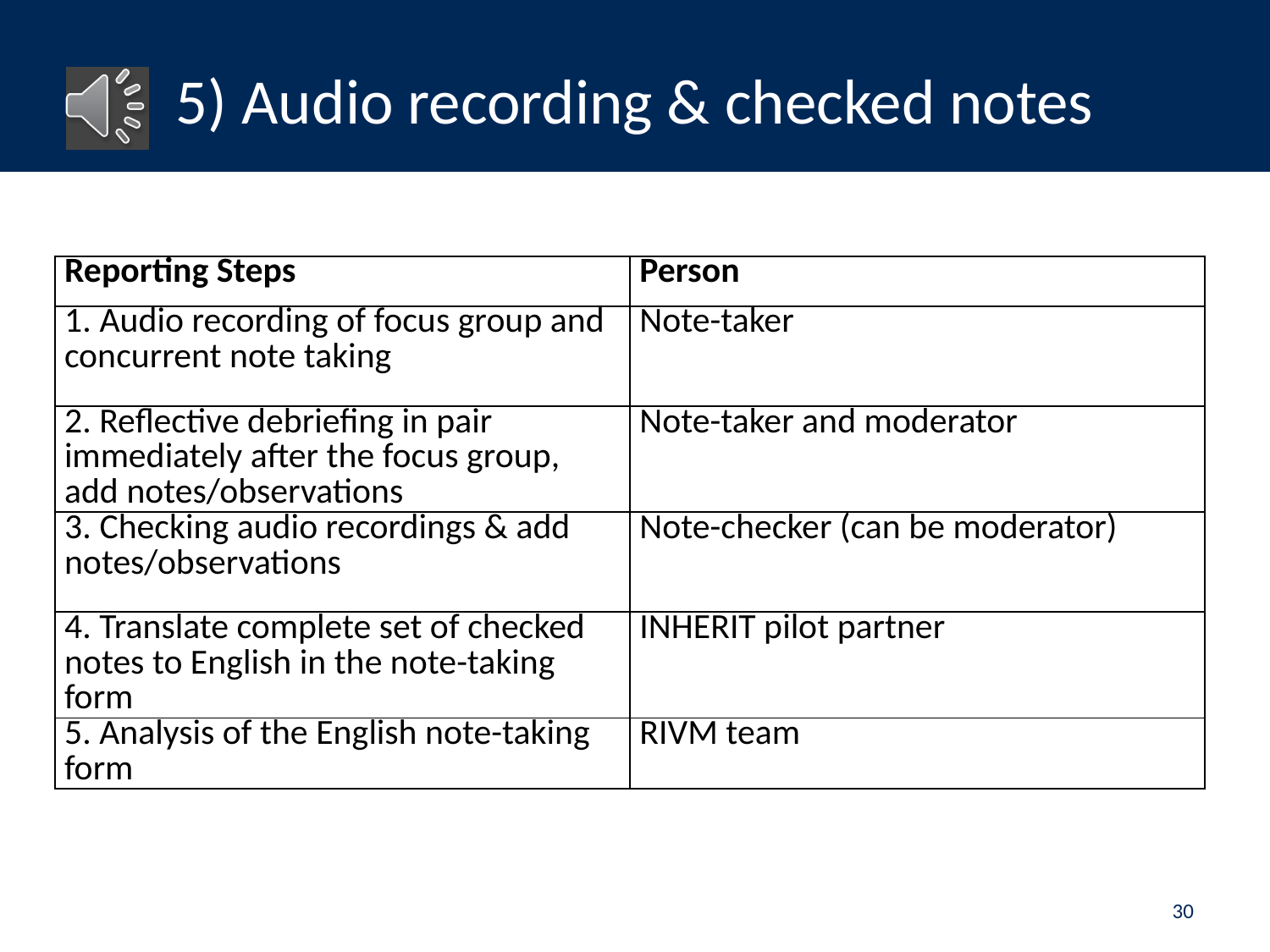

# 5) Audio recording & checked notes
| Reporting Steps | Person |
| --- | --- |
| 1. Audio recording of focus group and concurrent note taking | Note-taker |
| 2. Reflective debriefing in pair immediately after the focus group, add notes/observations | Note-taker and moderator |
| 3. Checking audio recordings & add notes/observations | Note-checker (can be moderator) |
| 4. Translate complete set of checked notes to English in the note-taking form | INHERIT pilot partner |
| 5. Analysis of the English note-taking form | RIVM team |
30

## Slide 31
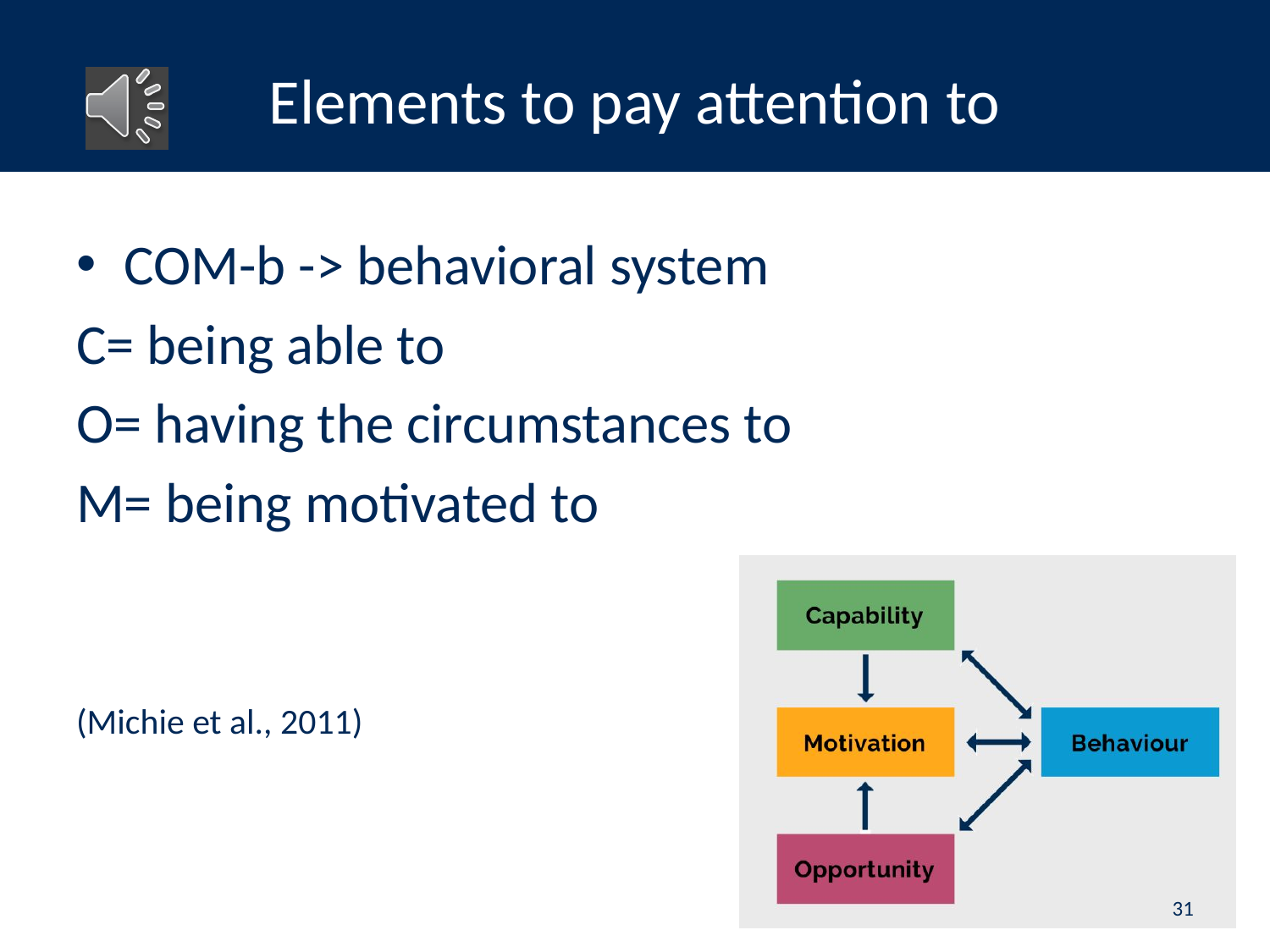

# Elements to pay attention to
COM-b -> behavioral system
C= being able to
O= having the circumstances to
M= being motivated to
(Michie et al., 2011)
31

## Slide 32
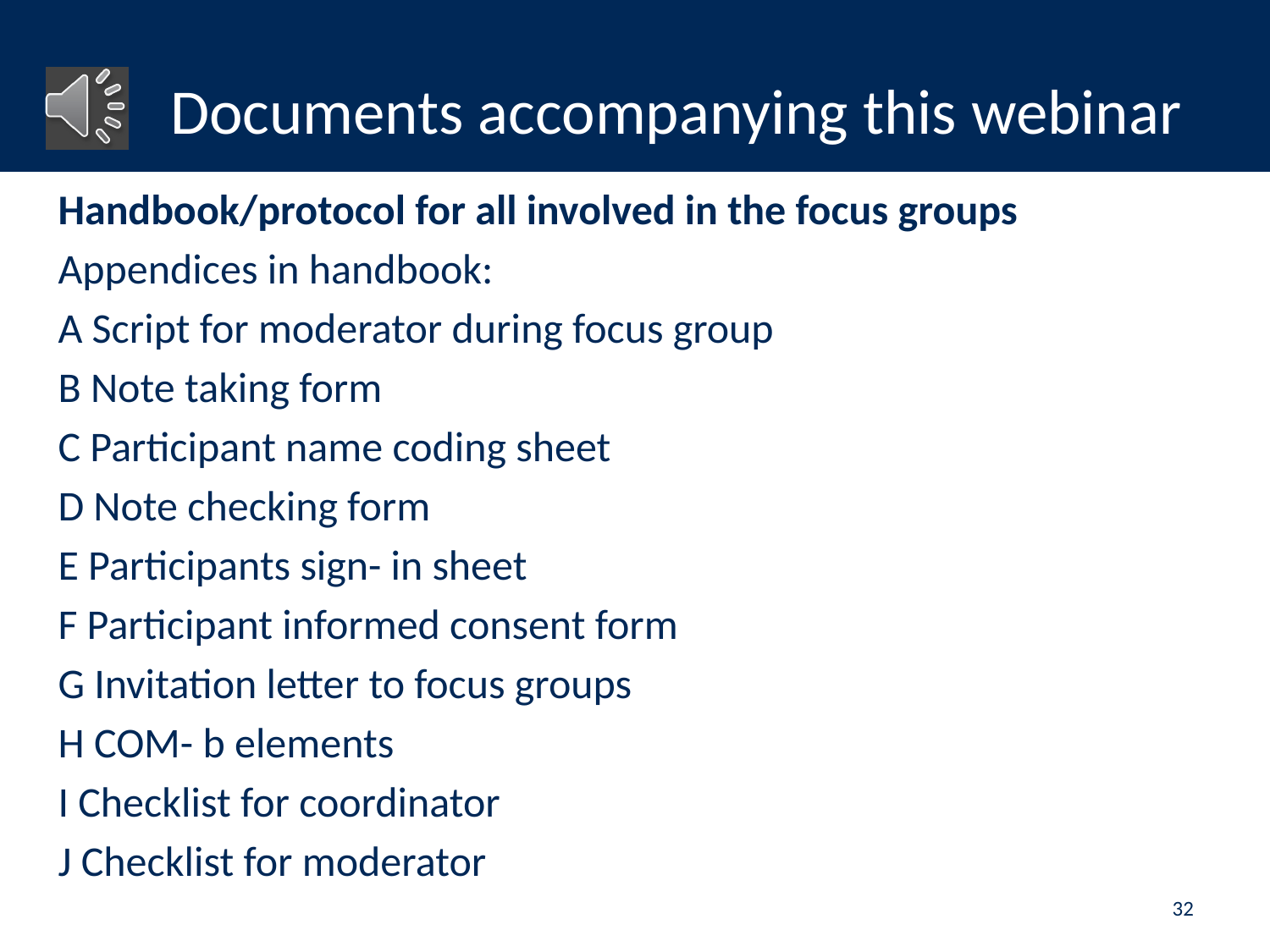

# Documents accompanying this webinar
Handbook/protocol for all involved in the focus groups
Appendices in handbook:
A Script for moderator during focus group
B Note taking form
C Participant name coding sheet
D Note checking form
E Participants sign- in sheet
F Participant informed consent form
G Invitation letter to focus groups
H COM- b elements
I Checklist for coordinator
J Checklist for moderator
32

## Slide 33
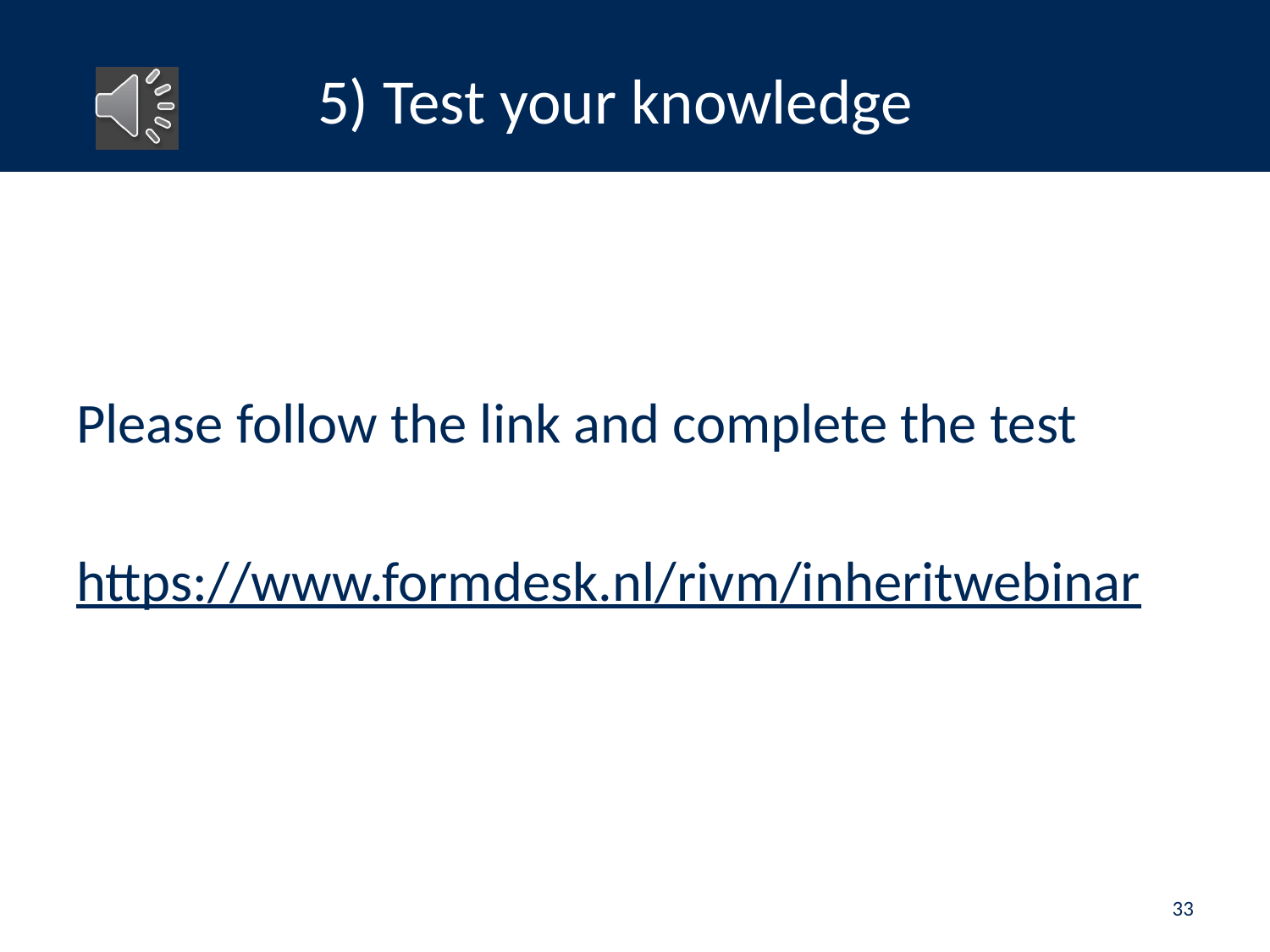

# 5) Test your knowledge
Please follow the link and complete the test
https://www.formdesk.nl/rivm/inheritwebinar
33

## Slide 34
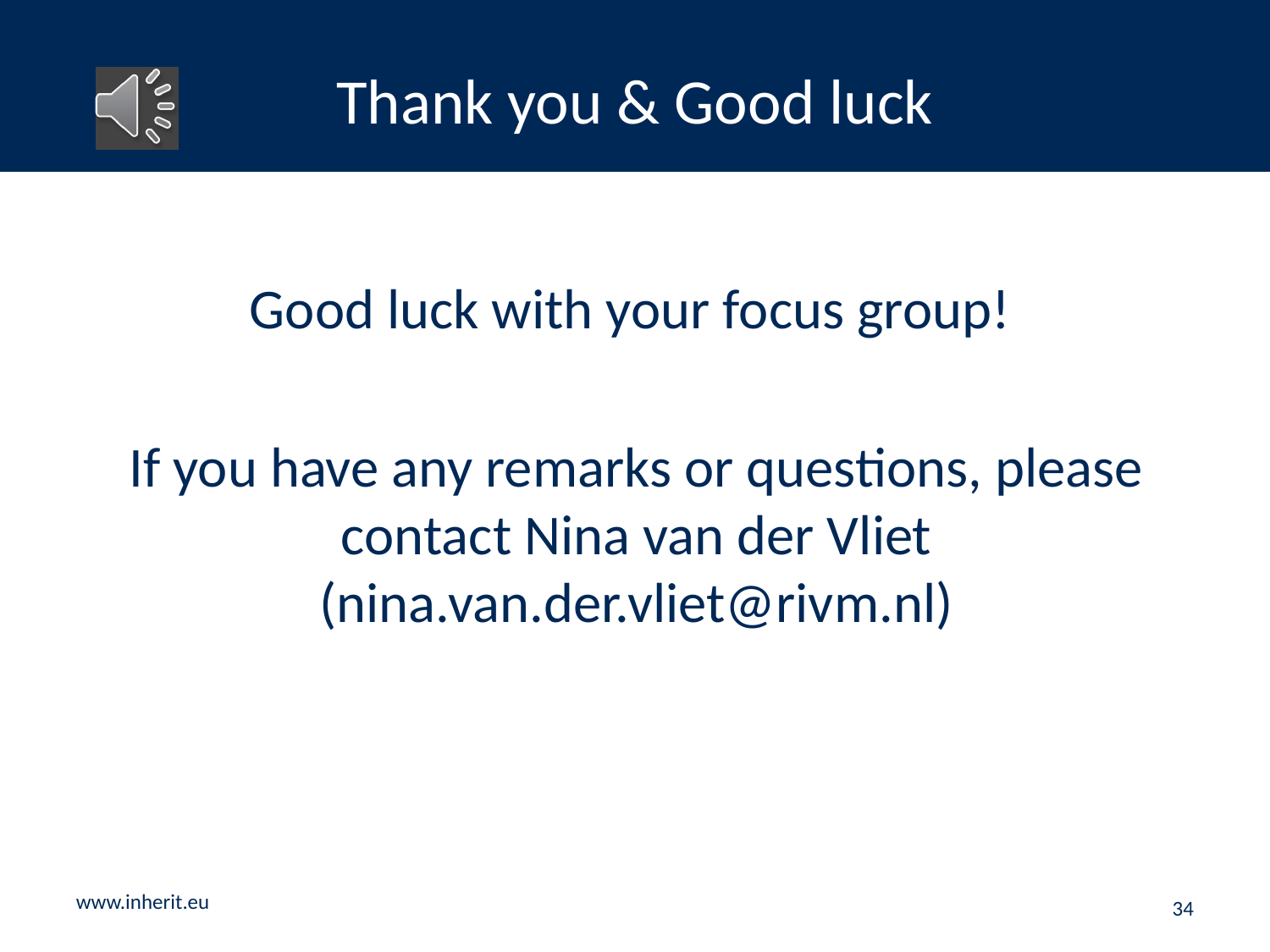

# Thank you & Good luck
Good luck with your focus group!
If you have any remarks or questions, please contact Nina van der Vliet (nina.van.der.vliet@rivm.nl)
www.inherit.eu
34

## Slide 35
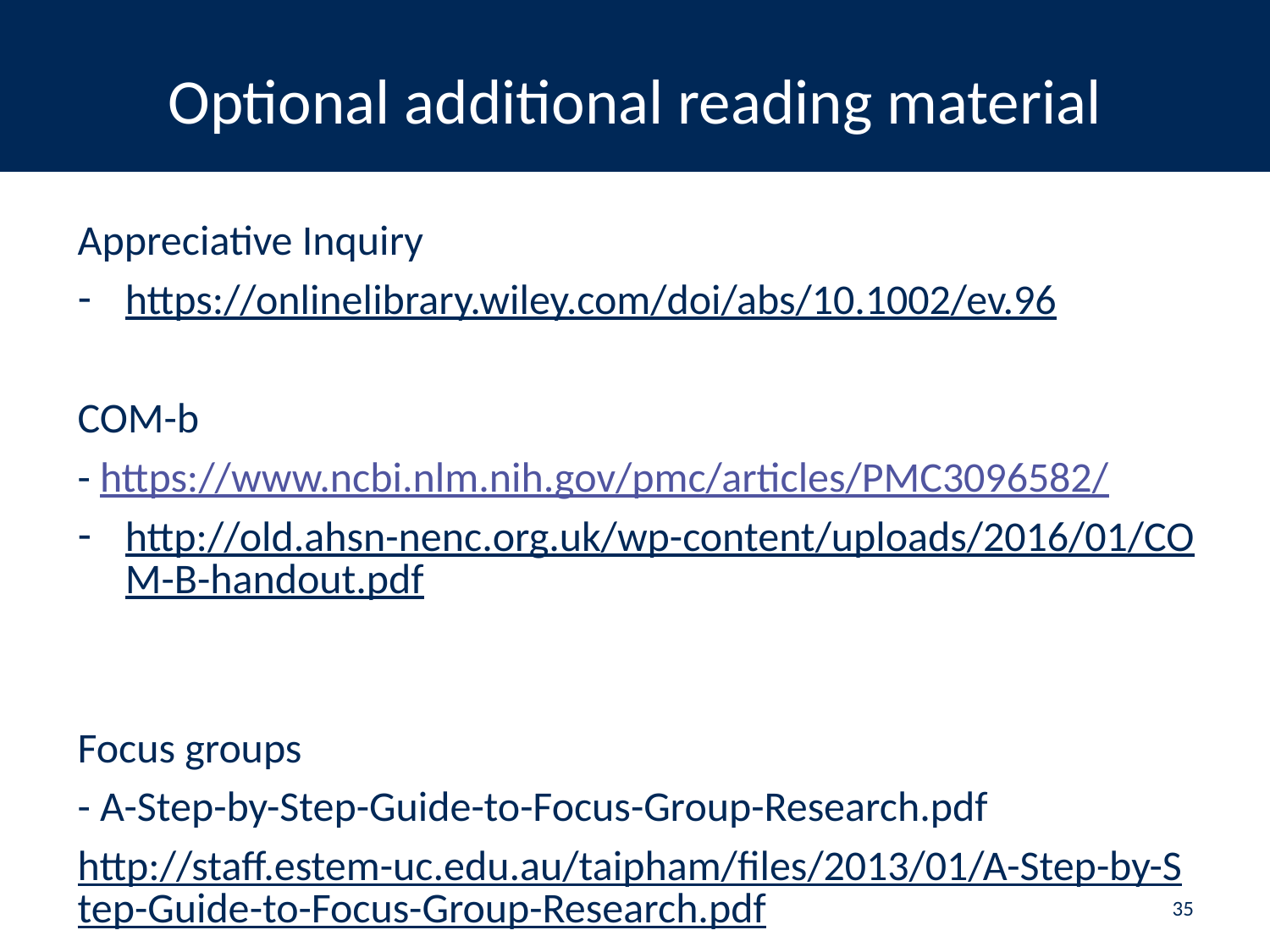

# Optional additional reading material
Appreciative Inquiry
https://onlinelibrary.wiley.com/doi/abs/10.1002/ev.96
COM-b
- https://www.ncbi.nlm.nih.gov/pmc/articles/PMC3096582/
http://old.ahsn-nenc.org.uk/wp-content/uploads/2016/01/COM-B-handout.pdf
Focus groups
- A-Step-by-Step-Guide-to-Focus-Group-Research.pdf
http://staff.estem-uc.edu.au/taipham/files/2013/01/A-Step-by-Step-Guide-to-Focus-Group-Research.pdf
35
