# Supplementary material for: Intersectoral Cooperation in 12 European Case Studies Aiming for Better Health, Environmental Sustainability, and Health Equity: Protocol for a Qualitative Evaluation
Source: JMIR Res Protoc. 2020 Jun 24;9(6):e17323. doi: 10.2196/17323 (PMC7381080; doi:10.2196/17323)
Supplement: Multimedia Appendix 3 [file resprot_v9i6e17323_app3.pdf]

Inter-Sectoral Health and Environment Research for Innovation
Qualitative Evaluation WP5 - Focus Group Research

Handbook for focus groups on (intersectoral) cooperation

Handbook for coordinators, moderators and note-takers

RIVM
July 2018

The INHERIT project (www.inherit.eu), coordinated by EuroHealthNet, has received funding from the European Union's Horizon 2020 research and innovation programme under grant agreement No 667364.

Table of Contents

Handbook for focus groups on (intersectoral) cooperation 1
1) Goal of this handbook 4
2) Overview 4
    For whom has this handbook been written? 4
    Pilot study 4
    Responsible INHERIT partner team member 4
    What information can be found in this handbook? 5
3) Qualitative evaluation & Focus groups 5
    Objective of the qualitative evaluation 5
    Objective of the focus groups 6
    What is a focus group? 6
    Why focus groups? 6
    Time and structure of focus groups 6
4) Coordinator instructions: preparing your focus group 7
    Selecting a date & inviting participants 8
    Selecting a venue 8
    Selecting a team: moderator & note-taker 8
    Materials & Documents 10
5) Theory: Appreciative Inquiry, Intersectoral Cooperation, COM-B 10
    Why is the focus group approach inspired by appreciative inquiry? 11
    Principles of Appreciative Inquiry 11
    Four phases 12
    Critical Appreciative Inquiry 13
    Intersectoral Cooperation 13
    COM-B Behavioral system 14
6) Structure and questions during the focus group 15
7) Moderator instructions 17
8) Note-taker instructions & reporting your focus group 22
9) Additional reading material 24
    Appreciative Inquiry 24
    COM-B 24
    Focus groups 24

10) Appendices 25
Appendix A Moderator Script 25
Appendix B Note-taking/Reporting form.. 31
Appendix C Participant Name Coding Table 34
Appendix D Note Checking Form 35
Appendix E Participant Sign-in Sheet.. 39
Appendix F Informed Consent Form... 40
Appendix G Invitation letter to focus groups 41
Appendix H COM-B elements 42
.... 43
Appendix I Checklist for Coordinator of Focus group.. 43
Appendix J Checklist for moderator of the focus group.. 45

1) Goal of this handbook

The goal of this handbook is to enable the RIVM and pilot partners to organize, perform and report the qualitative evaluation via the focus groups in a consistent, comparable way, generating data that can be analyzed in a standardized way. Partners are encouraged to read this handbook, and contact the RIVM team if they see any things that need adapting to their country/culture context. Some adaptation because of local context is possible, but only if discussed first with the RIVM team.

2) Overview

For whom has this handbook been written?

This INHERIT Handbook for focus groups on (intersectoral) cooperation is meant for coordinators of the focus groups (each coordinator will have to organize **one** focus group). These coordinators will mainly be the INHERIT pilot partners themselves. A few INHERIT partners have indicated that a hired agency will organize the focus group on their behalf (in yellow). This handbook is also meant for the moderator and the note-taker of the focus group, as it provides the necessary material to perform their tasks during the focus group. The theoretical section in this handbook on appreciative inquiry, behavioral determinants and intersectoral cooperation will provide everybody involved with the right contextual information. In addition, every INHERIT partner mentioned below is responsible for informing the moderator and note-taking with additional context about their specific pilot.

Pilot study	Responsible INHERIT partner team member
1. Food garden	Nina van der Vliet (RIVM)
2. Gardening with green gyms for meat free Mondays	Matluba Khan (UCL)
3. GemuseAckerdemie	Rosa Strube (CSCP)
4. Ghent en Garde	Marjolijn Vos (Gezond Leven)
5. PROVE	Daniela Craveiro (ISCTE)
6. Restructuring residential areas	Anne-Sophie Merritt (FOHM)
7. Restructuring green space	Hanneke Kruize (RIVM)
8. Sustainable schools in public schools	Pablo Martinez (UAH)
9. Thinking Fadura	Silvestre Garcia (BC3)
10. Place Standard Tool Riga	Diana Koerna (Riga City Council)
11. Place Standard Tool Macedonia	Dragan Gjorgjev (IJZRM)
12. Urban Cyclers	Vojtech Maca (CUNI)
13. Eco inclusion	Yvette Shajanian Zarneh (BZGA)

What information can be found in this handbook?

This handbook has been developed to provide information and practical guidance for the coordinators of the focus groups. In this handbook, all information from the webinar can be found, with additional details. It will provide coordinators with the necessary steps they need to take to organize the focus group, including practical arrangements and reporting. This handbook will provide moderators with instructions on how to facilitate the focus group, including a script they use during the focus group, but also practical information and tips and tricks on how to run a smooth focus group. Finally, the handbook provides the note-taker of the focus group with information and a form for reporting.

The handbook also provides theoretical information about the approach that has inspired the focus groups (Appreciative Inquiry), intersectoral cooperation, and information on behavioral determinants.

The appendices of the handbook contain the necessary documents to organize the focus group:

Appendix A Script for moderator during focus group
Appendix B Note taking form
Appendix C Participant name coding sheet
Appendix D Note checking form
Appendix E Participants sign- in sheet
Appendix F Participant informed consent form
Appendix G Invitation letter to focus groups
Appendix H Com-B elements
Appendix I Checklist for coordinator
Appendix J Checklist for moderator

3) Qualitative evaluation & Focus groups

Objective of the qualitative evaluation

The goal of the qualitative evaluation is to gain insight into the factors for success and failures of the pilot process, inter-sectoral cooperation and the possibilities for translation to other situations. As we want further insight into successful implementation of inter-sector initiatives, the most important focus point of the process evaluation is inter-sectoral cooperation (cooperation between different sectors, but also for example public-private collaboration). Experiences in inter sectoral work for health have shown that such cooperation is not self-evident. Therefore, it will be very interesting to gain insights from different angles about how inter-sectoral cooperation can be successfully organized to reach the triple-win of health, sustainability and equality goals, in the different fields of moving, living and consuming.

Objective of the focus groups

The goal of the focus groups is to qualitatively evaluate and gain a more complete, detailed image of (intersectoral) cooperation processes and goal attainment by gaining insight into underlying factors, motivations and opinions. In addition, the goal is to build capacity and motivation for future cooperation.

What is a focus group?

A focus group discussion is a structured discussion used to obtain in-depth information from a group of people about a particular topic, in our case (intersectoral) cooperation. The purpose of a focus group is to collect information about people's opinions, beliefs and attitudes, perceptions, not to come to consensus or make a decision. Focus groups typically consist of 5-10 persons. For the INHERIT focus groups, 6 to 8 persons is the optimum size. Experience has shown that focus groups of this size perform best, because everyone has the opportunity to share insights and yet the group is large enough to provide diversity of perceptions.

Why focus groups?

Focus groups are useful because they:

- are a means of gathering in-depth information and building on ideas
- focus on experience and gaining information on what people think and feel
- are flexible
- are face-to-face interaction
- are less time intensive than one-on-one interviews
- cost relatively little.

Focus groups are mostly conducted for:

- Exploring respondents' behavior, experiences, ideas, beliefs, perceptions, attitudes, feelings, and reactions in a social setting
- Generating hypotheses
- Finding common language
- Revealing group dynamism and norms
- Brainstorming (product-ideas/concepts, communication-ideas/concepts, …)
- Developing questions or concepts for questionnaires
- Early prototyping

This makes the focus group method very useful for the goals of the qualitative evaluation of INHERIT pilots.

Time and structure of focus groups

The focus groups are planned to last a maximum of 2 hours, of which approximately 80 minutes will be used for the process evaluation on inter-sectoral cooperation (including warm-up

questions and wrap-up). Thirty minutes of the time can be used for additional evaluation aspects, such as effect evaluation or non-inter-sectoral cooperation aspects. See Table 1 for the structure and timeline of the focus groups.

Table 1. Structure and Timeline of the focus group

Part	Length	Questions for moderator are prepared by
3.1) Introduction & Warming up	15 minutes	RIVM
3.2) Introductory questions	10 minutes	RIVM
3.3) (Intersectoral) Cooperation<br>3 questions	3x 15 = 45 minutes	RIVM
3.3) Wrap- up Cooperation part	10 minutes	RIVM
3.4) Additional questions	30 minutes	Pilot partner
3.5) Wrap up total focus group	5 minutes	RIVM
		Total = 115 minutes (+/- 2hours)
3.6) Debriefing meeting	20- 30 minutes	Discussion between moderator and note-taker

4) Coordinator instructions: preparing your focus group

For your focus group, there are several elements that need be arranged beforehand. To check whether you, the coordinator, have arranged all the necessary steps and materials to successfully perform the focus group, we have made a checklist. See Appendix I for this checklist.

Steps that need to be taken by the coordinator beforehand include:

- Selecting a date
- Inviting participants (6-8 maximum)
- Selecting a venue

- Selecting a moderator & note-taker
- Bringing the necessary documents and materials
- Arranging rewards

Selecting a date & inviting participants

As most focus groups will take place between September and December, it is important that the coordinator sends out invitations and reserves a venue on short notice after receiving and reading this handbook, as soon as possible. It might be best to send out a few dates to participants, to check on which date the most essential participants are available. We understand it is holiday season, but invitations should be sent in August or very early September. Please keep in mind that the maximum group size is 6 to 8 participants.
In the Appendix, an invitation letter template can be found, which should be translated and expanded with specific information about your pilot.

Selecting a venue

Selecting an appropriate location and venue to hold focus groups is important as it can affect the recruitment of participants. Key issues associated with successful recruitment are:

- transport – the focus group must be easy for participants to travel to and get in and out
- access – consider wheelchair and pram access, parking and access to public transport

The location needs to be safe for participants and facilitation staff. This also includes considering the time of day that the focus groups take place. The venue should be close to toilets and have facilities to prepare a snack and tea and coffee. Ideally, offer refreshments before or after the focus group, as snacking during the discussion may affect participation. It is a good idea to have water and tissues available.

Importantly, the focus group needs to be held somewhere where you will not be disturbed. Noise levels and visual and other environmental distractions should be reduced. Especially when recording focus group interviews, do a quick test of the audio in your room to make sure that the recording level is adequate for the background noise levels and for participants' natural speaking volume.

Selecting a team: moderator & note-taker

The following team members are required to organize a focus group:

- Project coordinator. Someone from the INHERIT team of the INHERIT partner
- Moderator
- Note-taker
- Observer (optional). Can be another person from the INHERIT team of your institute

The moderator can be another person from the INHERIT team of your institute or a hired contractor (for now, only PST Macedonia and PST Riga will subcontract).

The note-taker can be another person from the INHERIT team of your institute. The coordinator can function as an observer (optional) during the focus group.

The moderator directs and guides the discussion and is responsible for ensuring that everything runs on track. Most importantly, the moderator is responsible for ensuring a safe and supportive consultation for participants. The moderator must be able to maintain the flow of the session, address problematic group dynamics (should they occur), and ensure that participants feel comfortable in discussing issues with each other. The moderator guides the discussion but should not be the center of the process.

! An internal moderator should not be a colleague who works with the participants on a daily basis, because the moderator must be objective and comfortable discussing all experiences.

Key role of the moderator:

- able to understand fully the research objectives
- make participants feel at ease
- build trust
- listen
- be alert
- be flexible (without losing focus)
- show sensitivity
- observe
- be attentive for nonverbal behavior
- be able to link reactions together
- encourage participation of each participants
- facilitate/stimulate interaction between respondents
- challenge group members
- keep control over the conversation
- probe
- synthesize

The following characteristics need to be taken into account when selecting a moderator;

- aware of the needs of the topic
- non-judgmental
- skilled in active listening
- respectful of participants
- open-minded
- observant
- patient and flexible.

Materials & Documents

Ten days before the focus group is planned, the coordinator is encouraged to go through the checklist in Appendix I, to check whether all arrangements are made and all materials are translated and available to be taken to the focus group. Important materials that need to be prepared beforehand for the focus groups include:
(number between brackets indicates the number of copies that should be brought to the focus group)

- Translated Script for moderator and note taker, and coordinator (3)
- Translated Note taking form (1) & notebook (1) & coding sheet participants (1) (digital/paper). Also when making digital notes, bring a paper version as a back-up
- Translated Participants sign-in list (1)
- Translated Participants informed consent form (one for each participant so about 8)

- Audiotape recording material (& back-up) (2)
- Sticky notes and pens for all participants (10)
- Handbook for coordinator (1)
- Checklist (2) (one for moderator, one for coordinator)
- Special attention topic sheet for moderator and note-taker (2)
- A reward (optional) for participation

For a complete list, see the Appendices (part 10).

5) Theory: Appreciative Inquiry, Intersectoral Cooperation, COM-B

It is important that the coordinator, moderator and note-taker all know what appreciative inquiry is, and why RIVM has chosen this approach to inspire and guide the focus group.

As INHERIT is a future oriented project exploring new ways to generate healthy and sustainable behavior, the methodology for the qualitative evaluation needs to be future oriented as well. The focus in the qualitative evaluation is, therefore, not solely on gathering data about what happened in the various pilots , but also on determining what steps could be taken to perform even better in future application of the interventions. Moreover, we will look for similarities and differences between pilots to develop both generic recommendations for effective triple win interventions and specific ones for various interventions/contexts. We propose a procedure and method building on Appreciative Inquiry (Cooperrider, Whitney et al. 2003).

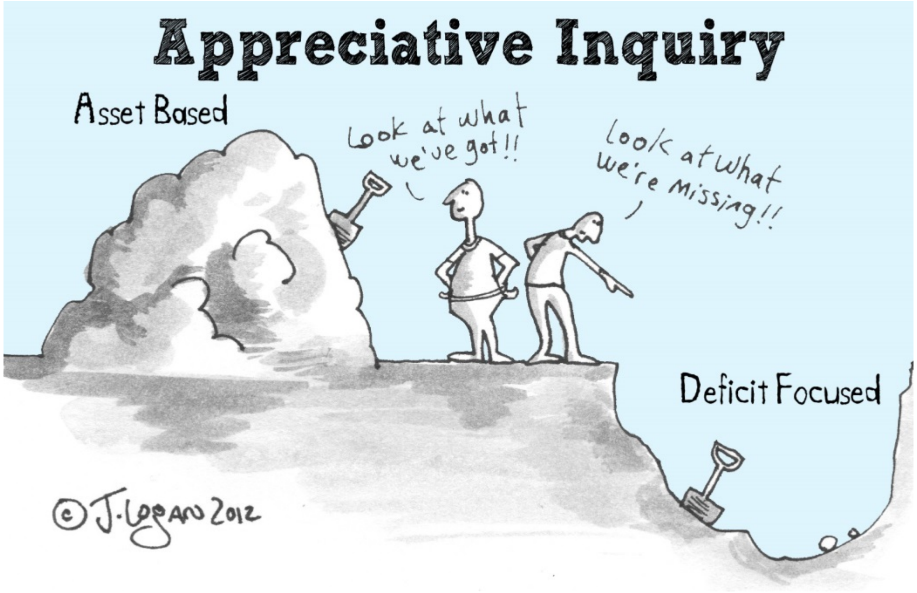

Appreciative inquiry is a strength-based approach to data gathering. Instead of focusing on problems, and what is going wrong, it focusses on questions such as: "*what works well around here, and how can we do more of it?*" It uses a deep understanding of moments when we have been at our very best as a launching point for future action. It helps people identify what they want to see more of, to create a shared vision of the future and to make that vision become reality.

Why is the focus group approach inspired by appreciative inquiry?

Research has shown that people who experience positive feelings are more flexible, creative, integrative, open to information and efficient in their thinking.
Also, a ratio of more positive than negative talk is related to the quality of relationships, cohesion, decision-making, creativity and overall success of various social systems. Appreciative inquiry creates a momentum for change by talking about what goes well and what could be done in the future[1].

Principles of Appreciative Inquiry

Some important principles of appreciative inquiry will now be explained, to help you get a better idea of what appreciative inquiry means.

[1] (Isen, 2000; Fredrichson & Losada, 2005)

Principle	Summary	Details
Constructionist Principle	Words create worlds	Reality, as we know it, is a subjective vs. objective state and is socially created through language and conversations.
Simultaneity Principle	Inquiry creates change	The moment we ask a question, we begin to create a change. "The questions we ask are fateful."
Poetic Principle	We can choose what we study	Teams and organizations, like open books, are endless sources of study and learning. What we choose to study makes a difference. It describes – even creates – the world as we know it.
Anticipatory Principle	Images inspire action	Human systems move in the direction of their images of the future. The more positive and hopeful the image of the future, the more positive the present-day action.
Positive Principle	Positive questions lead to positive change	Momentum for [small or] large-scale change requires large amounts of positive affect and social bonding. This momentum is best generated through positive questions that amplify the positive core

– From Cooperrider, D.L., & Whitney, D. A Positive Revolution in Change: Appreciative Inquiry. Taos, NM: Corporation for Positive Change, 1999.

Regarding our subject of cooperation, the constructionist principle inspired us to work with images and stories, such as "how did this cooperation begin, how did it develop to what it is now". The simultaneity principle means that inquiry creates changes. This means that when talking about what cooperation should look like, what is important and what is currently going well, people gain insights in potential actions that contribute to cooperation, allowing them to act upon these insights.

Four phases

Appreciative inquiry consists of four phases, but these phases do not come after each other, they take place within an iterative process.

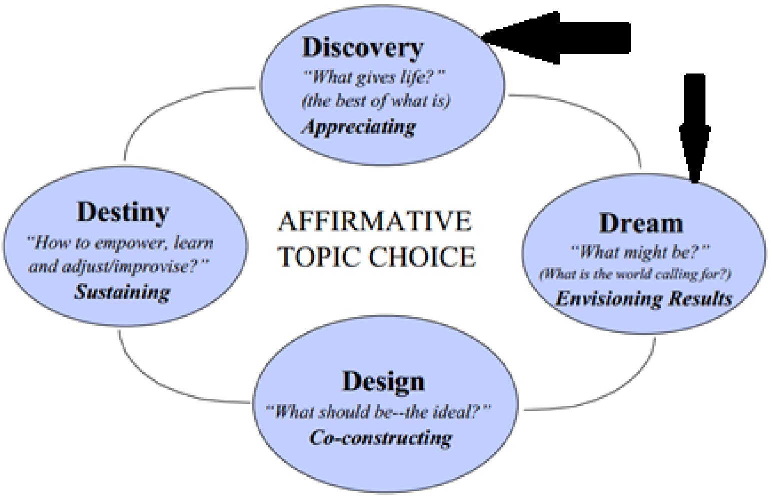

As the main goal of the focus group is evaluation and not transformation, the focus is on Discovery: appreciating the best of what is, and less focus on the Dream, Design and Destiny phases, which focus more on envisioning results and co-constructing a design.

Critical Appreciative Inquiry

A common criticism on appreciative inquiry is that it is unwilling to look at negative side, at what is going wrong, discouraging criticism. Appreciative Inquiry does address issues and problems, but from a different and often more constructive perspective: it reframes problem statements into a focus on strengths and successes. For example, rather than ask participants to list the problems their organization is facing, they are asked to explain what is going well, why it is going well, and what they want more of in the organization. In some Appreciative Inquiry efforts, participants are also asked to state their specific wishes for the organization. This implicitly raises and addresses problems.

Problems and weakness can and do emerge when using appreciative inquiry, and should not be ignored. Instead, barriers and downsides can be discussed when asking : “what might have been?” or “what to avoid?”. In the INHERIT focus groups, we will ask what is missing and what might have been. This provides insights into what creates the gap between what participants want versus what they see happening, and may lead to identifying barriers.
See Moderator tips in section 8 for more information on how to deal with negative reactions.

Intersectoral Cooperation

What do we mean by intersectoral cooperation?

It is the cooperation between
- parties from different sectors (such as health or environmental sectors);
- parties from private and public sectors;
- parties from different types of institutes or organizations (for example, NGO’s);
- parties from different levels of government (neighbourhood, community, local, regional, national).
- professionals and citizens

Goal of intersectoral cooperation

What is the goal of intersectoral cooperation? It is to bring actors from different parties together to achieve mutual understanding on an issue and negotiate and implement mutually agreeable plans for tackling this issue/challenge. Each actor possesses distinctive assets that can be combined in a productive manner to solve complex problems.

Cooperation

Some pilot projects focus more on cooperation between different sectors (interdisciplinary), others more on cooperation between professionals and citizens, and others between the

private and public sector. Because of this, it may be less confusing if it is communicated to participants that the focus group is about cooperation in the context of the project. In the focus groups, please use the term 'cooperation'.

Clarify in advance what the type of intersectoral cooperation is that your pilot project is about, and what your focus group will be about. Make sure intersectoral elements are part of the discussion on cooperation, as we want to gain more insight in working intersectorally.

COM-B Behavioral system

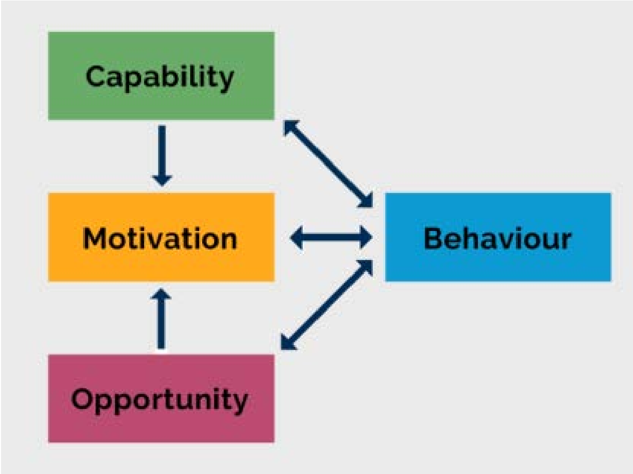

The COM-B is a behavioral system with three behavioral components that determine behavior (and in turn are affected by behavior). These components are capability, opportunity and motivation. In analyses of the focus groups, the COM-b will one of the guiding models. Therefore, it is important that moderators and note-takers are aware of these behavioral influencers during the focus group.

Capability can be psychological: having the necessary knowledge and skills about what kind of other local companies or initiatives there are in the area. It can also be physical, which is more about being physically able to perform a certain behavior, such as being good with computers and social media to reach out to others, or having strong social skills.

Opportunity, is about the context in which populations and individuals behave. These can be physical opportunities, such as having local initiatives very close to each other, facilitating cooperation. In addition, social opportunities, such as having a historically strong network of companies in an area.

Motivation entails all the brain processes that energize and direct behavior, and these are influenced by capability and opportunity . When someone has a positive attitude towards cooperation, and consciously decides to make this happen more, this is reflective motivation.

However, a lot of behavior is habitual, and we just do what we do, because we have always done it that way. This is more automatic motivation, more unconscious processes.

In Appendix H, a sheet explaining the COM-b elements can be found. These should be taken to the focus group, as both the moderator and the note-taker should have these elements in the back of their heads when moderating and taking notes during the focus group.

6) Structure and questions during the focus group

30-60 minutes before the actual focus group time
1) Arrive in time before participants, coordinator needs to set up all materials (such as audio recording material, pens and sticky notes, note-taking desk, timers, refreshments).

Minutes 0- 10 (Welcome & Consent)
2) Welcome all participants, make them sign the sign-in form, and give them some time to read, sign and ask questions about the informed consent form, and to put their name cards in front of them (In the meantime, note-taker makes a seating map with acronyms for participants)

Minutes 10- 20 (Start focus group, introduction, introductory round)
3) After everybody has signed and handed back the consent forms, start the audio taping and the focus group. Welcome and thank participants for coming, introduce yourselves and state the purpose of the focus group, a short explanation of the project, and how long the session will take. Also explain some ground rules, including the Chatham House Rule (see part 7 for more information about this rule). In addition, inform participants that their mobile phones should be turned off or put on mute. Then explain that this focus group is inspired by Appreciative Inquiry and what that entails.

Start the introductory round, starting yourself. Answer the question: "*What is your name, and what is a positive experience you have had today?*"

Minutes 20- 35 (Introductory questions cooperation)
4) Ask the introductory questions:
- *How did the cooperation/project start, how did it develop to where it is now? What contributed to the cooperation process?*

➔ Method: ask the whole group and guide a short group discussion
Prompt participants to tell personal stories about their experiences with cooperation.

Minutes 35- 80 (Core questions cooperation)
5) Ask the three core questions:

(1)What are the core factors that make this cooperation happen, that energized and inspired cooperation? *"Describe a peak experience in (intersectoral) cooperation in [project X], when you felt really engaged and motivated"*
→ Method: Ask participants to write down their answer on sticky notes (5 min). Make sure participants write Q1 on the sticky notes. Follow this by a group discussion, asking participants to share their answer, and explicitly ask after an answer, if someone has written down something very different from what he or she has heard so far. Prompt participants to tell personal stories about their experiences with cooperation. You want to know What is it about this project, - its structure, systems, processes, policies, staff, leaders and strategy, that creates conditions where cooperation can flourish?

(2) *How could the cooperation have been? What would you change if you could change anything in this cooperation? What could it still become?*
→ Method: guide group discussion

(3) Where do you want to be between now and a certain period, what does this future look like? What are possible options (actions, projects) to reach this and enhance cooperation in the future? Be ambitious! *Vivid proposition: "If your dream is X, what would you want to have accomplished in Y years?"*
→ Method: Ask participants to write down their answer on sticky notes (5min). Make sure participants write Q3 on the sticky notes. Follow this with a group discussion, asking participants to share their answer and guide the discussion.

Minutes 80- 90 (Wrap- up cooperation part)
6) Wrap up the cooperation discussion, and ask participants: "*Of all the things we discussed, which one is the most important to you*?"
Summarize the discussed, and ask if this is accurate.
Ask if you have missed anything (or anyone).

Minutes 90-120 (Optional additional questions)
7) In this part, the INHERIT partners who have indicated that they want to ask additional questions that are not about cooperation, or that are effect evaluation, can be asked during these 30 minutes. It should be kept in mind that these questions should be answerable by the stakeholders that are participating in the discussions on intersectoral cooperation, which may limit the scope of topics.

Minutes 120-125 (Wrap-up)
8) Wrap-up of the whole focus group discussion
Summarize and ask if accurate. Thank all participants, point them to travel expenses and optional reward, make sure they hand in all sticky notes with answers.

Minutes 125-155 (Debriefing meeting)
9) Moderator & note-taker hold a short meeting immediately after the focus group

- To discuss outcomes, clear confusions, check interpretations

1) How did it go?
2) What are the most important results?
3) What things stood out?
4) What have we learned from today, from what we've heard?

7) Moderator instructions

In Appendix A, a script for the moderator is attached that should be used to facilitate the focus group. Materials to be used can also be found in the Appendices, such as participants sign-in sheet (Appendix E) and participants informed consent form (Appendix F). In Appendix H, COM-b elements are explained, as these elements should be held in the back of your mind when facilitating the focus groups. In Appendix J, a checklist for the moderator is attached, where some important steps and preparations can be found that should be completed before, during and after the focus group.

Instructions to create an open, positive atmosphere where everyone gets to speak their opinion

The Chatham House Rule

This rule originated at Chatham House with the aim of providing anonymity to speakers and to encourage openness and the sharing of information. It is now used throughout the world as an aid to free discussion. By introducing the Chatham House Rule, people should get the feeling that they are anonymous and can speak their minds freely and openly [2].

"When a meeting, or part thereof, is held under the **Chatham House Rule**, participants are free to use the information received, but neither the identity nor the affiliation of the speaker(s), nor that of any other participant, may be revealed."[2]

Sticky Notes

By using sticky notes, everyone's opinion can be taken into the evaluation analyses. It can also help those who are more quiet to speak their minds, when they get the change to first write it down, and then answer when asked their opinion. Ask those that do not volunteer to share their answer as well, to avoid only hearing answers of the more dominant participants.

There is no pressure by the moderator to reach group consensus, instead attention should be paid to understanding the feelings, comments and thought processes of participants as they discuss the topics.

[2] https://www.chathamhouse.org/chatham-house-rule

Opening round

Very important for the focus group to get everyone talking, as the longer it takes before someone says something in a group, the less likely he or she is to say anything.

What if participant does not want to sign the informed consent form, or to be audio taped?

- If a participant does not want to sign the informed consent form, they cannot participate in the focus group. He or she should be compensated for travel expenses, and can leave.
- If a participant does not want to be audiotaped, you can try whether he or she would agree that when the report is out, he or she can first look at the report and have the chance to indicate what he or she wants deleted from the report that he or she said. If he or she does not agree with this as well, then he or she should be asked to leave (but travel expenses should be compensated).

Tips regarding Appreciative Inquiry

As the focus group is inspired by Appreciative Inquiry, here are some tips on how to pose engaging, appreciative questions[3]:

- State questions in the affirmative
- Invite participants to use storytelling and narratives: when you begin to address the topic of cooperation, start with specifics relevant to the person interviewed. Try to get him or her to tell story. This is basically what we try to reach by asking participants to: "*Describe a peak experience in (intersectoral) cooperation, when you felt really engaged and motivated*"
- Value "what is" (what has happened so far, the state of cooperation, do not judge but appreciate)
- Spark the appreciative imagination by helping the person locate experiences that are worth valuing (ask participants to think back to successes or positive experiences)
- Convey unconditional positive regard
- Evoke essential values, aspirations and inspirations
- A question should A) evoke a real personal experience and narrative story that helps participants see and draw on the best learnings from the past, and B) help envision the best possibility of the future

How to deal with negative reactions?

---

3 **The Appreciative Inquiry Handbook: For Leaders of Change** David Cooperrider, Diana D. Whitney, Jacqueline M. Stavros

As mentioned earlier, problems and barriers do and can emerge in an appreciative inquiry section. People should feel free to talk about things they believe require fixing. There are several ways to deal with problems, barriers and other negative aspects[3].

- Listen: if someone feels real intensity about what he or she wants to say about issues, let the person say it, but maintain a caring and affirmative spirit.
- Redirect: when you have listened, find a way to redirect back to the task at hand after paraphrasing.
- **Preferably, postpone**! Tell them you would like to get back later to what they have said, when you get to the question "how could the cooperation have been". If someone reacts with a problem, this implies images of an ideal someone may have. The moderator should listen to this problem, and then tell the participant that discussion on this will be take place later, when the moderator asks Q3: What would you change if you could change anything in this cooperation?

General Moderator Tips

The following tips for success from CSCP's INHERIT focus group guideline are very useful to read for moderators, as they provide guidelines on how to run a focus group smoothly.

What do you need to pay attention to:

• Dawn Dominator (monopolizers who dominate discussions)
• Negative Ned (Idea zapper, who is negative and not open for new ideas and input)
• Off track Tron (who continuously directs the discussion towards different topics)
• Suzy Sunshine (who does not see any barriers, flaws, negativity)
• Quiet Quinton (hesitators who do not speak out)
• Experts (who feel they know everything)
• I'd rather be somewhere else
• Emotional reactions

Encourage discussion:

- Maintain a warm and friendly attitude.
- Establish eye contact with participants (if culturally appropriate).
- Do not judge participants' responses (verbally, body language).
- If a participant gives a vague answer: probe, e.g. "Could you say a little more about that?" or "Would you give me an example of what you mean?" "Please describe what you mean."

Keep the group focused:

- If the group is getting too far off the topic, remind the group of the original question by summarizing the responses and then repeating the question.
- If the group is finding the question difficult to answer, rephrase the question.

Keep individual participants from dominating the conversation:
- Try avoiding eye contact with dominant participants.
- Remind the group that everyone's opinion is important.
- Acknowledge the response of the dominant participants and redirect the discussion by asking other participants for their opinions.
  - ➔ Often dominant talkers are spotted in pre-session small talk. Try to seat the dominant individual beside the moderator, in order to exercise control by the use of body language. Avoid eye contact with the dominant talker, and invite others actively to contribute by asking "Are there others who wish to respond to this question?" "Does anyone feel differently?"

Encourage shy participants to contribute:
- Making eye contact with quieter participants.
- Gently ask quieter participants for their opinions during pauses in the conversation by addressing them by name.

Be aware of group pressure:
Probe for alternate views from the group, e.g., "That is an interesting viewpoint. Let's also explore some alternatives." Monitor time closely.

Do:
- stay calm and take deep breaths
- acknowledge the emotion. Name it and recognise its origin
- focus on the issue. Take it seriously and pay full attention to what the participant is saying. Try to understand the source of their anger or distress
- acknowledge and explore the participant's emotions. Make the participant feel that you have heard what they have said and will not judge them. For example, say. 'I can see that you are very upset and I would like to find out more about what happened to make you feel that way'
- listen actively and maintain eye contact. Always listen without interrupting. You can then paraphrase the information supplied and ask clarifying questions
- identify the participant's key concerns. For example, say 'Can I just check that I have heard you correctly… you said that…'
- apologise if appropriate, for example, 'I can see why you're upset and I would be too. I'm sorry that happened to you in hospital'
- move on to suggestions regarding how the problem can be overcome and the situation improved
- recognise if you have been hearing about a situation that requires further action or reporting and work with the participant or carer to support this process

Do not:

- take it personally as this is not about you. Participants who appear difficult or angry are acting this way as their needs have not been met. Try to visualise angry comments going through you as a messenger only
- get defensive. Do not challenge or criticise the participant. It is more important that you try to understand the root cause of the anger
- get into an argument or yell back at the participant
- be tempted to fix the problem before the participant has finished talking.

What to do when you encounter a difficult situation?

You may encounter several types of angry emotions, expressed in different ways, during the course of the discussion. A participant may:

- appear withdrawn and unwilling to share information, or present information in a vague manner
- be very critical or negative about aspects of their experience
- intimidate others through use of sarcasm
- appear sad or depressed.

When dealing with difficult or angry situations, it is important to remember that anger:

- is a common and normal reaction
- often results from a loss of control and feelings of powerlessness
- can be justified and should be managed accordingly.

Participant anger also has several positive features:

- it signals an important problem which needs attention
- it acts as a motivator to find solutions and bring about change.

What to do when you encounter participants who become emotionally distressed?

Dealing with strong emotional responses can be extremely challenging. You must maintain a calm professional detachment and not become too involved or distressed or express inappropriate emotion. An inappropriate emotional response can stop you thinking and acting clearly and takes the focus away from the participant in distress. Similarly, participants will not disclose emotional content to someone who is trying to avoid discussing such content. Therefore, always ensure that your demeanour is as comfortable as possible in order that the participant will also be put at ease.

What to do when you encounter participants who seem overly agreeable
Some participants may be too agreeable in a discussion. They may say very little, provide only positive information or provide information that they think you want to hear.

If you encounter a participant who appears to be too agreeable, it may be helpful to:

- re-focus the participant's attention on the discussion of negative as well as positive aspects of their journey and ways to improve this
- use normalisation statements to encourage a balanced discussion, for example, 'Some stakeholders have said that they didn't have such an easy time when beginning the cooperation … was that the case for you?'
- explore subtle statements like 'They are very busy …' or 'I expect to wait …' as they may contain deeper information
- try asking what their colleagues thought about what happened, for example, 'Does your colleague also agree that everything went well with cooperating at that time?'

8) Note-taker instructions & reporting your focus group

Appendix B contains the note-taking form, Appendix C contains the name-coding form to give participants an acronym (P1 to PN-1). Before the focus group starts, when participants are filling in the informed consent form, fill in the name-coding form, and create a seating map (see below). Appendix H contains information about COM-b elements, which should be held in the back of your mind when observing and taking notes.

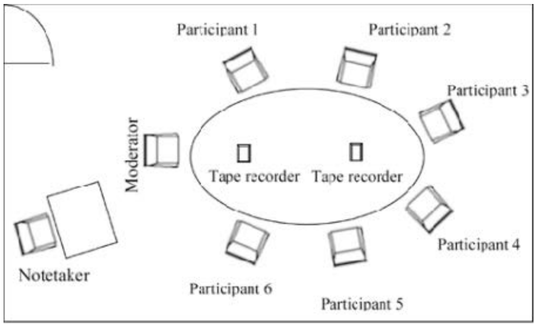

Note-taker instructions

- ➔ Clarity and consistency are important. Anticipate that others will use your notes days or weeks later when memory has faded.
- ➔ Try to type participants' statements in their own words where possible. So do not mainly summarize what people share.
- ➔ Ask for clarification if you do not understand something. If someone says something that you were not able to understand, ask them to repeat or clarify what they said. While the facilitator is responsible for leading the conversation, the note-taker should ask clarifying or probing questions

- ➔ Use the Observations Column to write down contextual notes about the focus group. Note impressions or insights that describe the "feel" of the focus group or that seemed to affect the conversation*. This column is also meant for noting down non-verbal cues from participants* – Head nodding, laughter, discomfort, pauses. Remember during

analysis that non-verbal cues can mean different things on different cultures. Make note of non-verbals but don't make assumptions about what they mean.

- ➔ Clean your notes and add or complete reflection notes after the focus group. Within 24 hours of the focus group, clean your notes so shorthand is spelled out and gaps are filled in. It can be difficult to remember what people said after 24 hours. Make sure your notes are clear and can be understood by another person who did not attend the focus group.

- ➔ *Use your notebook for big ideas, hunches or thoughts of the note-taker* – Occasionally the you will discover a new concept that will be helpful in later analysis. Jotting these down increases the likelihood they will be remembered during the analysis phase.
- ➔ *Also use your notebook for any important quotes worth highlighting*

Note-checking Instructions

<u>Note-checking is to be performed by other person, who was neither moderator nor note-taker.</u>
In Appendix D, the note-checking form can be found.
At a later time and day, the note-checker should use the note-taking form while listening to the audio file of the focus group, and fill out the note-checking form. The purpose of checking the note-taking form is to make sure that the notes accurately represent what has been said during the focus group, and any important missed notes and observations can still be added.
The note-checker should listen to the audio file and the notes, with two questions in mind:

- What has been written down, is it a good representation of what has been discussed in the focus group?
- Do you think the interpretation is correct, or do you have additional thoughts?

The following reporting steps should be conducted:

Steps	Person
1. Audio taping of focus group and concurrent note taking	Note-taker
2. Reflective debriefing in pair immediately after the focus group, add notes/observations	Note-taker and moderator
3. Checking audiotape & add notes/observations	Note-checker
4. Translate complete set of checked notes to English in the note-taking form	INHERIT pilot partner
5. Analysis of the English note-taking form	RIVM team

9) Additional reading material

Appreciative Inquiry

- An overview of appreciative inquiry in evaluation- Coghlan, Preskill & Tzavaras Catsambas
  https://onlinelibrary.wiley.com/doi/abs/10.1002/ev.96

- Appreciative Inquiry Handbok for Leader of Change – Cooperrider, Whitnet & Stavros
  https://books.google.nl/books?id=Y7j_ecbTvl0C&printsec=frontcover&dq=the+appreciative+inquiry+handbook+for+leaders+of+change&hl=nl&sa=X&ved=0ahUKEwj7qs2onrXcAhWNLFAKHat2CYkQ6AEIKDAA#v=onepage&q=the%20appreciative%20inquiry%20handbook%20for%20leaders%20of%20change&f=false

COM-B

- The behaviour change wheel: A new method for characterising and designing behaviour change interventions – Michie, van Stralen & West
  https://www.ncbi.nlm.nih.gov/pmc/articles/PMC3096582/

- COM- B handout
  http://old.ahsn-nenc.org.uk/wp-content/uploads/2016/01/COM-B-handout.pdf

Focus groups

- A-Step-by-Step-Guide-to-Focus-Group-Research.pdf
  http://staff.estem-uc.edu.au/taipham/files/2013/01/A-Step-by-Step-Guide-to-Focus-Group-Research.pdf
- Focus groups. A practical guide for applied research
- https://books.google.nl/books/about/Focus_Groups.html?id=ySKeSZeWywMC&redir_esc=y

10) Appendices

Appendix A Moderator Script

Inter-Sectoral Health and Environment Research for Innovation
Qualitative Evaluation WP5 - Focus Group Research

Moderation and questions	Aim	Time: Stick to this time!
**Welcome, ethics**<br><br>**Receive and welcome all participants together with coordinator upon arrival**<br><br>**Offer a drink**<br>**Let participants sign sign-in sheet**<br>**Let participants sign consent form**<br>**Let participants write names on name tents/ put on name cards**<br><br>**When all have signed, be explicit:** "*We will be audio taping this focus group, as described on the consent form*". **After stating this to the group, turn the audio tape on.**	Ethic requirements	Be there at least 30 to 60 minutes before the focus group takes place.<br><br>0.00-0.10 Signing of sign-in and consent form<br><br>(total duration 10 min)
**Welcome, information, introduction round**<br><br>*"Welcome everybody and thank you for taking the time to participate in this focus group today! My name is xxx and this is my colleague, who will be taking notes. My other colleague xxx, is the coordinator of the project for which we are doing this focus group today".*<br><br>*"We are here today to talk about your experiences with (intersectoral) cooperation in the context of [project X. Project X is… short introduction (3-4 sentences)"] Intersectoral cooperation is cooperation between parties from different sectors, private public sectors, different types of institutes of levels of government, and cooperation between professionals and citizens. Today, in the context of [project X], we will be talking about [choose which are most relevant of the before mentioned cooperation options]*	Welcome all participants, and thank participants for coming.<br><br>Introduce yourself & colleagues briefly<br><br>State the purpose of the focus group, and explain the Appreciative Inquiry inspired approach<br><br>State the Chatham House Rule & other discussion rules.	0.10 min – 0.20 min<br><br>(total duration 10 min)

This focus group is inspired by the Appreciative Inquiry approach. Many times we try to ask questions about things that aren't working well, the problems, so we can fix them. This may prevent learning and improvement. In this case, appreciative inquiry tries to find out what works, and find ways to infuse more of that positive core into performances. The end result will be that we understand the forces that provide vitality and strengths to this cooperation".<br><br>*"There are no right or wrong answers. We expect that you will have different points of view, please feel free to share your point of view, even if it differs from what others have said.*<br><br>*In addition, this focus group follows the Chatham House Rule. When a meeting, or part thereof, is held under the* ***Chatham House Rule****, participants are free to use the information received, but neither the identity nor the affiliation of the speaker(s), nor that of any other participant, may be revealed.*<br><br>"P*lease make sure your mobile phones are turned off or are muted*."<br><br>*"The focus group will take about [****1.5/ 2 hours****] maximum. To make sure we do not exceed this time, we keep track of time quite strictly and sometimes I will wrap up discussions to make sure we are all finished and can go home or to our next appointments!"*<br><br>**Introductory round: ask all participants clockwise to answer the question** "*What is your name, and what is a positive experience you have had today? I will start, my name is XX and my positive experience of today was …"*	Introducing yourself and start the introductory round, whilst setting a positive vibe.<br><br>Stating how long the session will be, and that time will be closely monitored.<br><br>← time depends on whether additional questions will be asked. If not, the duration will be 1.5 hours.	
**Introductory questions**: **ask the whole group and guide a short group discussion**<br><br>*"Now we will be talking about the topic of cooperation. How did the cooperation/project start, how did it develop to where it is now?*<br><br>*"What contributed to the cooperation process?"*	Getting a good idea of the development of cooperation in this project, what contributed to it.<br><br>Prompting	0.20- 0.35 min<br><br>(total duration 15 min)

**Ask participants explicitly about different experiences than what they have already heard, to get a broad insight into the process.**	participants to tell personal, vivid stories about their experiences with cooperation.	
**Core questions**<br><br>**<u>Q1</u>**<br>*"We will now proceed to the next question. What are the core factors that make this cooperation happen, that energized and inspired cooperation? "Describe a peak experience in (intersectoral) cooperation in [project X],, when you felt really engaged and motivated"*<br><br>*"We have distributed post it notes and pens, and I want to ask you to write down your answers on post it notes. Use different sticky notes for multiple experiences. Make sure you write Q1 on the upper corner of the sticky note you write your answer on. I will let you think and write it down in the coming 5 minutes"*<br><br>**Follow this by a group discussion, asking participants to share their answer, and explicitly ask after an answer, if someone has written down something very different from what he or she has heard so far. Prompt participants to tell personal stories about their experiences with cooperation.**<br><br>**<u>Q2</u>**<br>**"**Now we proceed to the next set of questions: *How could the cooperation have been? What would you change if you could change anything in this cooperation? What could it still become??"*<br><br>**→ Method: guide group discussion, again explicitly asking people to tell their experiences and ask for who had the same, or different opinions**<br><br>**<u>Q3</u>**<br>*"Now we will continue to the last question about cooperation, which is about the future. Where do you*	You want to know what is it about this project, - its structure, systems, processes, policies, staff, leaders and strategy, that creates conditions where cooperation can flourish?<br><br>You also want to know what can still improve, what people would change, what barriers may still exist	0.35- 1.20 min<br><br>(total duration 45 min)

want to be between now and a certain period, what does this future look like?<br><br>*What are possible options (actions, projects) to reach this and enhance cooperation in the future? Be ambitious and vividly!"*<br><br>*"If your dream is X, what would you want to have accomplished in Y years?"*<br><br>*In the following 5 minutes, write down the answer on sticky notes, noting Q3 in the upper right corner*<br><br>**Follow this by a group discussion, asking participants to share their answer and guide a discussion.**		
**Wrap up cooperation part**<br><br>**Wrap up the cooperation discussion, and ask participants**: "*Of all the things we discussed, which is the most important to you*?"<br><br>**Summarize the discussed, and ask if this is accurate. Ask if you have missed anything (or anyone).**	Retrieving what is most important about cooperation in the project<br><br>Summarizing and checking summary	Min 1.20-1.30<br><br>(total duration 10 min)
**Optional! Additional part with partner specific questions**<br>**Only when the coordinator of INHERIT has indicated that there will be a part with additional questions. These questions should be added to the moderator script below:**<br><br>It should be kept in mind that these questions should be relevant and answerable by the stakeholders that are participating.	Retrieving information about additional questions by partner	Min 1.30- 2.00<br><br>(total duration 30 min)
**Wrap up focus group**<br><br>**Summarize and ask if accurate. Thank all participants, point them to travel expenses and give them the optional reward (check with coordinator if there is a reward), make sure they hand in all sticky notes with**	Summarizing and checking summary<br><br>Thank participants, retrieve sticky notes	(Total duration 5 min)

**answers**.		

**Debriefing meeting with note-taker**<br><br>Moderator & note-taker hold a short meeting immediately after the focus group<br>1) How did it go?<br>2) What are the most important results?<br>3) What things stood out?<br>4) What have we learned from today, from what we've heard?	To discuss outcomes, clear confusions, check interpretations	(Total duration 20 to 30 min)

INHERIT
Health | Equity | Environment

Appendix B Note-taking/Reporting form

Inter-Sectoral Health and Environment Research for Innovation Qualitative Evaluation WP5 - Focus Group Research

Use together with name coding form, and list of elements to pay special attention to at hand

**Pilot:**<br>**Date: .. / .. / ..**<br>**Time : …..:…..**<br>**Focus group moderator:**<br>**Note taker:**<br>**Checker of notes:**<br>**Number of attendees:**	**Reporting**<br><br>*Please note down who said what using coded letters b (person John Smith could be given code P1, the next person P2 etc.) This should be done beforehand.*<br>*Please also pay attention to statements regarding capability, opportunity and motivation*	**Observation:**<br><br>*Meant for field notes*<br>- *Your interpretation of what is happening*<br>- *Unusual things, what stood out, moods*<br>- *Gives context for your own notes*
**(1) Introduction and warming-up**<br><br>**min … - min…**		
**(2) Introductory questions**<br><br>**min … - min…**		
**(3) Key questions**<br><br>**(Intersectoral) Cooperation**<br><br>**min … - min…**<br><br>**Question 1. X Core factors that give life, what should be kept, preserved?**		

**(Intersectoral) Cooperation**<br><br>**min … - min…**<br><br>**Question 2. X What might have been, what might be**		
**(Intersectoral) Cooperation**<br><br>**min … - min…**<br><br>**Question 3. X Where want to be?**		
**(4) End/wrap up of Cooperation (what is most important, summarize, ask if accurate, missed anything?**<br><br>**min … - min…**		
**(5) Additional questions by partner**<br><br>**min … - min…**<br><br>**Enter Questions asked below:**		

**(6) Wrap up, thank you, final words, questions, remarks?**<br><br><br>**min … - min…**		

Post-it Notes Question	Answers from all participants
Describe a peak experience in (intersectoral) cooperation, when you felt really engaged and motivated.	
Where do you want to be between now and a certain period, what will it look like? What are possible options (actions, projects) to reach this and enhance cooperation in the future? Be ambitious! *(use provocative proposition: if your dream is X, where do you want to be in X?)*	

Appendix C Participant Name Coding Table

Inter-Sectoral Health and Environment Research for Innovation
Qualitative Evaluation WP5 - Focus Group Research

Pilot Project:
Note-taker:
Date of focus group:

**Name participant**	**Acronym participant**
	P1
	P2
	P3
	P4
	P5
	P6
	P7
	P8

Appendix D Note Checking Form

Inter-Sectoral Health and Environment Research for Innovation
Qualitative Evaluation WP5 - Focus Group Research

To be performed by other person, who was neither moderator nor note-taker.

Use the note-taking/ reporting form while listening to the audio file of the focus group

Purpose: Checking of reporting form and optionally making additional notes and observations

- **What has been written down, is it a good representation of what has been discussed in the focus group?**
- **Do you think the interpretation is correct, or do you have additional thoughts?**

Name Note checker:
Date:
Pilot project:

**Pilot:**<br>**Date: .. / .. / ..**<br>**Time : …..:…..**<br>**Focus group facilitator:**<br>**Note taker:**<br>**Checker of notes:**<br>**Number of attendees:**	**Reporting**<br><br>*Please note down who said what using coded letters b (person John Smith could be given code P1, the next person P2 etc.) This should be done beforehand.*<br>*Please keep the list of elements to pay special attention to at hand*	**Observation:**<br><br>*Meant for field notes*<br>- *Your interpretation of what is happening*<br>- *Unusual things, what stood out, moods*<br>- *Gives context for your own notes*

**(1) Introduction and warming-up min … - min…**		
**(2) Introductory questions min … - min…**		
**(3) Key questions**<br><br>**(Intersectoral) Cooperation min … - min…**<br><br>**Question 1. X Core factors that give life, what should be kept, preserved?**		

**(Intersectoral) Cooperation min … - min…**<br><br>**Question 2. X What might have been, what might be**		
**(Intersectoral) Cooperation min … - min…**<br><br>**Question 3. X Where want to be?**		
**(4) End/wrap up of Cooperation (what is most important, summarize, ask if accurate, missed anything?**<br><br>**min … - min…**		

**(5) Additional questions by partner min … - min…**<br><br>**Enter Questions asked below:**		
**(6) Wrap up, thank you, final words, questions, remarks?**		

Appendix E Participant Sign-in Sheet

Inter-Sectoral Health and Environment Research for Innovation
Qualitative Evaluation WP5 - Focus Group Research

Participant Sign-in Sheet

Date: / /2018	Moderator:	Pilot project:

Participant's Name	Signature

Appendix F Informed Consent Form

Inter-Sectoral Health and Environment Research for Innovation
Qualitative Evaluation WP5 - Focus Group Research

Focus group discussion on cooperation and implementation of interventions related to living, moving and consuming. The EU-funded project INHERIT (INter-sectoral Health and Environment Research for InnovaTion) aims to advance sustainable and healthy lifestyles by looking at the connection between human health, well-being and the surrounding environment. Xxx is one of the partners of this project and will carry out this focus group in xxx.

The objective of the focus groups is to collate information and gain insight into factors that help ensure successful cooperation within local interventions, by looking into what elements were supportive or posed barriers for cooperation between all the people involved.

For this research, you will participate in group discussions (6-8 participants) to answer questions regarding intersectoral cooperation related to living, moving and consuming.
During the study, you have the opportunity to learn possible ways to enhance current cooperation within this project. For your participation, you will receive a voucher of *[depends on country]* as compensation.

The research presents little or no risk to the participants, which is comparable to the risk encountered during an ordinary day of office work. Your participation is voluntary and you may withdraw from participation at any moment during the study without consequences of any kind. If you have any questions regarding the research before, during and after your research engagement, feel free to ask the research leader in the room, or to contact *[name local INHERIT coordinator]*, via email *[e-mail local INHERIT coordinator]*.

During the workshop, the discussion will be recorded and reported afterwards. The recording will be deleted after reporting and the report will be stored without any references to you. The anonymized data will be kept on the servers of <name partner and RIVM> and will be destroyed at the end of the project. Your personal information (name, address, etc.) will neither be registered nor stored. No names will be associated with the report, stories and quotes from the focus groups.
To guarantee anonymity of the other group participants, we urge you to handle their remarks anonymous and confidential and not discuss them outside the group discussion. Also, we want to stress that for this reason, we cannot guarantee complete anonymity.

This research is for academic purposes only and will not be shared to any other parties beyond the ones involved in this task of the project nor for commercial purposes.
For more information see our website: www.inherit.eu. Thank you for your participation!
By signing this consent form, I indicate I have read the information, and understand the information, and I give permission to use my anonymized input for the INHERIT project.
Date:
Name participant:
Signature:

Appendix G Invitation letter to focus groups

Inter-Sectoral Health and Environment Research for Innovation
Qualitative Evaluation WP5 - Focus Group Research

Dear sir/madam xxxx,

We would like to invite you to participate in a focus group on (intersectoral) cooperation within [pilot project X] on [date] in [venue, place of venue] . This focus group will be held to evaluate the cooperation process in the context of [pilot project x], in order to gain insight into success factors and barriers, contributing to knowledge on how intersectoral cooperation can take place successfully.
[if partners want to use the additional 30 minutes to discuss other topics or effect evaluation questions, please insert here the other topic that will be discussed]

Your input in this focus group would be very valuable and greatly appreciated. The focus group will take [1.30 hours/ 2 hours, depends on if additional 30 minutes are used]. Travel expenses will be reimbursed [in case of a gift voucher or small gift, optionally indicate this, especially for an invitation to a citizen]

This focus group is held in the context of the European project INHERIT, which is an innovative project that aims to contribute to health, sustainability, inequality by behavior and lifestyle change. The project aims to encourage people to modify their current lifestyles, characterized by 'take, make, dispose' models of growth. The project consists of 18 partners from around Europe, of which one is [your institute]. More information can be found at www.inherit.eu.

Throughout Europe, pilot projects have been selected that will be evaluated. [Project x] is one of these pilot projects.

Please let us know as soon as possible whether you are available on this date and if you are willing to participate in the focus group. We look forward to hearing from you.

Kind regards,

[name, profession, institute]

Appendix H COM-B elements

Inter-Sectoral Health and Environment Research for Innovation
Qualitative Evaluation WP5 - Focus Group Research

Please pay additional attention to the following topics in discussions:

Topic	Explanation of possible discussed elements of topic
**Com- B behavioral system**	Capability: abilities (physical or psychological) to perform certain actions/behavior regarding cooperation. Competences, skills, knowledge, expertise, experience.<br><br>Examples: communication skills, flexibility, openness, leadership/management, problem solving skills, planned action, awareness
	Opportunity: Elements from the physical or social environment that may hinder or facilitate actions/behaviors<br><br>Examples: trust, social support, (technical/political) support from other parties, social cohesion, networks, diversity of participants, (quality of) communication, mutual dependency, voluntary or paid, resources, time, agreement on roles, community context and readiness, linkages to other groups, shared values
	Motivation: certain intentions for behaviors/actions, attitudes, habits, conscious reflective decisions, wants, desires, needs<br><br>Examples: attitudes towards cooperation, satisfaction, perceived effectiveness, perceived benefits and costs, engagement, perceived need for cooperation, personal values, dedicated people

Appendix I Checklist for Coordinator of Focus group

Inter-Sectoral Health and Environment Research for Innovation
Qualitative Evaluation WP5 - Focus Group Research

Checklist BEFORE the focus group

Knowledge
- □ Have you read the entire handbook?
- □ Have you followed the webinar and completed the test?

Date
- □ Have you checked possible dates with participants?
- □ Have you selected the one with highest availability/ availability of most essential people?
- □ Are you (coordinator) available on the date of the focus group?
- □ Are your moderator and note-taker available on the date of the focus group?

Venue
- □ Have you arranged a suitable venue?
- □ Have you arranged catering (tea, coffee, optional snacks, water)?

Team
- □ Have you selected and confirmed a moderator?
- □ Have you selected and confirmed a note-taker?

Participants
- □ Have you invited all participants?
- □ Have you slightly over recruited participants (in case of drop out/ no show)?
- □ Have you reminded all participants?

Translation of materials
- □ Have you translated the moderator script?
- □ Have you translated the note-taking form?
- □ Have you translated the informed consent form?
- □ Have you translated the participants sign-in sheet?

Printing of materials
- □ Handbook including this checklist
- □ Moderator script x3 (for coordinator, moderator, note-taker)
- □ Note taking form (also when doing digital note-taking, as back-up)

- ◻ Participant name coding table
- ◻ Special attention topics sheet for moderator and note-taker
- ◻ Consent form for all participants
- ◻ Participant sign-in sheet

Focus group materials/ logistics

- ◻ Have you arranged two audio taping devices?
- ◻ Have you arranged enough sticky notes?
- ◻ Have you arranged enough pens?
- ◻ Have you arranged paper for name tents or cards?
- ◻ Have you arranged two timers for moderator and note-taker? (stopwatches for example)
- ◻ Have you arranged a laptop for the note-taker (in case of digital note-taking)

Checklist on the day of the focus group

- ◻ Make sure material is installed: audio taping material, sticky notes and pens, name tents, enough informed consent forms, sign-in sheet, 2timers
- ◻ Material for coordinator: checklist coordinator, handbook, moderator script
- ◻ Material for note-taker: noteblock, pen, note-taking forms, **PREFERABLY A LAPTOP with note taking form on it,** name coding table, paper to draw seating map, moderator script, attention elements sheet
- ◻ Material for moderator: moderator script, note block and pen

- ◻ Arrange and set up refreshments
- ◻ Arrange the chairs and table so everyone can see each other
- ◻ Set up all equipment, verify it is working properly
- ◻ Welcome participants as they arrive
- ◻ Sit outside circle, opposite moderator.
- ◻ If late participant: take them outside, explain what has been discussed up till now, show him where to sit
- ◻ Observe the discussion, make notes of well-said quotes, non-verbal activity
- ◻ Monitor recording equipment
- ◻ Do not participate in the discussion!

Appendix J Checklist for moderator of the focus group

Week before focus group

- ◻ Complete (relevant parts of or entire) webinar
- ◻ Go through the handbook and relevant appendices
- ◻ Practice introduction without referring to notes
- ◻ Practice questions and be familiar with the key questions

On day of focus group, before it takes place

- ◻ Be present at least 30 minutes before the start, to receive everyone
- ◻ Put chairs in U shape/ round shape
- ◻ Seating plan for all participants
- ◻ Participant sign-in sheet visibly near entrance
- ◻ Check beforehand with coordinator if participants will be given a reward (voucher, or small gift), or just compensation for their travel expenses, to be able to inform participants after the focus group

During focus group

- ◻ Create a comfortable open atmosphere
- ◻ Have all participants signed the informed consent form?
- ◻ Have all participants signed the sign-in sheet?
- ◻ TURN ON audio devices
- ◻ Has everyone had a chance to share?
- ◻ Avoid giving personal opinions, or approval verbal comments
- ◻ Use probes and pauses
- ◻ Pens and sticky notes distributed
- ◻ Have participants written Q1 or Q3 on the sticky notes answers?
- ◻ Collected all sticky notes

After the focus group

- ◻ Have participants received the rewards for participating?
- ◻ Have you had a debriefing meeting with the note-taker after the focus group?
